# The significance of pyrogenic polycyclic aromatic hydrocarbons in Borneo peat core for the reconstruction of fire history

**DOI:** 10.1371/journal.pone.0256853

**Published:** 2021-09-08

**Authors:** Sher-Rine Kong, Masanobu Yamamoto, Hasrizal Shaari, Ryoma Hayashi, Osamu Seki, Norhayati Mohd Tahir, Muhammad Fais Fadzil, Abdullah Sulaiman

**Affiliations:** 1 Faculty of Science and Marine Environment, Universiti Malaysia Terengganu, Kuala Terengganu, Malaysia; 2 Faculty of Environmental Earth Science, Hokkaido University, Sapporo, Japan; 3 Graduate School of Environmental Earth Science, Hokkaido University, Sapporo, Japan; 4 Lake Biwa Museum, Kusatsu, Japan; 5 Institute of Low Temperature Science, Hokkaido University, Sapporo, Japan; 6 Institute of Oceanography and Environment, Universiti Malaysia Terengganu, Kuala Terengganu, Malaysia; 7 Department of Mineral and Geoscience, Kedah, Kuala Terengganu, Malaysia; National Dong Hwa University, TAIWAN

## Abstract

The reconstruction of fire history is essential to understand the palaeoclimate and human history. Polycyclic aromatic hydrocarbons (PAHs) have been extensively used as a fire marker. In this work, the distribution of PAHs in Borneo peat archives was investigated to understand how PAHs reflect the palaeo-fire activity. In total, 52 peat samples were analysed from a Borneo peat core for the PAH analysis. Pyrogenic PAHs consist of 2–7 aromatic rings, some of which have methyl and ethyl groups. The results reveal that the concentration of pyrogenic PAHs fluctuated with the core depth. Compared to low-molecular-weight (LMW) PAHs, the high-molecular-weight (HMW) PAHs had a more similar depth variation to the charcoal abundance. This finding also suggests that the HMW PAHs were mainly formed at a local fire near the study area, while the LMW PAHs could be transported from remote locations.

## Introduction

Polycyclic aromatic hydrocarbons (PAHs) are commonly found in the atmosphere, marine, and terrestrial areas, including peats, sediments, ancient sedimentary rocks, and petroleum [1–4]. The parent PAHs (PAH molecules with no alkyl substitutes) and some alkylated PAHs are produced from the incomplete combustion of organic matter in the environment [5–7], while alkylated PAHs are formed mainly from the sedimentary organic matter in sedimentary rocks and petroleum during thermal maturation [8] and petrogenic [9] processes.

The reconstruction of past fire events is critical to understand the carbon cycle, climatic regime [10, 11], fire source [12], and vegetation change [13]. The records of PAHs in sedimentary archives have been used as tracers for past fire events [14]. The chemical composition of pyrogenic PAHs is generally varied and depends on their sources (combustion materials) such as grass and fossil fuels [15, 16] and combustion temperature [17, 18]. PAHs can be long-range transported as aerosols in the atmosphere [19]. Low-molecular-weight PAHs are more volatile and water-soluble than high-molecular-weight PAHs [20, 21]. Therefore, the chemical composition of the PAHs in sedimentary archives can offer useful information on the material source, combustion temperature, and transportation process [22, 23]. However, the way of interpretation of PAH compositions is not established in the practical use of reconstruction of fire events in sedimentary (peat) archives.

Generally, many factors can affect the concentration and composition of PAHs, and most factors are complex to analyse in modern samples because there are contributions of additional anthropogenic sources such as petroleum-derived PAHs and the combustion of fossil fuels. Thus, the chemical composition of PAHs should be investigated and analysed in a simple environmental setting to understand the contributing factors that determine the characteristics of PAHs.

In this paper, we investigated the PAHs in the peat samples obtained from the summit of the Baram peat dome in northern Borneo. The Baram peatland was covered with natural vegetation until the end of the 20^th^ century, and human activity was minimal in the peat dome. By comparing with the charcoal abundance record [24], we also discussed the factors that controlled the PAH distribution.

**Fig 1:**
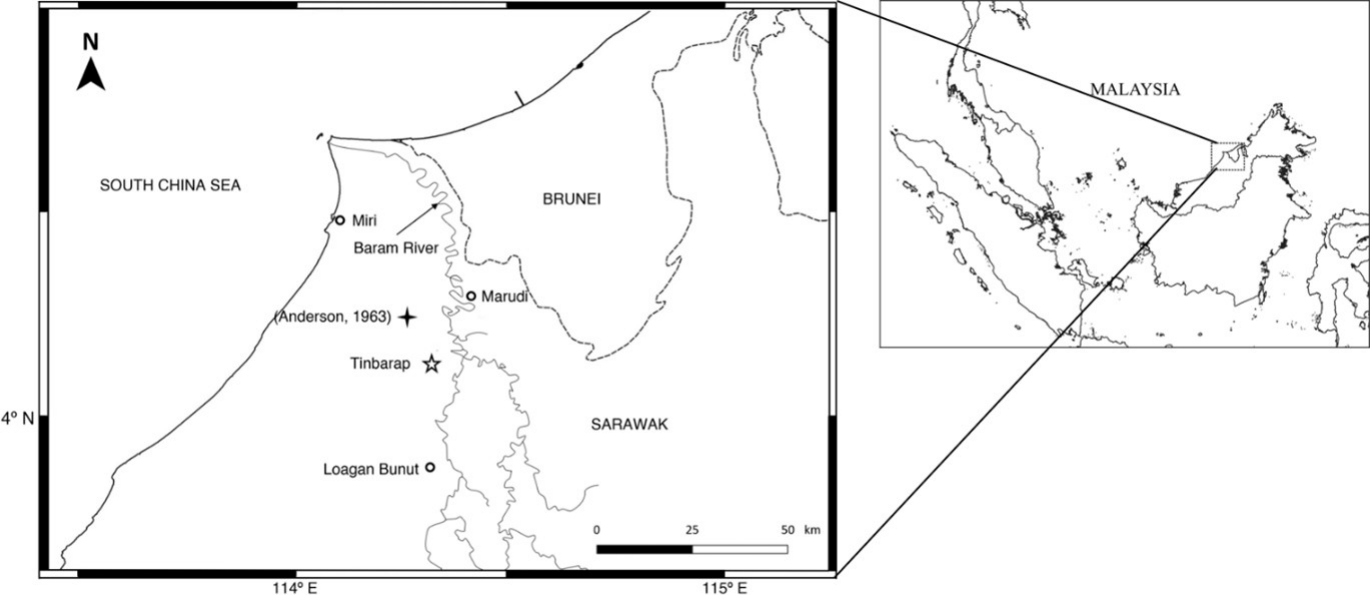
Map of the studied site of Borneo tropical peatland. A peat core was collected at the Tinbarap site (N 04°03’01.47” E 114°15’02.45”), which was marked as ☆. A previous study was conducted by Anderson [29] (marked by 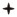) on the vegetation composition of the Borneo peat dome. The map was made using QG is software (version 3.6.2).

Borneo is an island within the Western Pacific Warm Pool (WPWP) with a tropical climate and high year-round temperature and precipitation. The precipitation is influenced by both the East Asian Winter Monsoon (EAWM) and El Niño-Southern Oscillation (ENSO). Today, the ENSO regulates the frequency of forest fires in Borneo [25]. In El Niño years, the frequency of forest fires is significantly high due to low precipitation (<100 mm per month in dry seasons) and dry environments [25–27]. The monthly precipitation reduction by >80 mm during August-October significantly increased the fire activity in southern Borneo in El Niño years during 1997–2015 (Fig 1D) [27]. Thus, fire history provides a precipitation record in Borneo [24], which is influenced by the EAWM and ENSO.

## Samples and methods

### Study cores

**Fig 2:**
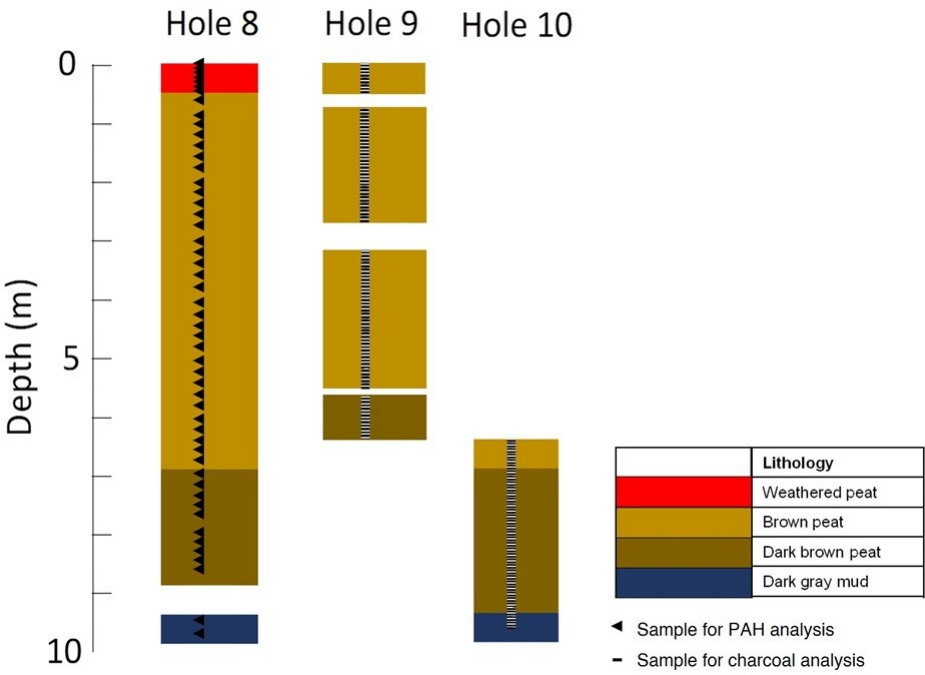
The lithology of Borneo peat core with a total length of 10 m. The selected intervals for analysis are indicated by black triangles (◄), and charcoals were measured in every 2 cm-interval, indicated by dash lines (˗). The difference between brown and dark brown colours is gradual and not significant.

The peat cores were retrieved from three holes at the Tinbarap site near Marudi Town, Sarawak, Malaysia (N 04°03’01.47” E 114°15’02.45”, Fig 1). The permit for collection of Tinbarap peat core was required and obtained from the Sarawak Forestry Corporation and the Sarawak Biodiversity Centre. Hole 8 was drilled using a Russian peat sampler, whereas Holes 9 and 10 were drilled using a thin-wall sampler (Fig 2). Holes 8, 9, and 10 were drilled at about the same point. Each hole was located far from the other hole within one meter. The peats are thus correlative based on the depth. The site was located at the summit of the Baram peat dome [28]. Anderson [29] described six phasic vegetation communities that formed between the marginal and central areas of the peat dome: *mixed swamp forest*, *Alan Forest*, *Alan Bunga Forest*, *Padang Alan* (*Padang Medang*), *Padang Paya*, and *Padang Keruntum*. The *Padang Paya* and *Padang Keruntum* communities were reported as the most significant biodiversity-ecosystems, which can only be found between the midst of the Baram dome to the upriver of Marudi [30]. The Baram peat dome started to develop more than 5,300 years ago. There were approximately 18,920 ha of undisturbed peatlands. The natural vegetation at the study site was oligotrophic low trees [29] and recently cleared by the development of oil palm plantation [30]. The core sediments consist of weathered peat (0–0.4 m), brown to dark brown peat (0.4–9.5 m), and dark grey mud (9.5–9.8 m). The peat contained undecomposed roots and fragments of woods, leaves, and charcoals. Microscopic observation showed that plant tissues are not degraded throughout the holes of the site. This suggests that the preservation of organic matter was unchanged remarkably. Charcoals can be found throughout the entire hole, and the charcoal abundance markedly varied every approximately 2 metres.

Eight samples from Holes 9 and 10 were dated at the Tinbarap site (S8 Fig in S1 File). The remains of leaves in the peats were picked out and prepared using the acid-alkali-acid (AAA) treatment [31]. The samples were combusted with CuO at 850°C for 3 hours in a sealed quartz glass tube to produce CO_2_, which was then purified in liquid N_2_ and EtOH-liquid N_2_ traps [32]. The purified CO_2_ was reduced to graphite with an iron powder catalyst. The graphite was analysed at the accelerator mass spectrometry facility at the Museum of the University of Tokyo. Conventional ages were converted to calendar ages using the OxCal program (version 4.4) [33] and the IntCal20 dataset [34]. The age of the bottom peat layer was 5,321 years BP. The average sedimentation rate was ~0.17 cm year^−1^. The age of the core-top sample was obtained by extrapolation of the age-depth relationship for the first and second top dates.

### Lipid extraction and fractionation

The samples were freeze-dried and homogenised. The PAHs were extracted and fractionated according to the following method. Approximately 1 g of sample was extracted thrice with 100 mL of dichloromethane/methanol (3:1 v/v) for 45 minutes using an ultrasonic agitation approach. The extract was condensed to 3 mL and saponified by adding 1 mL of 6% potassium hydroxide in methanol-water (4:1) and 1 mL of clean Mili-Q water. The extract was heated at 80°C in a water bath for an hour. The saponified lipid was extracted with *n-*hexane three times before it was fractionated by column chromatography with silica gel. Before usage, the silica gel was heated at 200°C for 4 hours and cleaned Mili-Q water was added (5% weight of silica gel). The column consisted of 12 mL of slurry silica gel (Gel 60 40–60 μm mesh) with a little sodium sulfate powder added to the top. The lipid was separated into three fractions: *n-*hexane (1:1 v/v) 8 mL (*n*-alkane); *n-*hexane/dichloromethane (9:1, v/v) 8 mL; *n-*hexane/dichloromethane (1:1, v/v) 7 mL (PAH) and dichloromethane/methanol (9:1, v/v) (sterol). The *n-*hexane/dichloromethane (9:1, v/v) and *n-*hexane/dichloromethane (1:1, v/v) fractions were combined and analysed using a gas chromatography-mass spectrometer (GC-MS).

### PAH analysis

PAHs were measured using a GC-MS (Shimadzu-QP2010 Ultra) fitted with a DB-5MS fused silica capillary column (30.0 m length x 0.25 mm i.d; 0.25 μm film thickness). The mass spectrometer was operated at a full scan in the electron impact mode (70 eV) with a mass range of m/z 45–600 at a scan speed of 1.25 s. Helium (>99.9% purity) was used as a carrier gas with a constant pressure of 67.0 kPa. The splitless injector, ion source, and interface temperatures were set at 300°C, 200°C, and 300°C, respectively. The column temperature was programmed as follows: Hold for 1 min at 50°C; apply a temperature ramp from 50 to 300°C at 5°C/min; maintain at 300°C for 20 minutes. This process resulted in a total run time of 74 minutes.

As listed in Tables 1 and 2, the PAH compounds were identified by comparing their mass spectra and retention times with those of available compounds in the standard. Those compounds (Table 1) that are available in the standard were obtained from Supleco, USA and dissolved with dichloromethane/hexane solvent mixture. To measure the reproducibility of the analysis, the signal-to-noise (S/N) ratio for each available compounds were measured by integrating the signal of the compound to the noise approximately 1 minute before and after of the compound peak area appeared in the mass chromatogram and multiply the signal by 3. Table 3 shows the detection limit and reproducibility of the available compounds in the standard. The unavailable alkylated compounds were identified by comparing the mass spectra and retention times in Mita and Shimoyama [35], Marynowski et al. [36], Mita [37], Romero-Sarmiento et al. [38], and Romero-Sarmiento et al. [39].

**Table 1:** Measured polycyclic aromatic hydrocarbons (PAHs) identified based on the authentic reference compounds.

Peak	Compounds	Code	Molecular weight (m/z)	Response factor
1	Naphthalene	Naph	128	9.73E-06
2	Fluorene	Flu	166	1.86E-05
3	Phenanthrene	Phe	178	1.30E-05
4	Anthracene	Ant	178	1.37E-05
5	Fluoranthene	Fla	202	1.57E-05
6	Pyrene	Py	202	1.54E-05
7	Benzo[a]anthracene	BaA	228	3.57E-05
8	Chrysene	Chr	228	3.17E-05
9	Retene	Ret	234	1.01E-04
10	Perylene	Per	252	7.24E-05
11	Indeno[1,2,3-c,d]pyrene	IPy	276	1.93E-04
12	Benzo[g,h,i]perylene	BPer	276	1.29E-04
13	Dibenzo[a,h]anthracene	DiA	278	3.72E-04

**Table 2:** List of the measured polycyclic aromatic hydrocarbons (PAHs) identified based on the retention times and mass spectra published by Mita and Shimoyama [35], Marynowski et al. [36], Mita [37], Romero-Sarmiento et al. [38], and Romero-Sarmiento et al. [39].

Peak	Compounds	Code	Molecular weight (m/z)	Response factor
14	2-Methylnaphthalene	2-MN	142	9.73E-06
15	1-Methylnaphthalene	1-MN	142	9.73E-06
16	Biphenyl	Bip	154	9.73E-06
17	1,3-Dimethylnaphthalene	1,3-DMN	156	9.73E-06
18	1,6-Dimethylnaphthalene	1,6-DMN	156	9.73E-06
19	1,4- + 2,3-Dimethylnaphthalene	1,4- + 2,3-DMN	156	9.73E-06
20	1,5-Dimethylnaphthalene	1,5-DMN	156	9.73E-06
21	1,2-Dimethylnaphthalene	1,2-DMN	156	9.73E-06
22	1,8-Dimethylnaphthalene	1,8-DMN	156	9.73E-06
23	2,6 + 2,7-Dimethylnaphthalene	2,6- + 2,7-DMN	157	9.73E-06
24	2-Methylbiphenyl	2-MBip	168	9.73E-06
25	Diphenylmethane	DMe	168	9.73E-06
26	3- + 4-Methylbiphenyl	3- + 4-MBip	168	9.73E-06
27	1,3,7-Trimethylnaphthalene	1,3,7-TMN	170	9.73E-06
28	1,3,6-Trimethylnaphthalene	1,3,6-TMN	170	9.73E-06
29	1,4,6- + 1,3,5-Trimethylnaphthalene	1,4,6- + 1,3,5-TMN	170	9.73E-06
30	2,3,6-Trimethylnaphthalene	2,3,6-TMN	170	9.73E-06
31	1,2,7-Trimethylnaphthalene	1,2,7-TMN	170	9.73E-06
32	1,6,7-Trimethylnaphthalene	1,6,7-TMN	170	9.73E-06
33	1,2,6-Trimethylnaphthalene	1,2,6-TMN	170	9.73E-06
34	1,2,4-Trimethylnaphthalene	1,2,4-TMN	170	9.73E-06
35	1,2,5-Trimethylnaphthalene	1,2,5-TMN	170	9.73E-06
36	1,4,5-Trimethylnaphthalene	1,4,5-TMN	170	9.73E-06
37	2-Butylnaphthalene	2-BN	170	9.73E-06
38	Tetramethylnaphthalene	TeMN	184	9.73E-06
39	3-Methylphenanthrene	3-MP	192	1.30E-05
40	2-Methylphenanthrene	2-MP	192	1.30E-05
41	2-Methylanthracene	2-MA	192	1.37E-05
42	4- + 9-Methylphenanthrene	4- + 9-MP	192	1.30E-05
43	1-Methylphenanthrene	1-MP	192	1.30E-05
44	Cadalene	Cad	198	9.73E-06
45	3-Ethylphenanthrene	3-EP	206	1.30E-05
46	2- + 9-Ethylphenanthrene	2- + 9-EP	206	1.30E-05
47	1-Ethylphenanthrene	1-EP	206	1.30E-05
48	3,5- + 2,6-Dimethylphenanthrene	3,5- + 2,6-DMP	206	1.30E-05
49	2,6- + 2,7-Dimethylphenanthrene	2,6- + 2,7-DMP	206	1.30E-05
50	1,3- + 2,10- + 3,9- + 3,10-Dimethylphenanthrene	1,3- + 2,10- + 3,9- + 3,10-DMP	206	1.30E-05
51	1,6- + 2,9- + 2,5-Dimethylphenanthrene	1,6- + 2,9- + 2,5-DMP	206	1.30E-05
52	1,7-Dimethylphenanthrene	1,7-DMP	206	1.30E-05
53	2,3- + 1,9-Dimethylphenanthrene	2,3- + 1,9-DMP	206	1.30E-05
54	1,8-Dimethylphenanthrene	1,8-DMP	206	1.30E-05
55	1,2,3-Trimethyl-4-propenylnaphthalene	1,2,3-TMPN	210	9.73E-06
56	2-Methylpyrene	2-MPy	216	1.54E-05
57	4-Methylpyrene	4-MPy	216	1.54E-05
58	1-Methylpyrene	1-MPy	216	1.54E-05
59	Simonellite	Sim	237	1.37E-05
60	Methylchrysene	MChr	242	3.17E-05
61	Coronene	Cor	300	1.29E-04

**Table 3:** The detection limits and reproducibility (standard deviation) of the available PAHs in the standard.

Compounds	Detection limit (μg/g)	Standard deviation
Naphthalene	1.76	3.96E-01
Fluorene	1.66	3.34E-01
Phenanthrene	11.93	3.86E+00
Anthracene	3.39	7.34E-01
Fluoranthene	5.72	1.40E+00
Pyrene	4.53	1.08E+00
Benzo[a]anthracene	3.42	1.49E+00
Chrysene	15.22	9.30E+00
Retene	1.12	7.01E-01
Perylene	2.52	1.07E+00
Indeno[1,2,3-cd]pyrene	0.75	1.89E-01
Benzo[g,h,i]perylene	1.40	3.62E-01
Dibenzo[a,h]anthracene	1.37	7.12E-01

Quantification was done by the comparison between samples and a standard mixture in different runs. The concentration of the PAH compound (Eq 1) was calculated according to the peak area of the compound, response factor, and sample weight as follows:

$$\text{Concentration}\;\text{in}\;\mu \text{g}/\text{g}=\text{Response}\;\text{factor}\;\text{x}\;\text{Peak}\;\text{area}\;\text{of}\;\text{sample}\;\text{x}\;\frac{\left(\frac{\text{Final}\;\text{fraction}\;\text{volume}}{\text{Injection}\;\text{volume}}\right)}{\text{Sample}\;\text{weight}\;(\text{g})}$$ (1)

The response factor was obtained by the analysis of known amounts of the PAH mixture (Table 1). The unavailable compounds were assumed to have identical response factors to the compounds with a similar molecular weight because the response factor strongly depends on the molecular weight (Table 2).

### Principal component analysis

A principal component analysis (PCA) was performed for the dataset of PAH concentrations using a PRIMER-E (PRIMER 6 version 6.1.12) software. The PAH concentration was normalised based on the mean and standard deviation and converted to the z-score before the PCA was performed [40].

## Results

**Fig 3:**
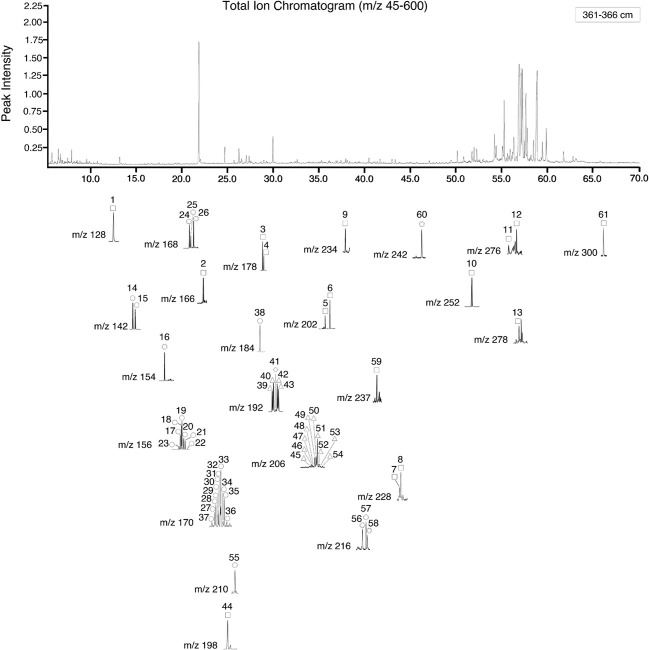
Total ion chromatogram (TIC) and the mass chromatograms of the molecular ions of the PAHs in sample Hole 8, 361–366 cm. The numbered spectral peaks are the compounds listed in Tables 1 and 2.

**Fig 4:**
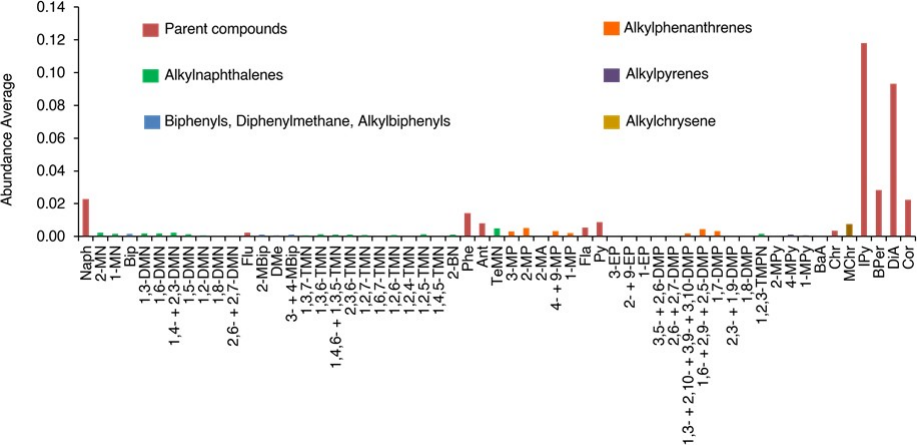
The relative abundance of pyrogenic compounds averaged in all samples.

**Fig 5:**
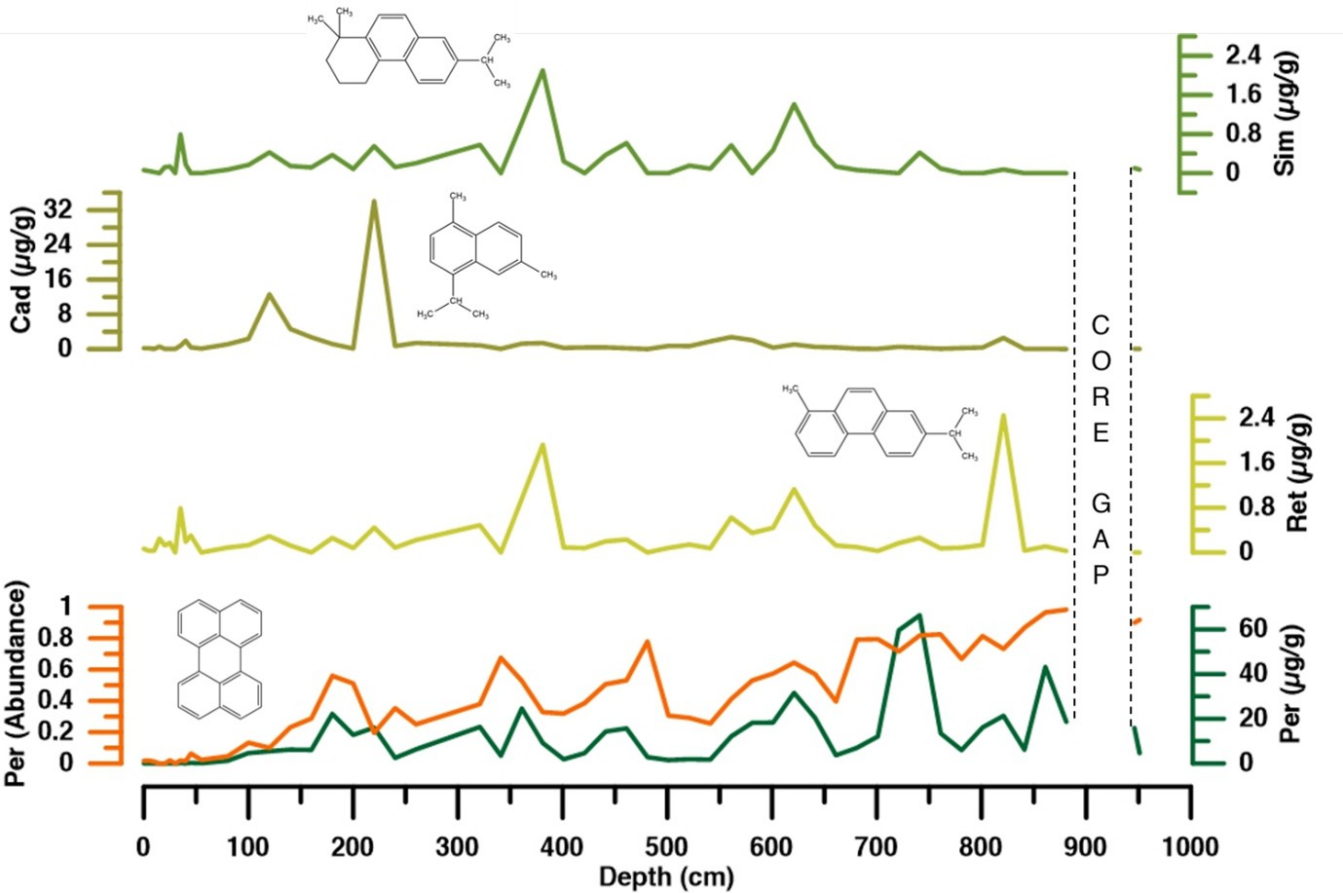
Depth variations in retene (Ret), perylene (Per), cadalene (Cad), and simonellite (Sim) concentrations.

In total, 61 individual compounds, including parent and alkylated compounds, were identified in Tinbarap peat samples (Tables 1 and 2 and Fig 3). Their chemical structures are shown in S1 Appendix in S1 File. The identified PAHs ranged from two (naphthalene) to seven (coronene) condensed aromatic rings: naphthalene (m/z 128), fluorene (m/z 166), phenanthrene (m/z 178), anthracene (m/z 178), fluoranthene (m/z 202), pyrene (m/z 202), benzo[a]anthracene (m/z 228), chrysene (m/z 228), indeno[1,2,3-c,d]pyrene (m/z 276), benzo[g,h,i]perylene (m/z 276), dibenzo[a,h]anthracene (m/z 278), and coronene (m/z 300). Our careful investigation of ion chromatograms indicates that there were alkylated homologs (Fig 4) in naphthalene, phenanthrene, pyrene, and chrysene structures, but they were absent in fluorene, fluoranthene, benzo[a]anthracene, indeno[1,2,3-c,d]pyrene, benzo[g,h,i]pyrene, perylene, dibenzo[a,h]anthracene, and coronene. Among these compounds, retene (m/z 234), perylene (m/z 252), cadalene (m/z 198), and simonellite (m/z 237) (Fig 5) are generally interpreted as “diagenetic” compounds [41–44]. Other compounds are considered the PAHs of pyrogenic origin [15, 45, 46]. Major pyrogenic PAHs are naphthalene, fluorene, phenanthrene, anthracene, fluoranthene, pyrene, benzo[a]anthracene, chrysene, retene, perylene, indeno-[1,2,3-c,d]pyrene, and dibenzo[a,h]anthracene (S1 Fig in S1 File).

Retene, perylene, cadalene, and simonellite are generally considered as “diagenetic” compounds. The concentrations of “diagenetic” compounds were 0.04–67.33 μg/g with an average of 13.25 μg/g (Fig 5). Perylene was the most abundant compound among the “diagenetic” compounds. The relative abundance of perylene to the total PAHs markedly increased with increasing core depth (Fig 5). Other “diagenetic” compounds show a cyclic variation.

**Fig 6:**
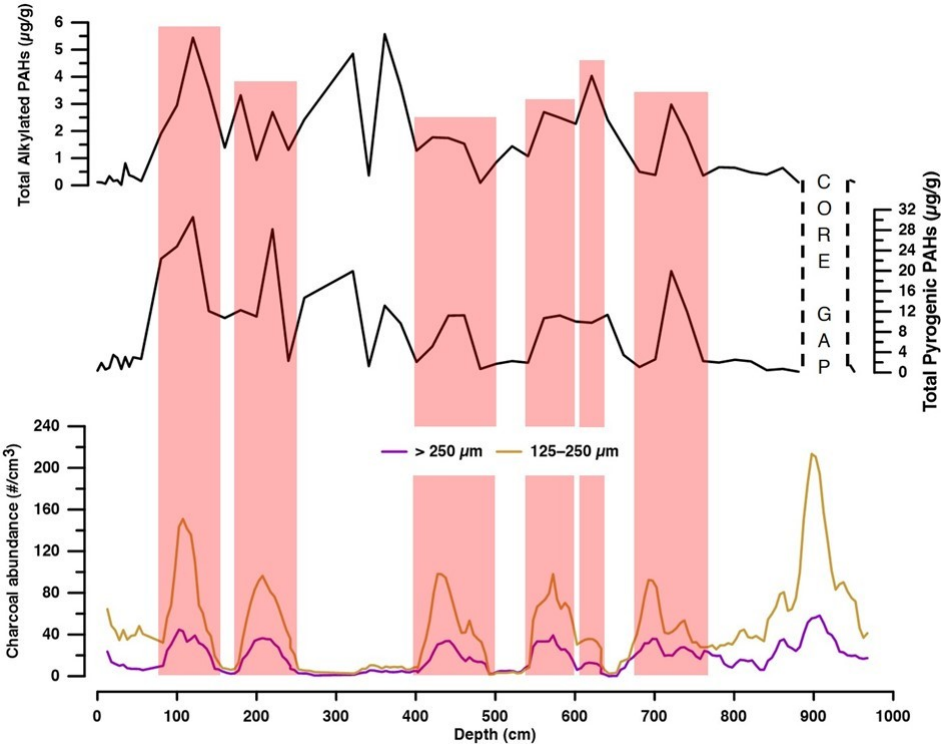
Depth variations in charcoal abundance with the total concentrations of pyrogenic parent and alkylated PAHs.

The total concentrations of “pyrogenic” compounds in the Tinbarap peat core significantly fluctuated in the range of 0.17–30.55 μg/g with an average of 7.19 μg/g (Fig 6). The variation in the total concentration of alkylated PAHs mirrors the total concentration of “pyrogenic” PAHs (Fig 6). Depth variations in all “pyrogenic” PAHs are shown in S1 Fig in S1 File.

**Fig 7:**
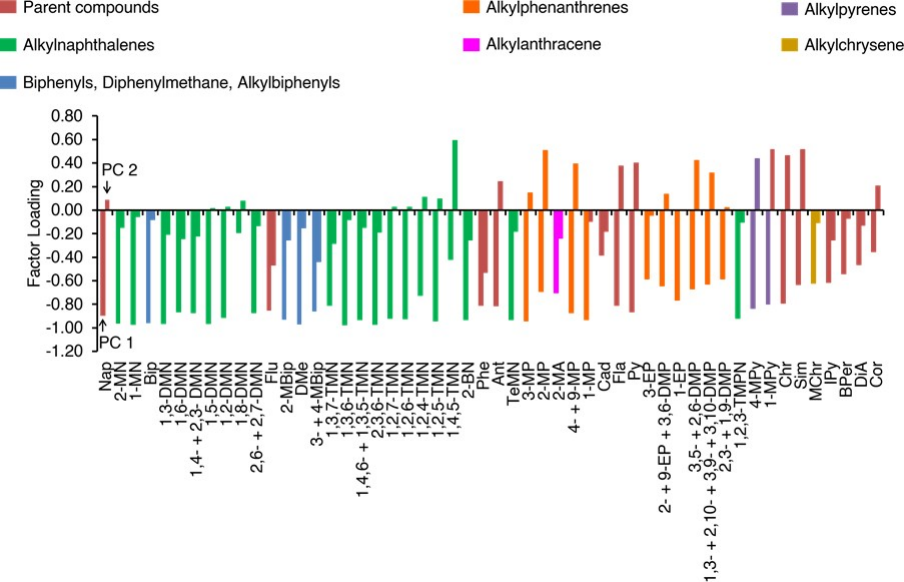
Factor loadings of PC1 and PC2 of PAH compounds.

**Fig 8:**
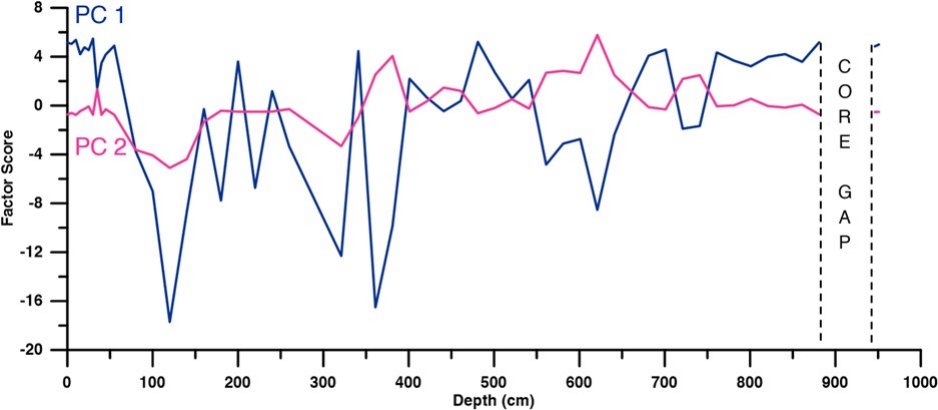
Depth plots of factor scores for PC 1 and PC 2.

The PCA analysis (Figs 7 and 8) extracts two major factors that determine the concentrations of “pyrogenic” PAHs; PC 1 (65.0% of the total variance) and PC 2 (7.5% of the total variance). PC 1 explained the synchronous variations of pyrogenic PAHs, which implies that the variation of all pyrogenic PAHs was identical to the first-order approximation. PC 2 explained the difference in depth distributions between high- and low-molecular-weight PAHs. Methylated and ethylated PAHs were accompanied by their parent molecules.

## Discussion

### Diagenetic formation of perylene

Perylene was found to be a major PAH in Tinbarap peat samples. The relative abundance of perylene in total PAHs markedly increased with increasing depth (Fig 5). Perylene is assumed to be formed from the biological pigment 4,9-dihydroxiperylene-3,10-quinone, biosynthesised by fungi, insects, and marine organisms that formed by the reduction in anaerobic sediments [18, 47–49], soils [47, 50, 51], and termite nests [52]. The downward-increasing trend of perylene abundance in the Tinbarap peat core can be attributed to the diagenetic formation of perylene with increasing burial depth.

### Molecular weight of pyrogenic PAHs

**Fig 9:**
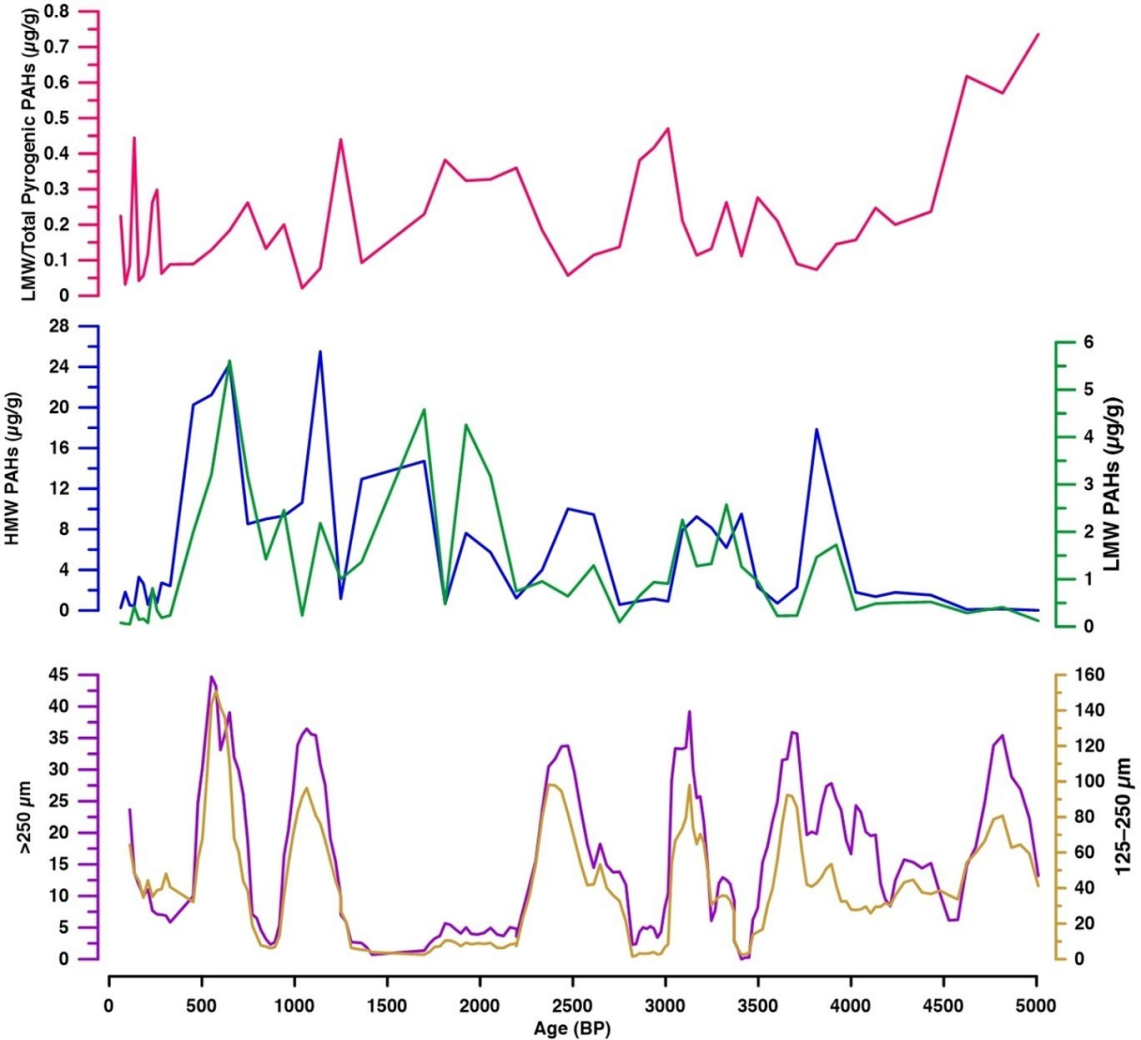
Variations of low-molecular-weight and total pyrogenic PAHs (LMW/Total pyrogenic PAHs), high-molecular-weight (HMW), and low-molecular-weight (LMW) PAHs with comparison to the charcoal abundance (5-point moving mean).

The difference in depth variation between compounds with 2–3 ring (LMW) and those with 5–6 ring (HMW) (Fig 9) suggests that the pyrogenic PAHs in Tinbarap peat core has two different groups, indicated by the PC 2 variation (Fig 8). McGrath et al. [53] showed that LMW PAHs (fluorene, phenanthrene, and anthracene) were usually generated in a broader temperature range than HMW PAHs (indeno[1,2,3-c,d]pyrene, dibenzo[a,h]anthracene, and benzo[g,h,i]perylene) by the pyrolysis of cellulose. Thus, the difference in pyrolysis temperature can affect the variation of PAHs in the peat archives.

Alternatively, the difference in transportation among PAHs can affect the change in PAH composition in the peat archives. The LMW PAHs are more volatile than HMW PAHs [20, 54]. In comparison to LMW PAHs, the depth variation of HMW PAHs was more similar to the variation of charcoal abundance (Fig 9). The HMW PAHs variation show a statistically significant correlation with charcoal abundance (r = 0.40 and p = 0.006 for both >125 μm and 250 μm charcoal fractions). In contrast, LMW PAH variation show a much lower correlation with charcoal abundance (r = 0.15 and 0.13; p = 0.30 and 0.40 for >125 μm and 250 μm fractions, respectively). Charcoals are residues of combusted plant tissues [55]. Macroscopic charcoals with particle sizes of >125 μm and 250 μm were prominent local fire tracers [56, 57]. In-situ or local fires produced more charcoal than the ex-situ or distal fire [58–60]. Previous studies have shown that larger macroscopic charcoals were formed at and near the burnt areas [57, 58, 61]. The dispersion distance of char particles (<200 μm) is at a maximum of 10 km [12]. Hence, charcoal cannot be transported far. Therefore, HMW PAHs reflect the local fire, while the LMW PAHs capture fire events from remote locations. Karp et al. [23] showed that LMW PAHs are significantly enriched in smokes (<2.5 μm) than in char residues [23], suggesting that the LMW/Total pyrogenic PAHs ratio is an indicative of the transportation of PAHs. The LMW/Total pyrogenic PAHs ratio varied reversely with charcoal abundance (Fig 9). Our result of the close relationship between charcoal and HMW PAHs strongly supports the fidelity of their proposed index. This implies that the HMW PAHs can be used as a proxy of local fire activity as charcoal is. PAHs are generally analysed together with other biomarkers, potentially providing more data for fire activity to reconstruct more robust fire history. On the other hand, the usage of LMW PAHs for the reconstruction of proximal fires needs caution, although these compounds can be potentially used for the reconstruction of regional fire history.

Our PAH record indicates that fire activity was intensified at several hundred years interval (Fig 9). Usually, natural tropical rainforest is highly resistant to fire because of low dry fuel availability and high humidity, even during drought [25]. Thus, the repeated occurrence of wildfires implies that the megadroughts have occurred repeatedly in Borneo peatlands in the past. The preliminary data of charcoal at five different sites were discussed in the context of climate variability [24], showing the influence of solar cycles affecting the occurrence of droughts in Borneo. Further PAH studies will be useful to test the hypothesis.

### Thermal origin of retene, simonellite, and cadalene

Generally, retene and simonellite are considered as “diagenetic” compounds, since they are formed from diterpenoids of higher plant resins in gymnosperms during diagenesis of sediment burial [62, 63]. However, the peat samples did not have sufficient time to thermally mature before forming “diagenetic” compounds. There is no increasing downward trend of retene concentration, not supporting the diagenetic formation of retene. The depth variations of these compounds are similar to those of some pyrogenic PAHs such as pyrene, benzo[a]anthracene, and fluoranthene (Fig 5 and S1 Fig in S1 File). The pyrolysis of peat yield retene transformed from diterpenoids by heating of fires [64]. The retene in ice cores [65, 66], air [67], and lake sediments [68] were often attributed to the combustion of plants. Retene in the Tinbarap peat core showed similar variation patterns to that of charcoal abundance (Figs 5 and 6). Thus, these compounds were formed by the pyrolysis of plants and considered as thermally transformed compounds.

Cadalene is also well-known for its “diagenetic” characteristics. Cadalene belongs to a sesquiterpenoid derived from angiosperm plants [69]. Cadalene is formed from the degradation of cadinene and cadinol during diagenesis [41]. However, the peat samples did not have sufficient time to thermally mature before forming “diagenetic” compounds. There is no increasing downward trend of cadalene concentration, not supporting the diagenetic formation of cadalene. Thus, like retene, cadalene can also be a thermally-transformed compound formed by the combustion of angiosperm plants. Cadalene in Tinbarap peat core showed a unique depth variation, which probably reflects the contribution of angiosperm to fuel materials for wildfires.

## Conclusion

The pyrogenic PAHs in Tinbarap peats had 2–7 rings, where some compounds had methyl and ethyl groups. Pyrogenic PAHs showed large fluctuations with the core depth. Compared to low-molecular-weight (LMW) PAHs, the depth variation of high-molecular-weight (HMW) PAHs was more similar to that of charcoal abundance. Thus, the HMW PAHs were mainly formed from a local fire near the study area, while the LMW PAHs were likely originated from remote locations. Our results suggest that the concentration and composition of PAHs are useful to understand the frequency of fire activity in both local and remote areas.

## Supporting information

S1 File (DOCX)

S2 File Dataset concentration of diagenetic PAHs with depth of Hole 8, Tinbarap peat core. (ZIP)

S3 File Dataset concentration of pyrogenic PAHs with depth of Hole 8, Tinbarap peat core. (ZIP)

S4 File Dataset concentration of alkylated PAHs with depth of Hole 8, Tinbarap peat core. (ZIP)
