# Supplementary material for: The significance of pyrogenic polycyclic aromatic hydrocarbons in Borneo peat core for the reconstruction of fire history
Source: PLoS One. 2021 Sep 8;16(9):e0256853. doi: 10.1371/journal.pone.0256853 (PMC8425563; doi:10.1371/journal.pone.0256853)

**Supporting information**

**S1 Appendix**

| 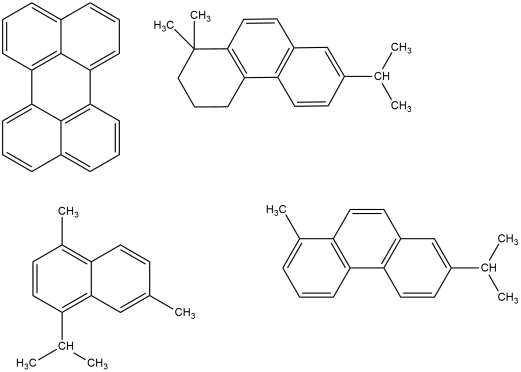  Perylene (Per) | 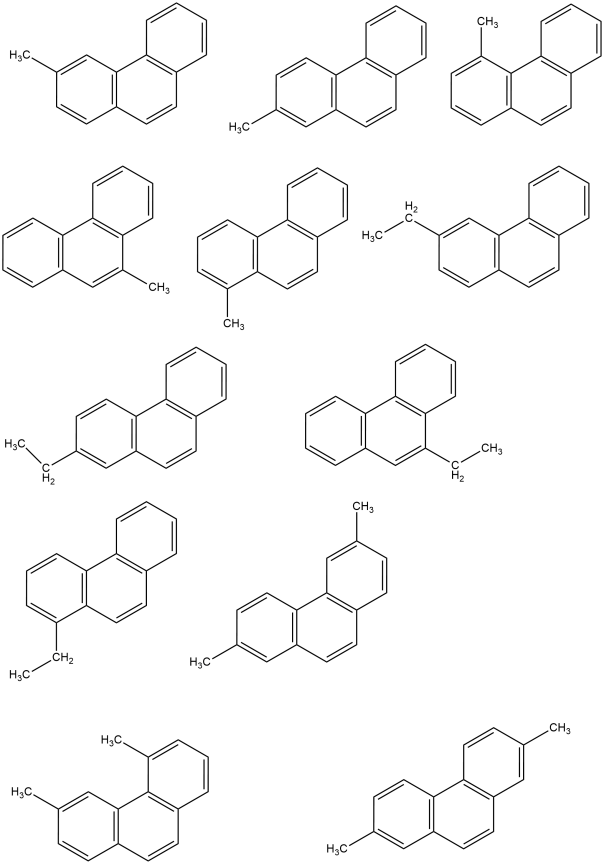  3-Methylphenanthrene (3-MP) | 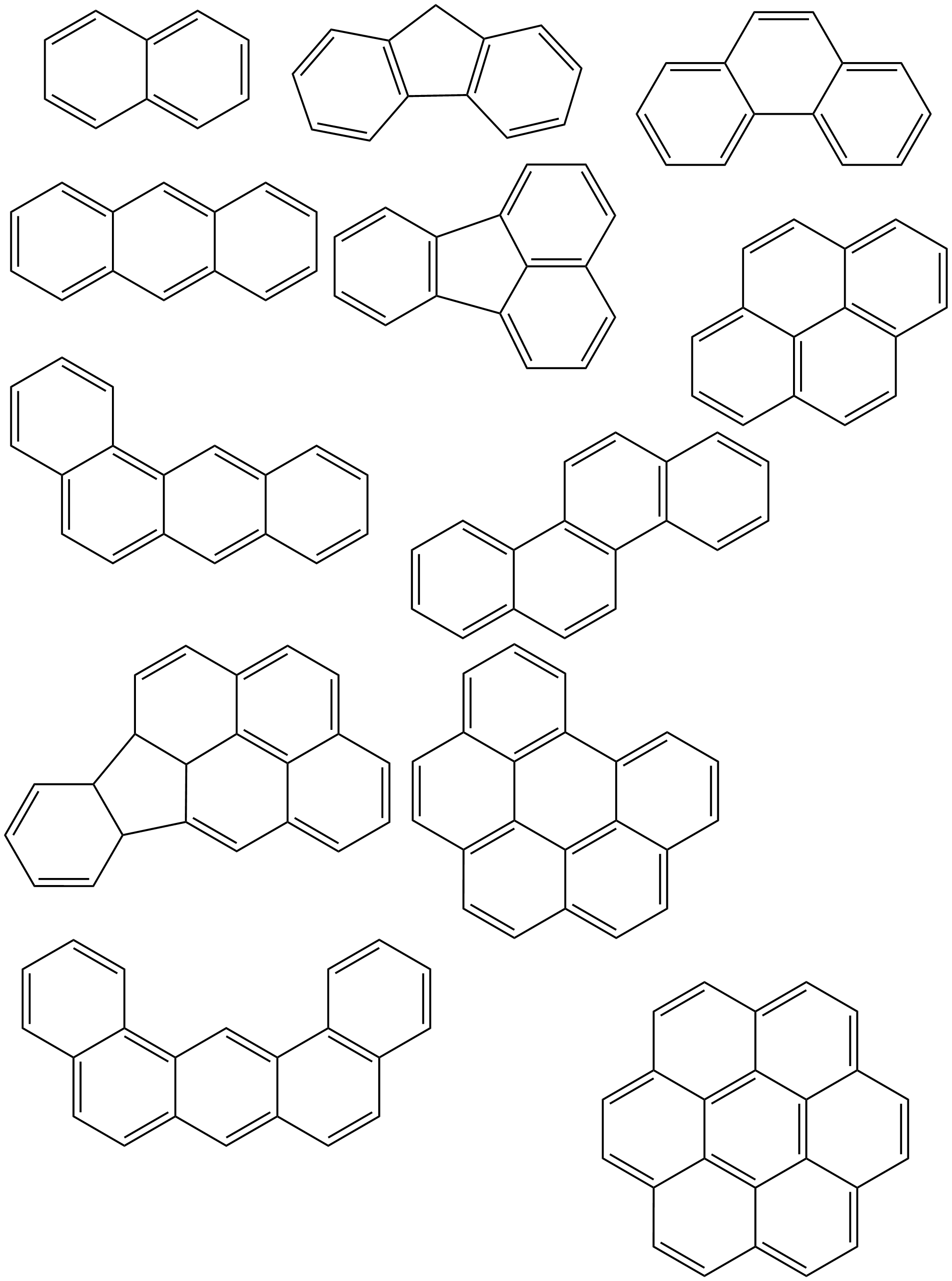  Naphthalene (Naph) |
| --- | --- | --- |
| 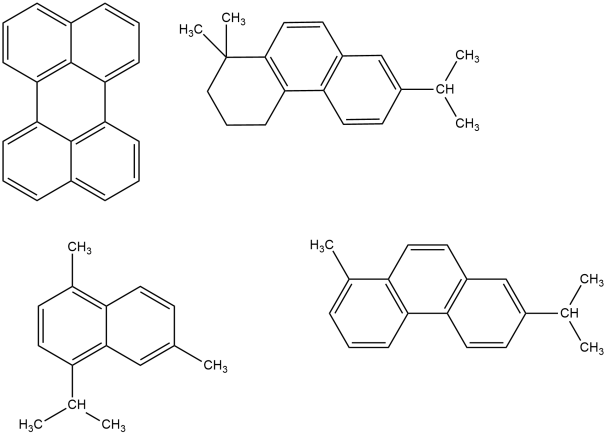  Retene (Ret) | 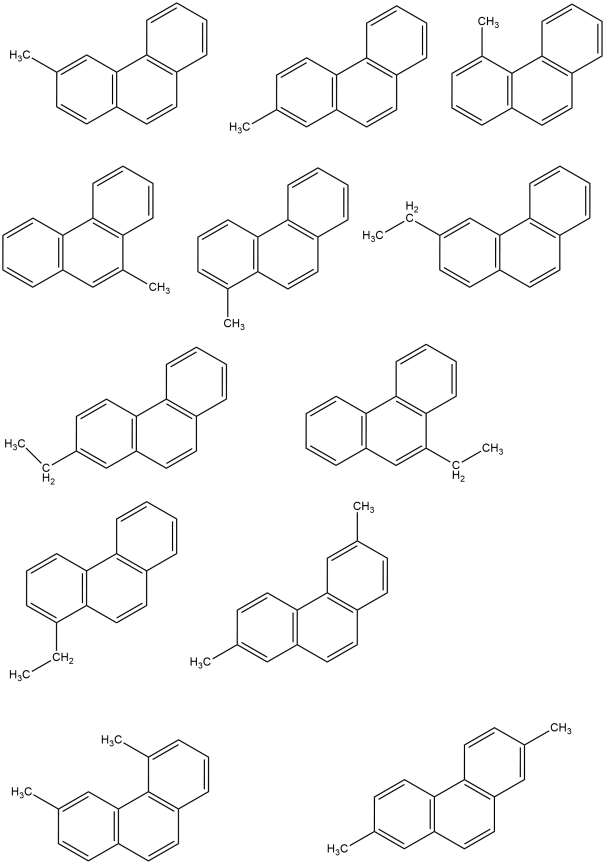  2-Methylphenanthrene (2-MP) | 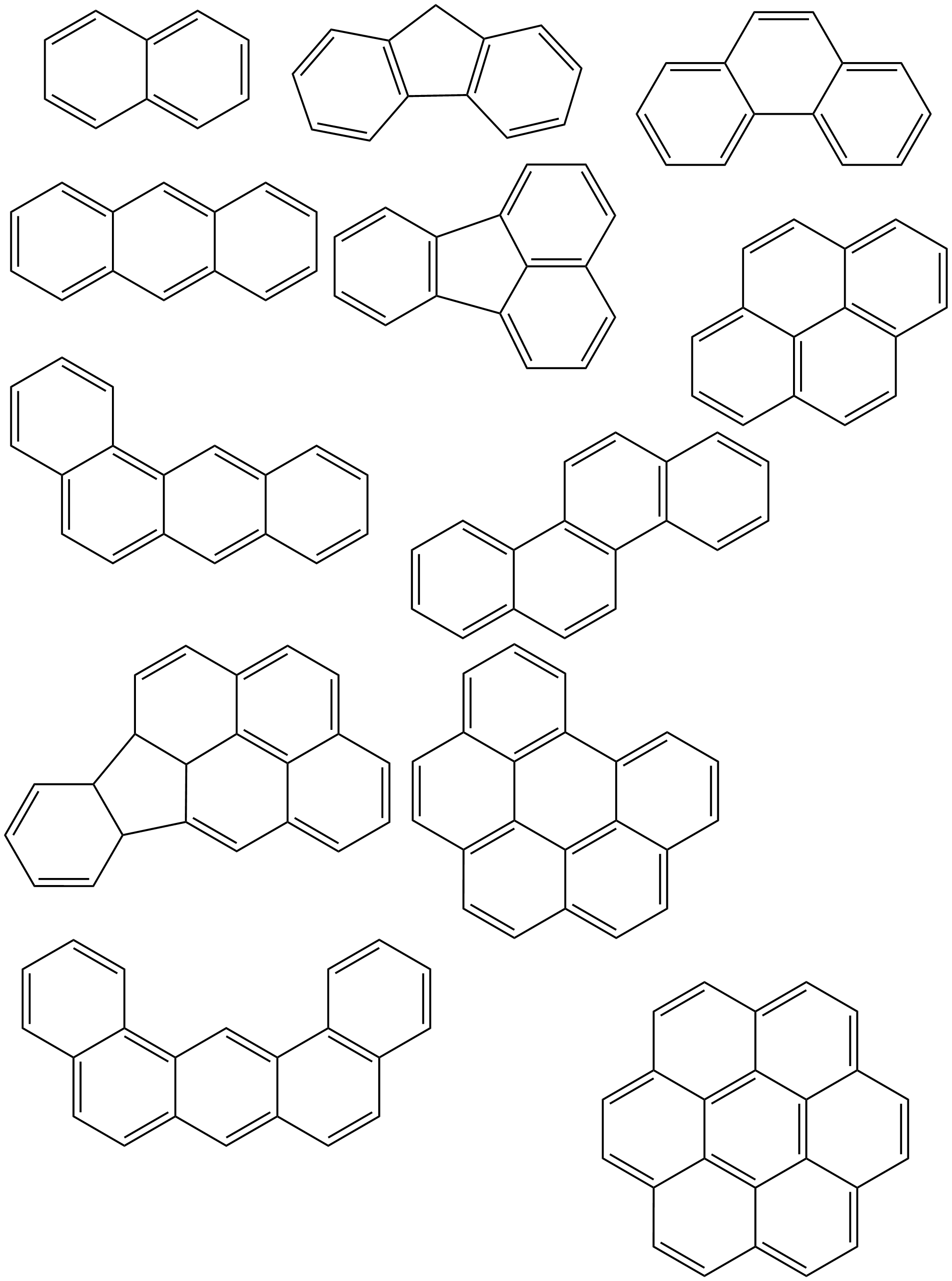  Fluorene (Flu) |
| 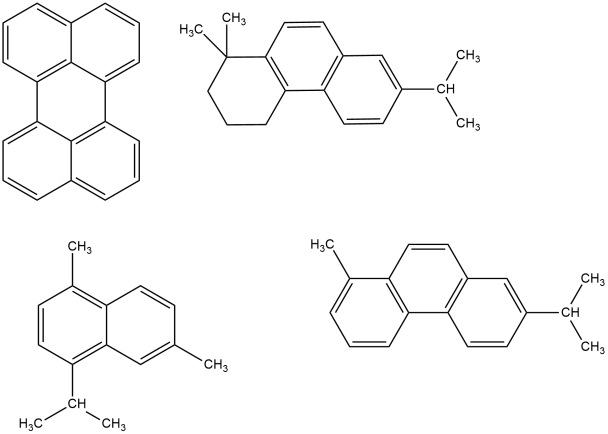  Cadalene (Cad) | 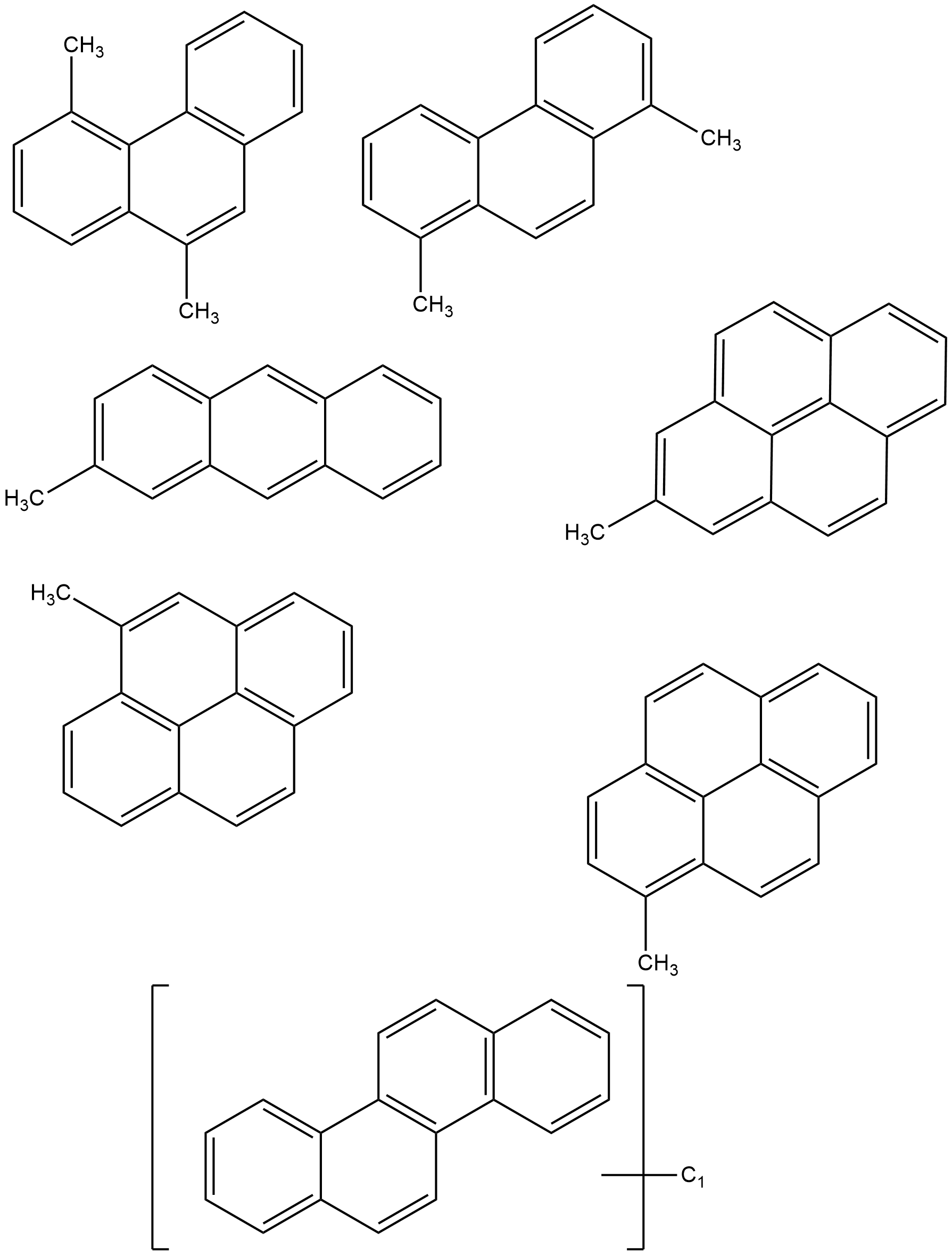  2-Methylanthracene (2-MA) | 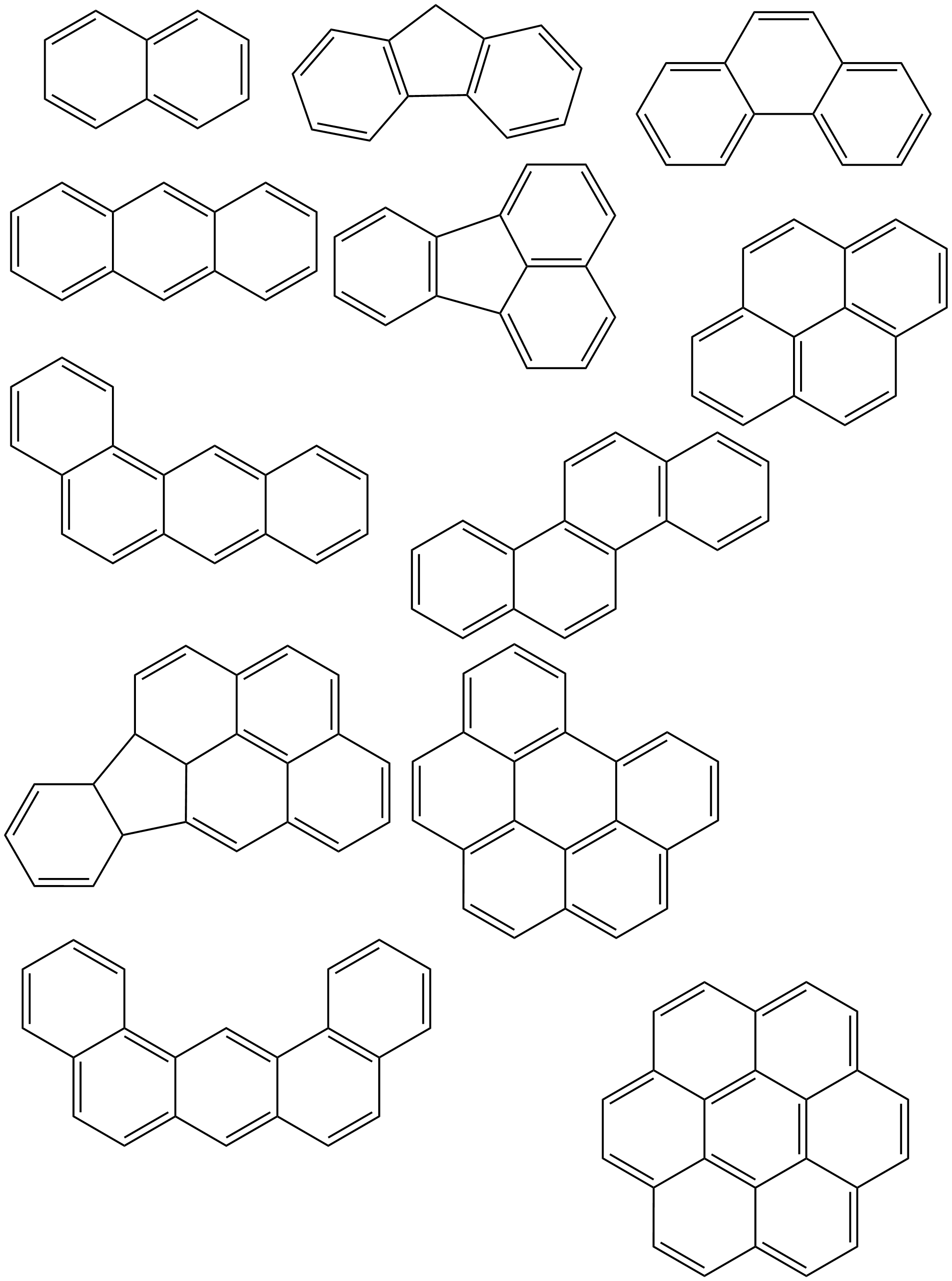  Phenanthrene (Phe) |
| 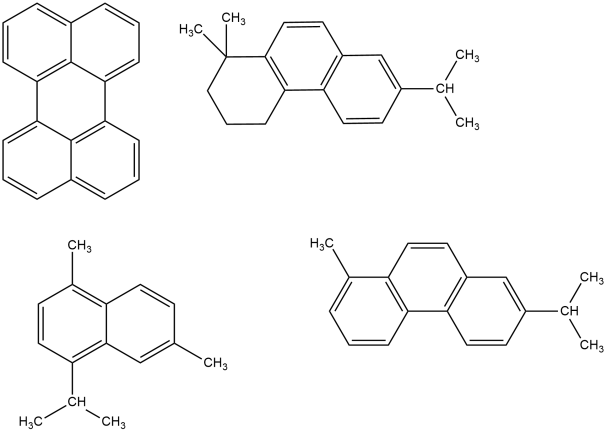Simonellite (Sim) | 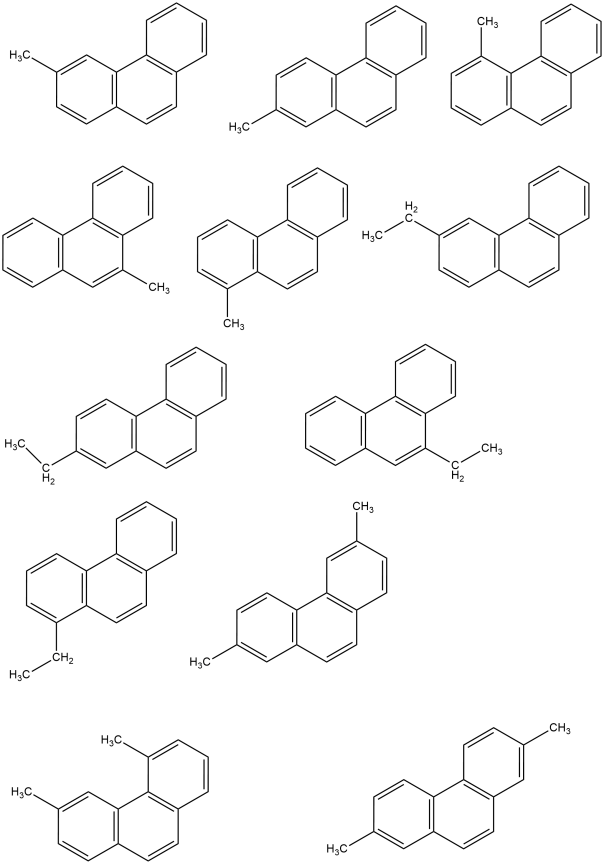  4-Methylphenanthrene (4-MP) | 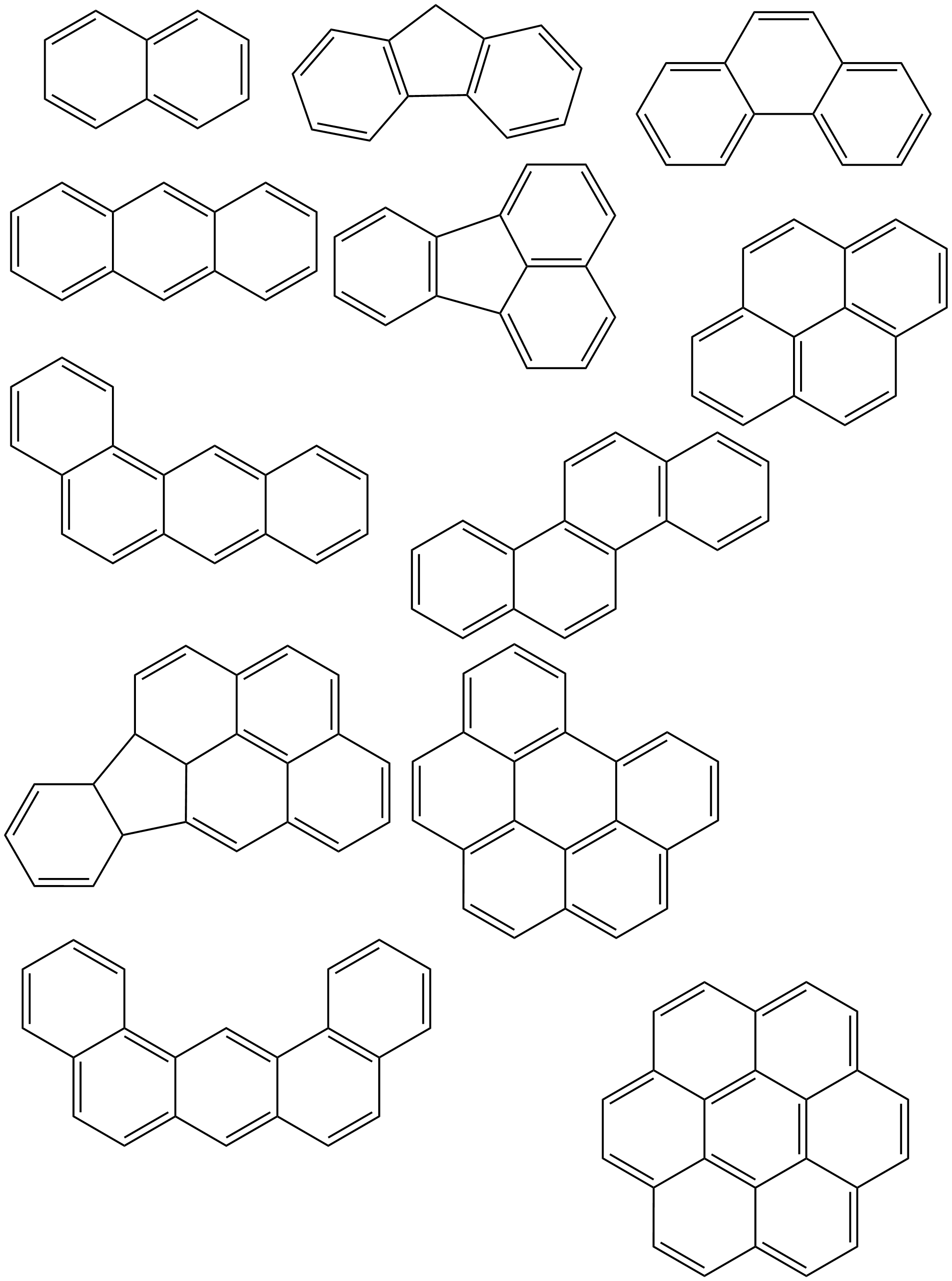  Anthracene (Ant) |
| 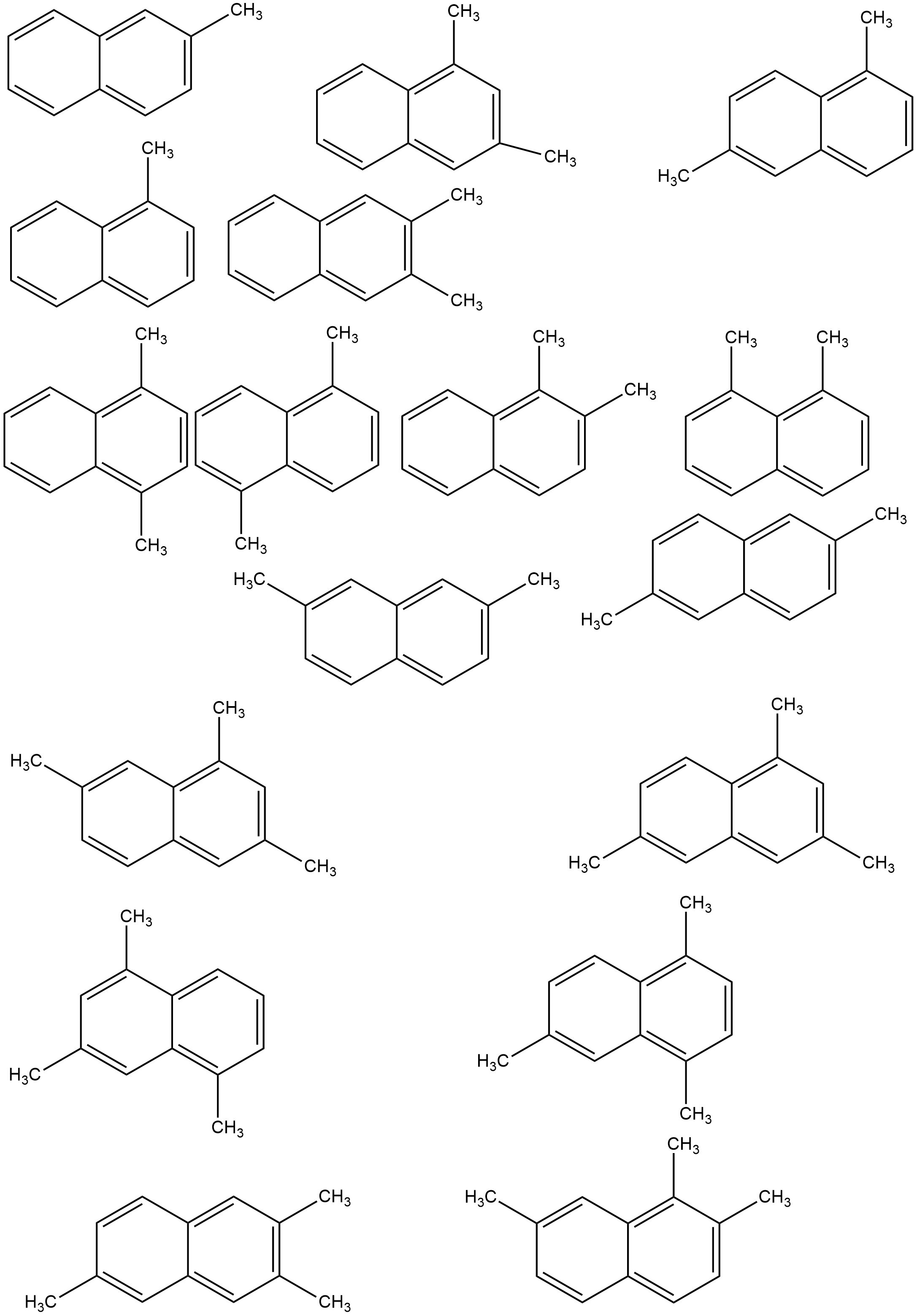  2-Methylnaphthalene (2-MN) | 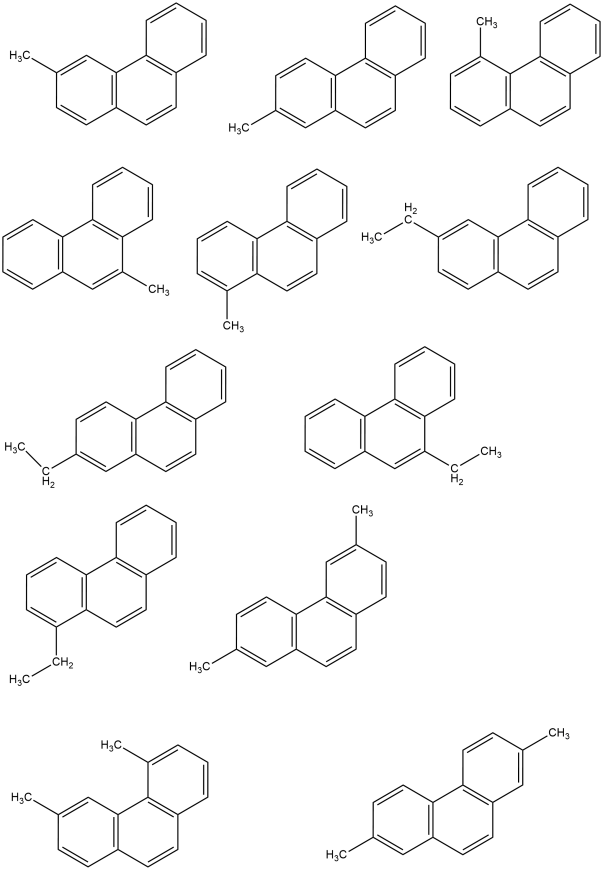  9-Methylphenanthrene (9-MP) | 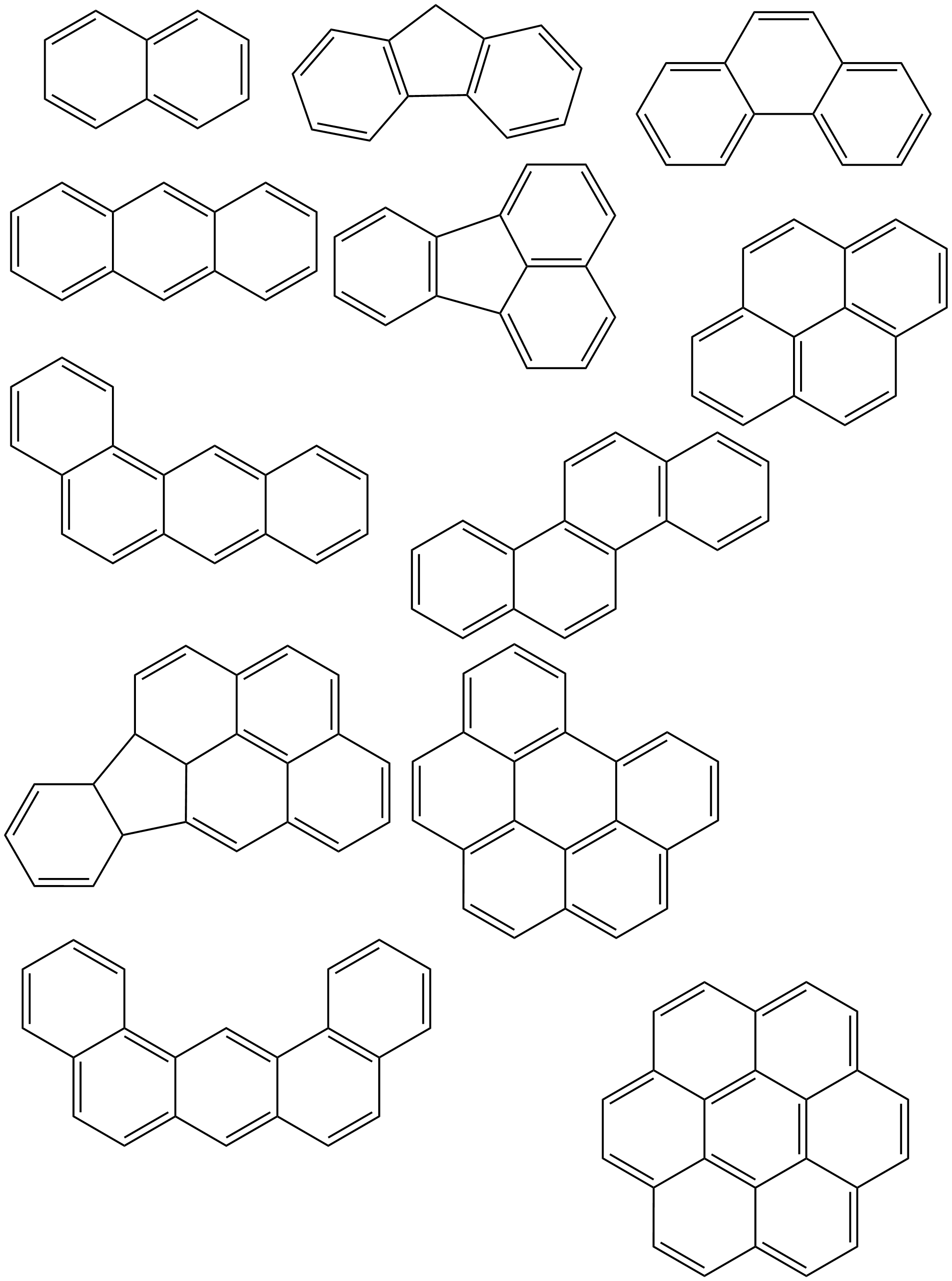  Fluoranthene (Fla) |
| 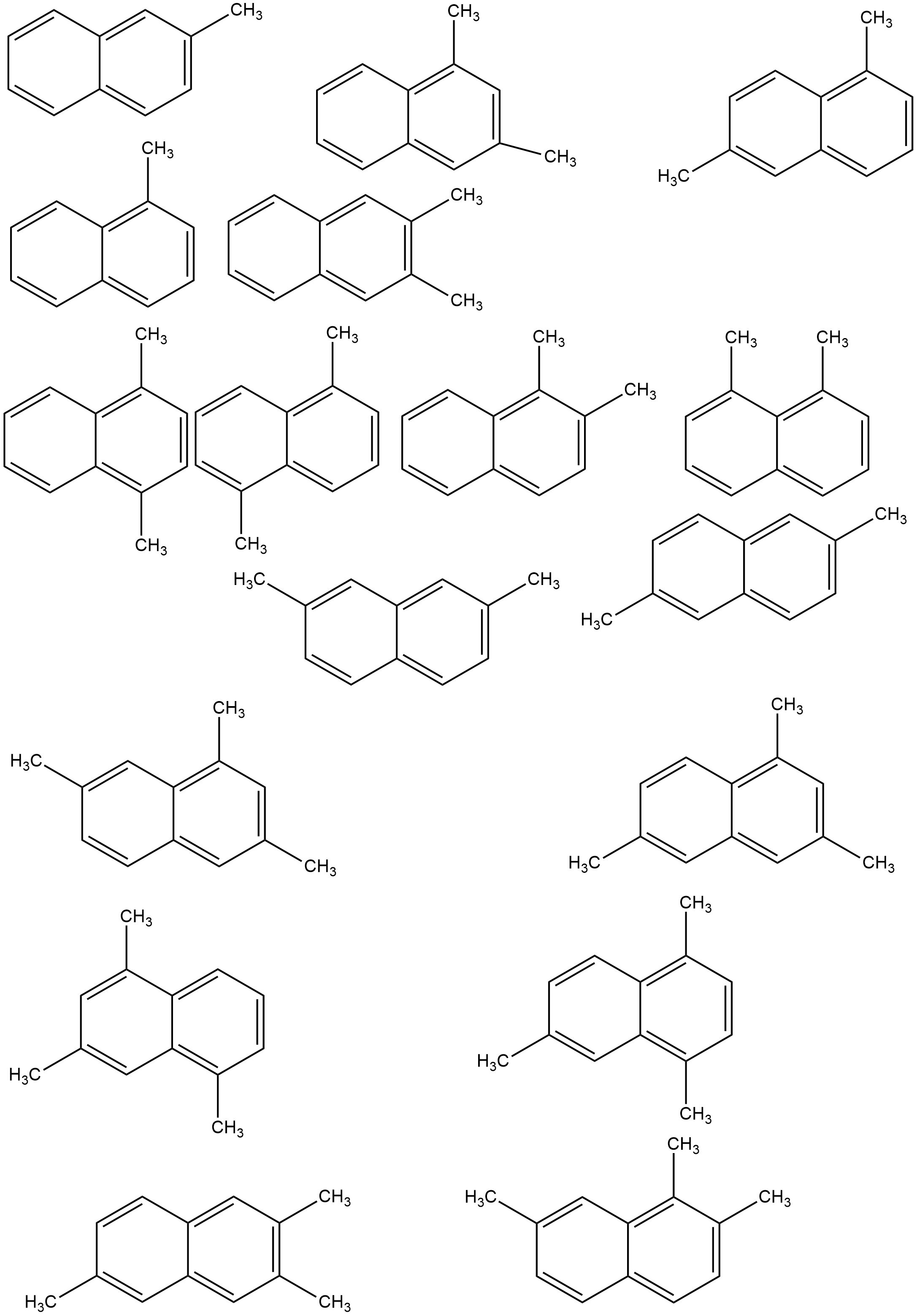  1-Methylnaphthalene (1-MN) | 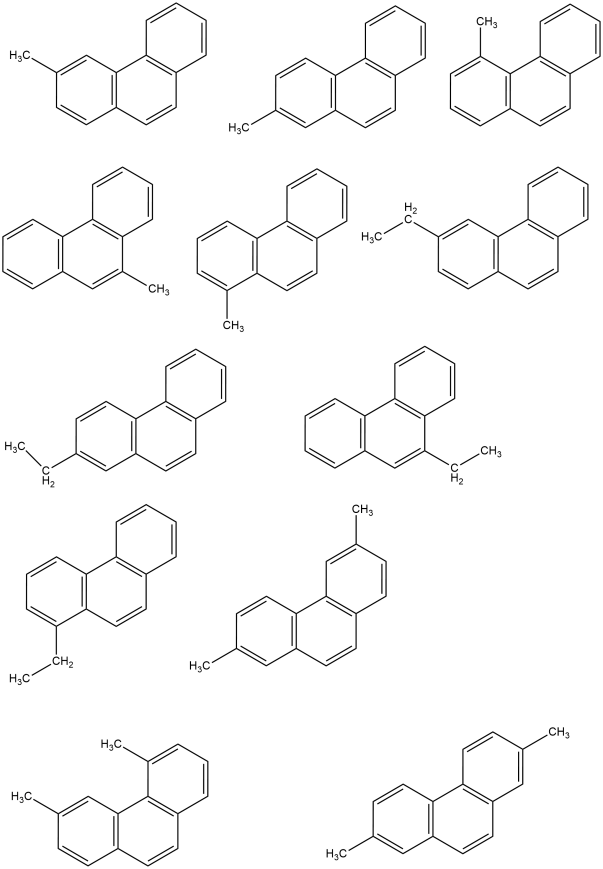  1-Methylphenanthrene (1-MP) | 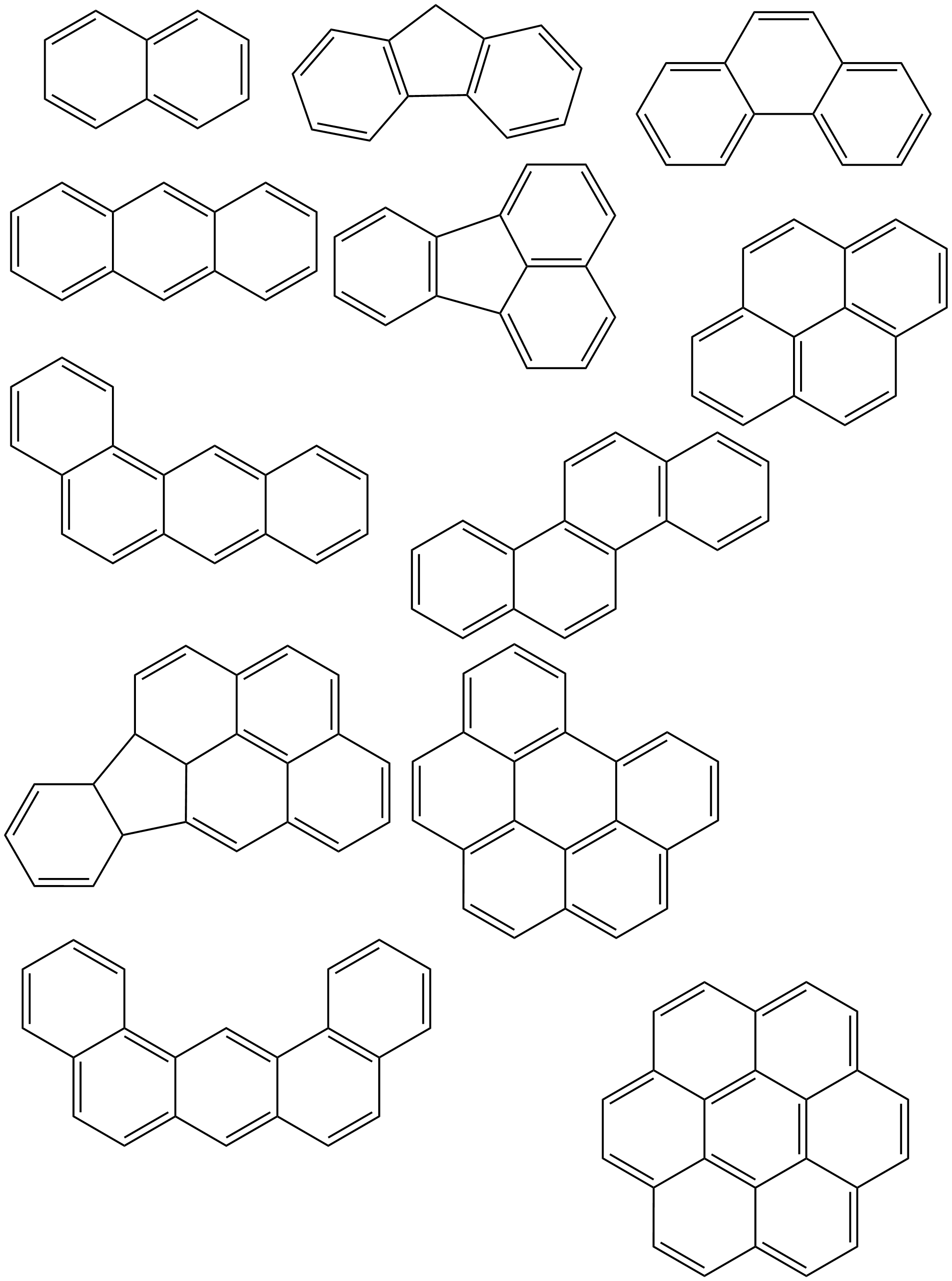  Pyrene (Py) |
| 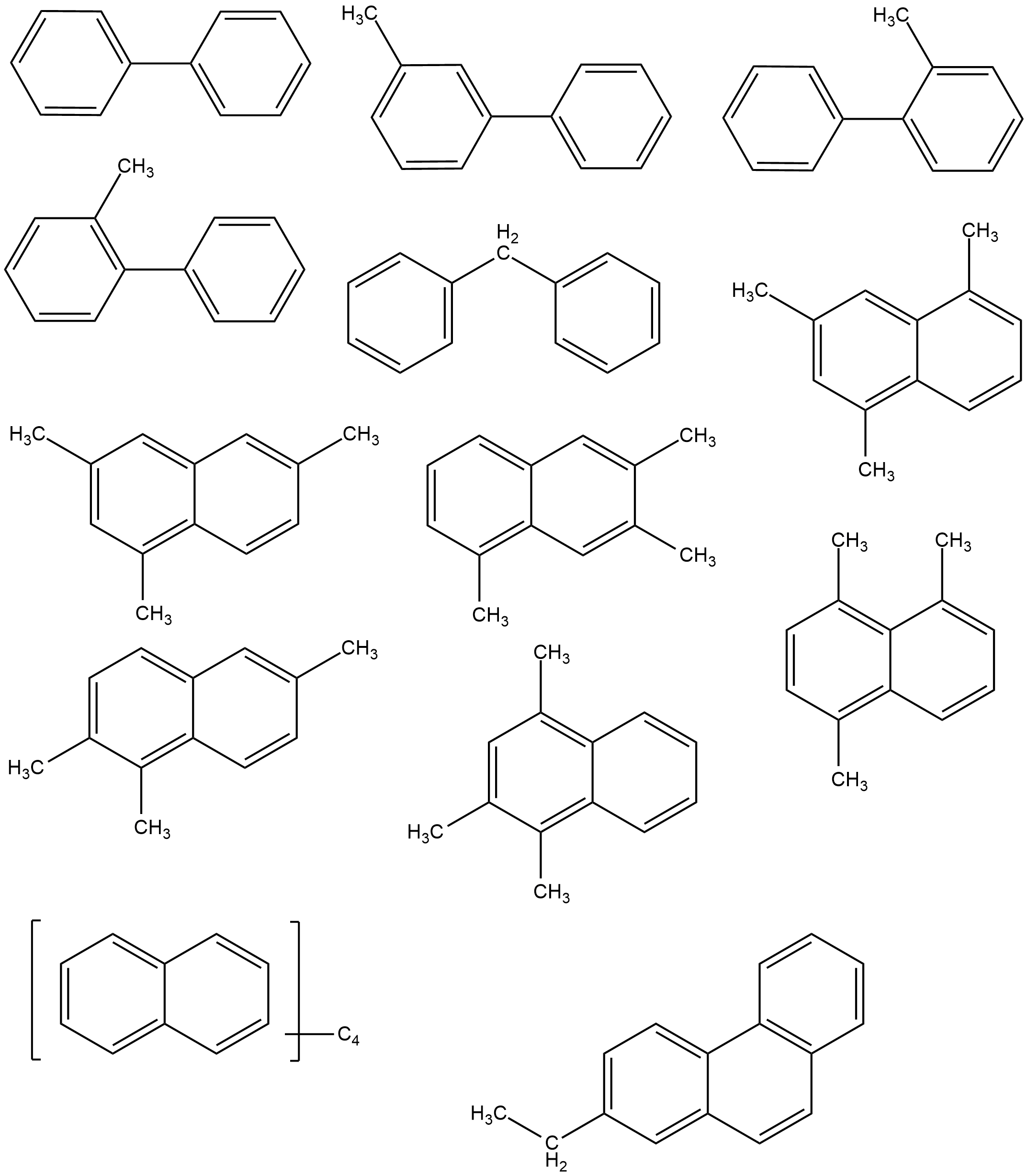  Biphenyl (Bip) | 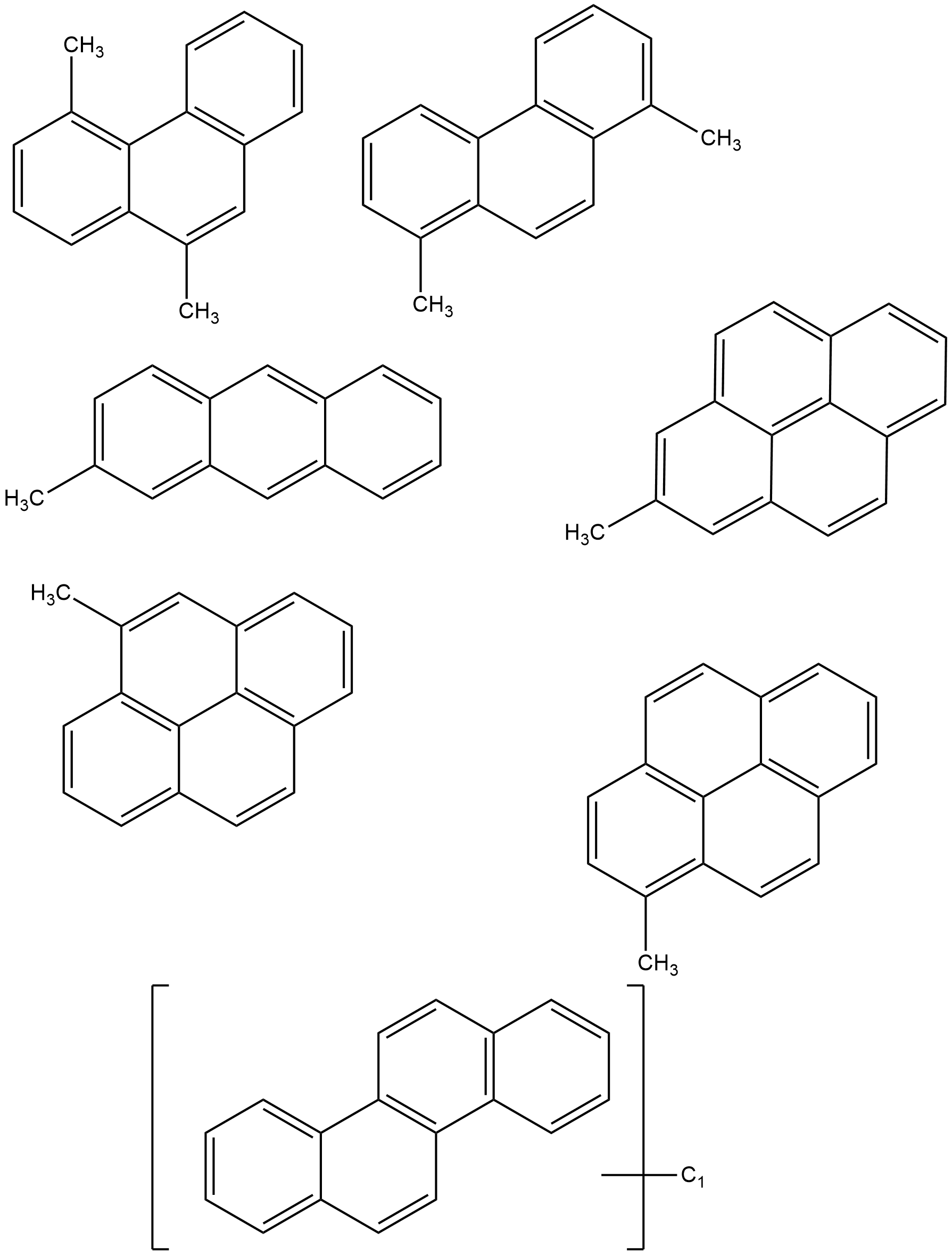  2-Methylpyrene (2-MPy) | 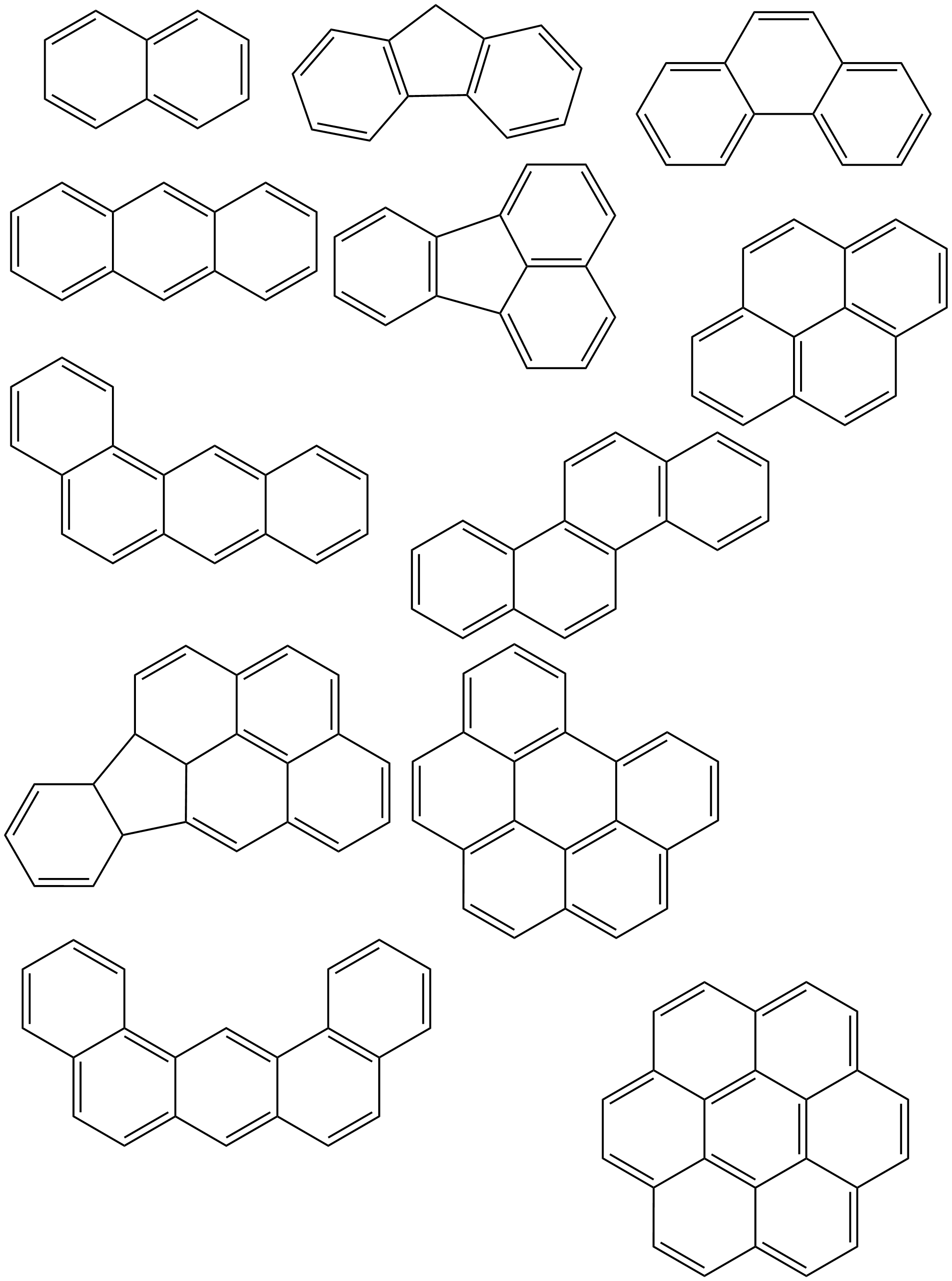Benzo[a]anthracene (BaA) |
| 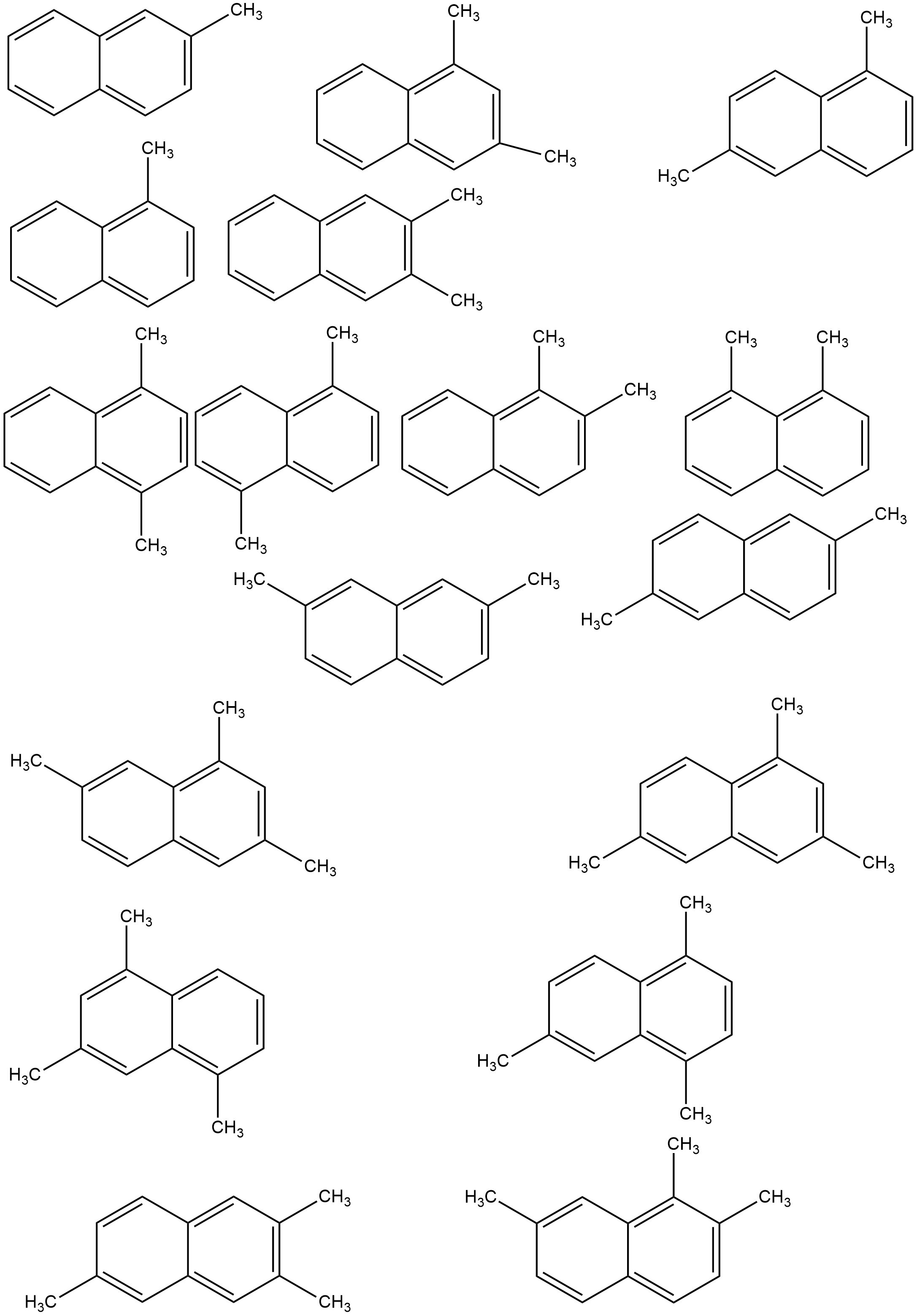  1,3-Dimethylnaphthalene  (1,3-DMN) | 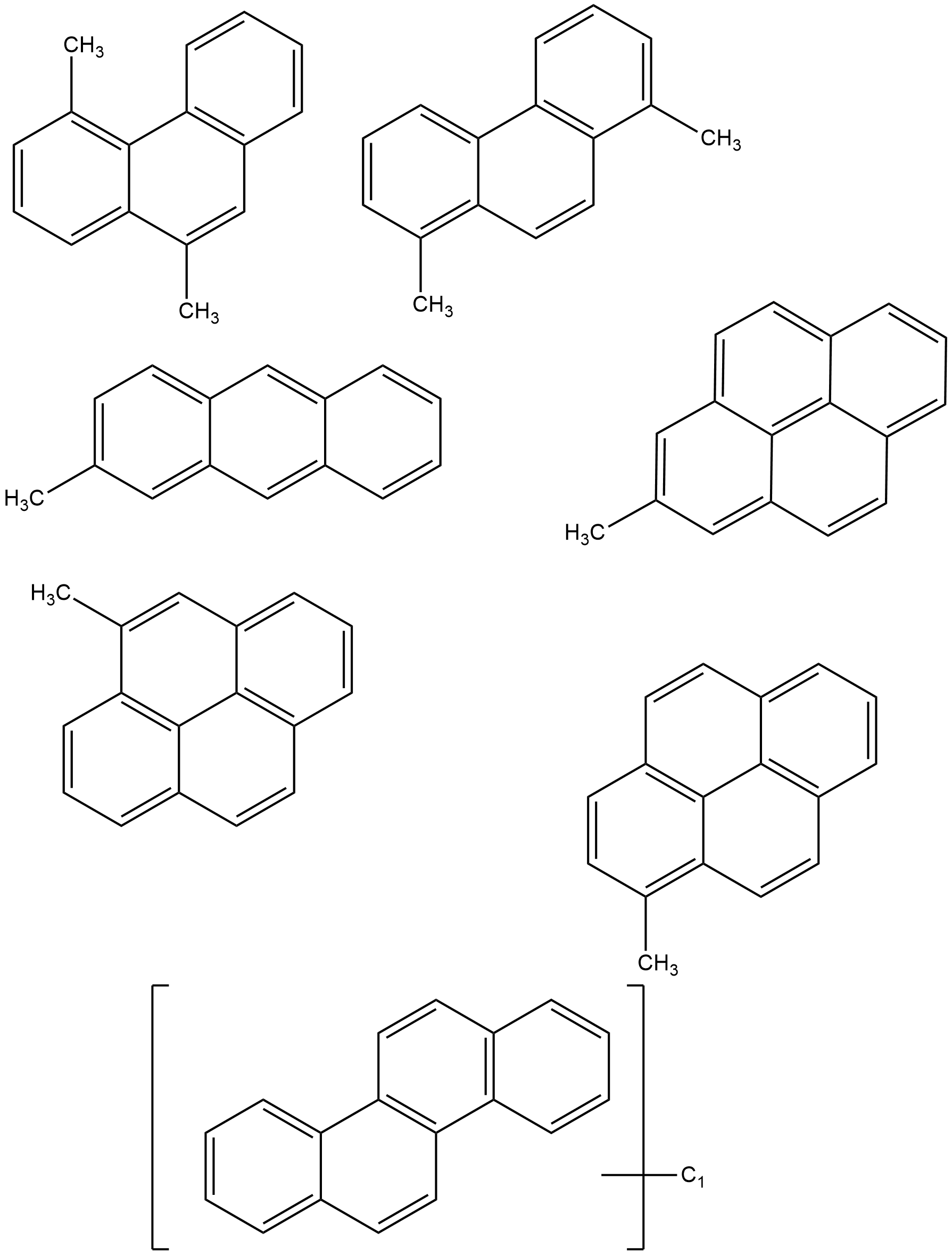  4-Methylpyrene (4-MPy) | 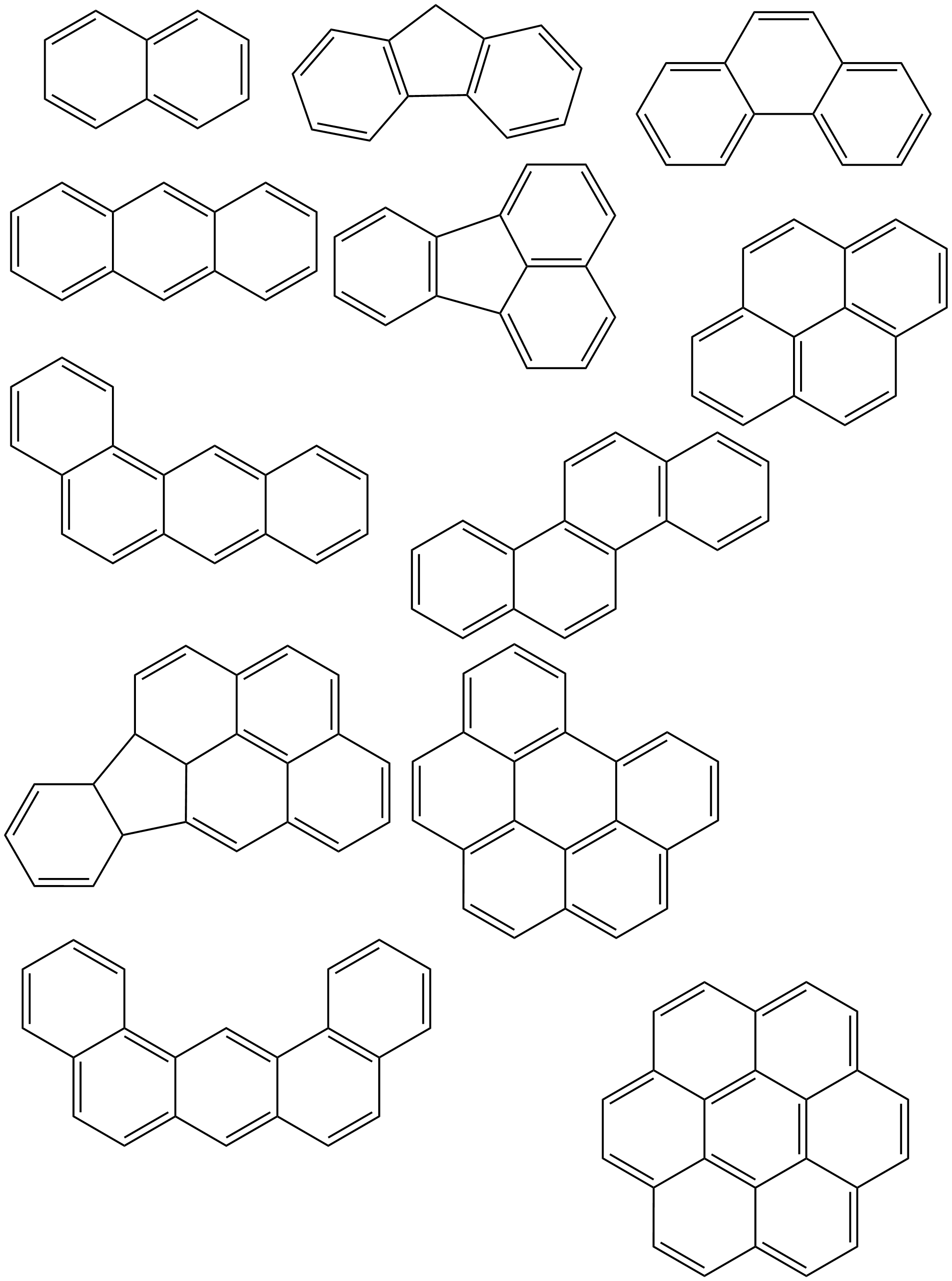  Chrysene (Chr) |
| 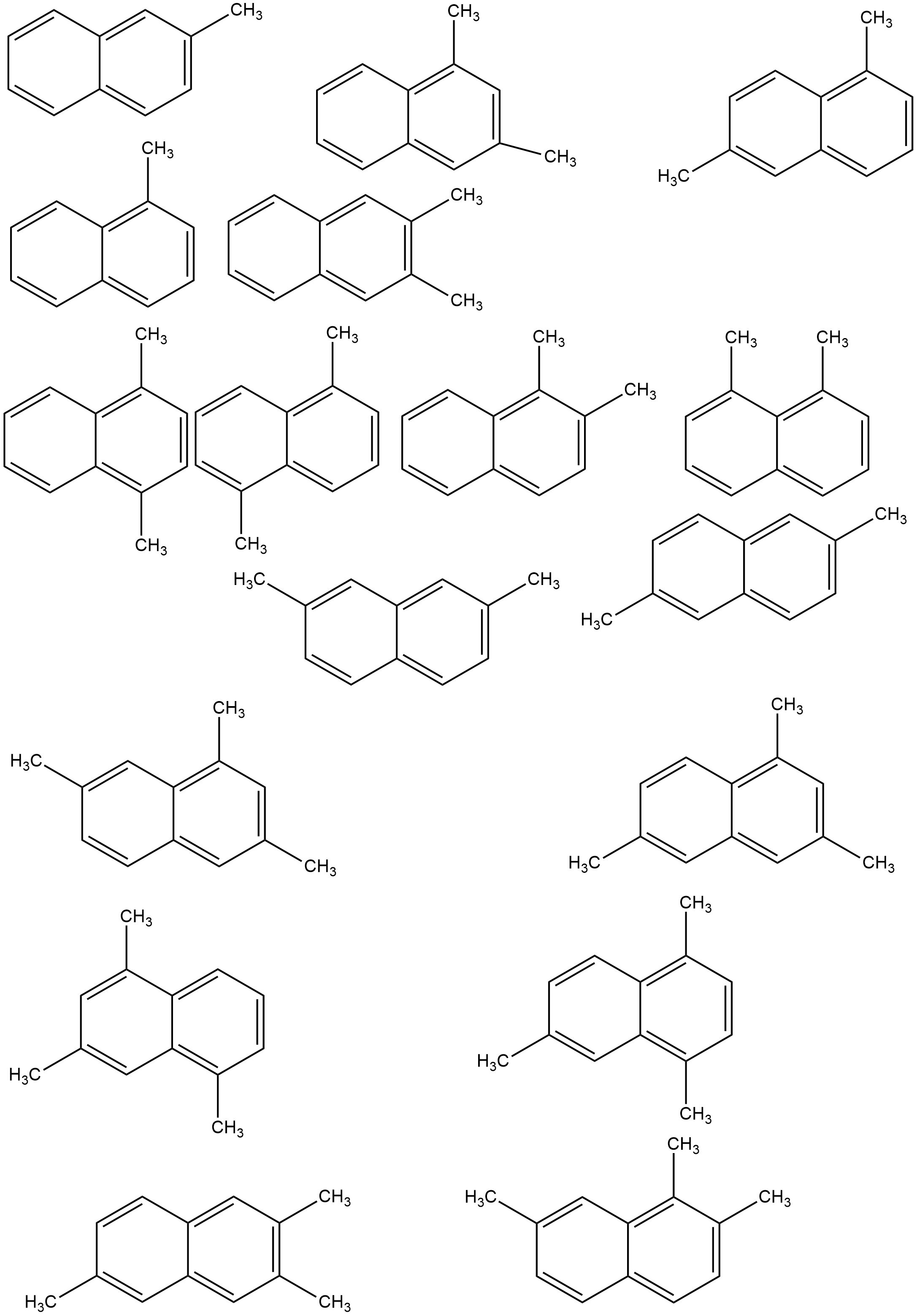  1,6-Dimethylnaphthalene  (1,6-DMN) | 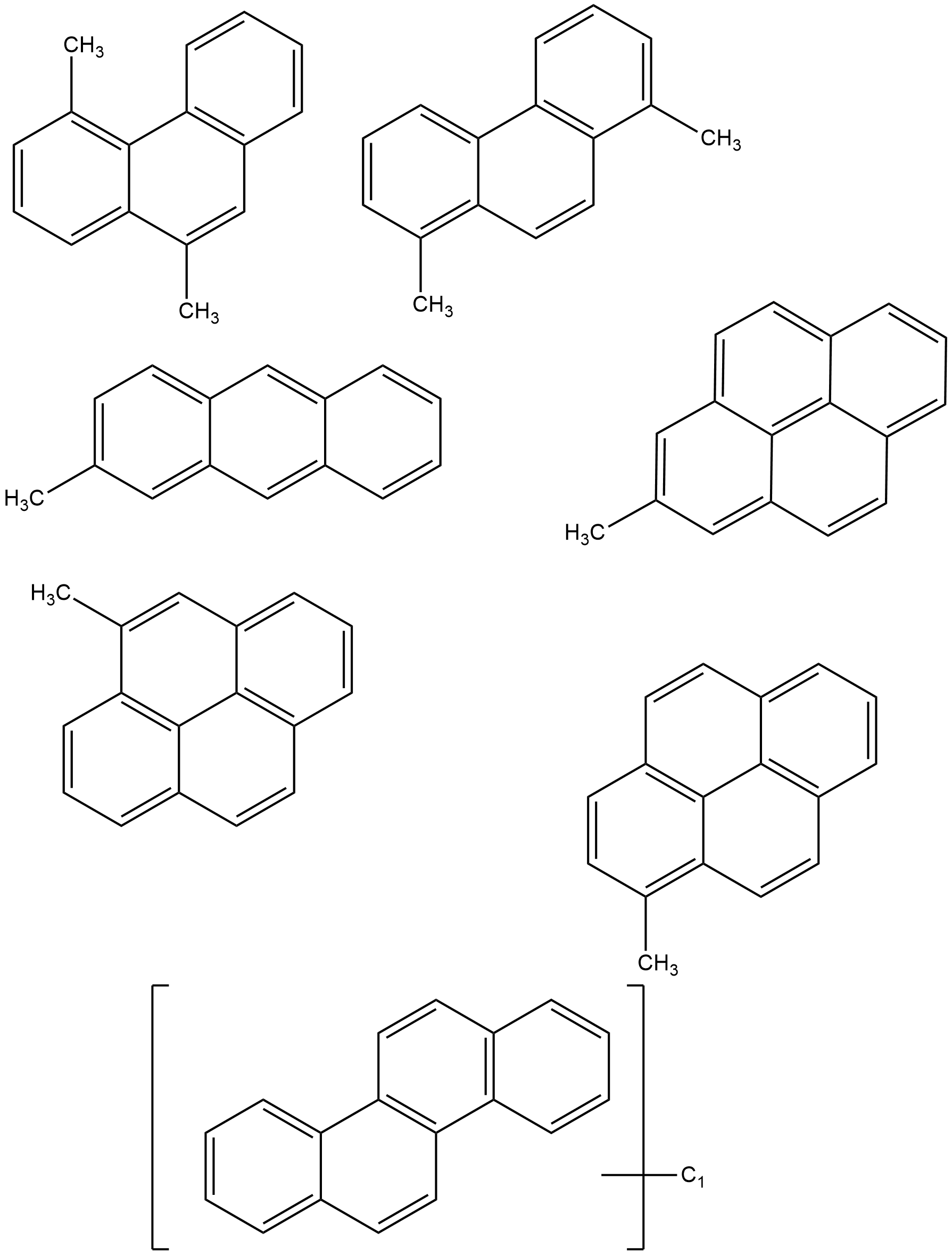  1-Methylpyrene (1-MPy) | 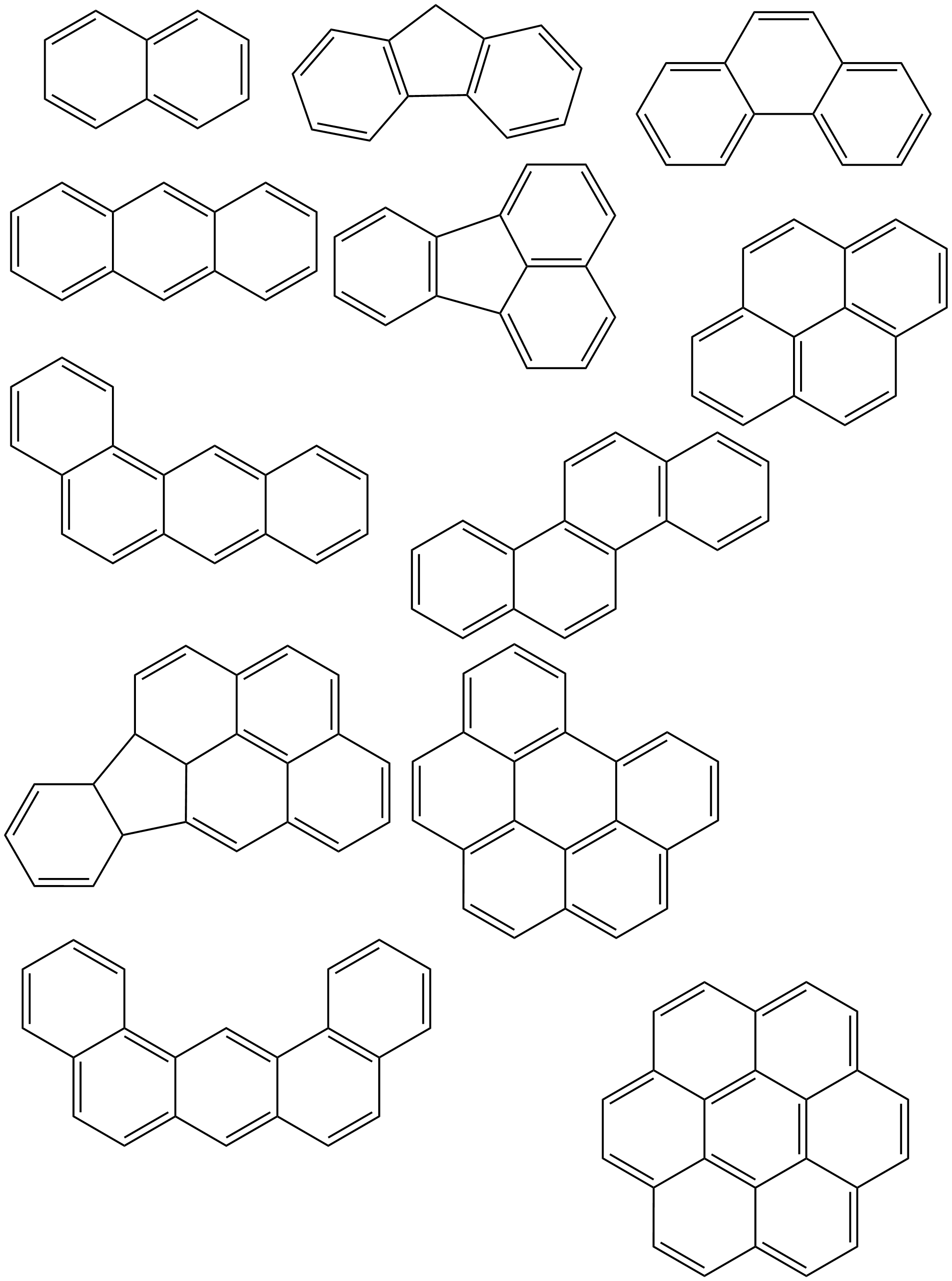Indeno[1,2,3-c,d]pyrene (IPy) |
| 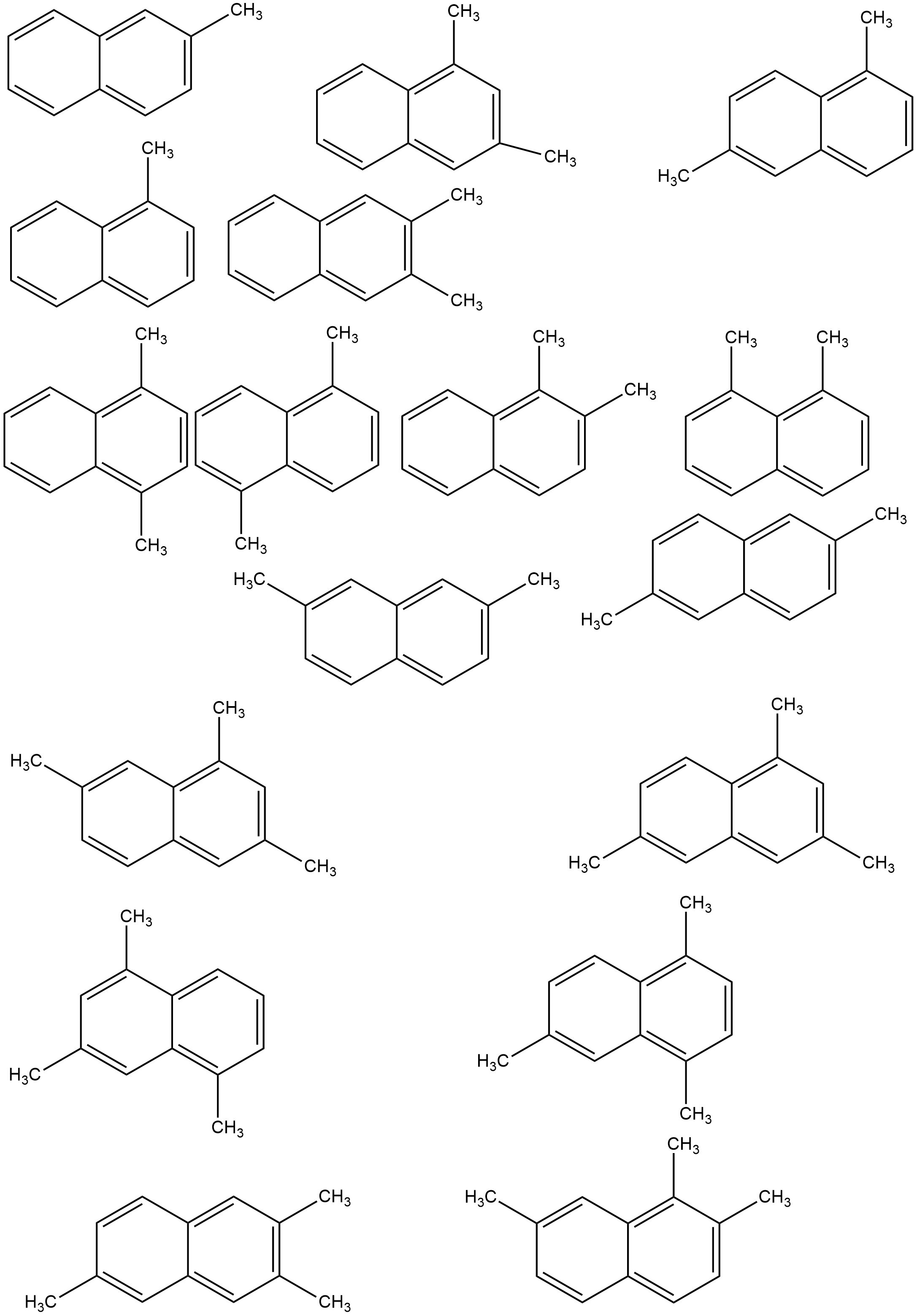  1,4-Dimethylnaphthalene  (1,4-DMN) | 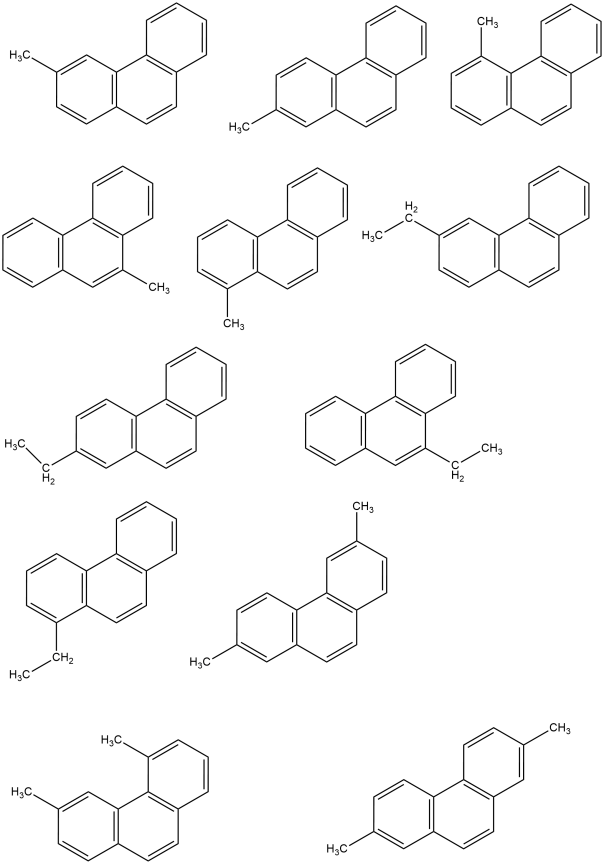  3-Ethylphenanthrene (3-EP) | 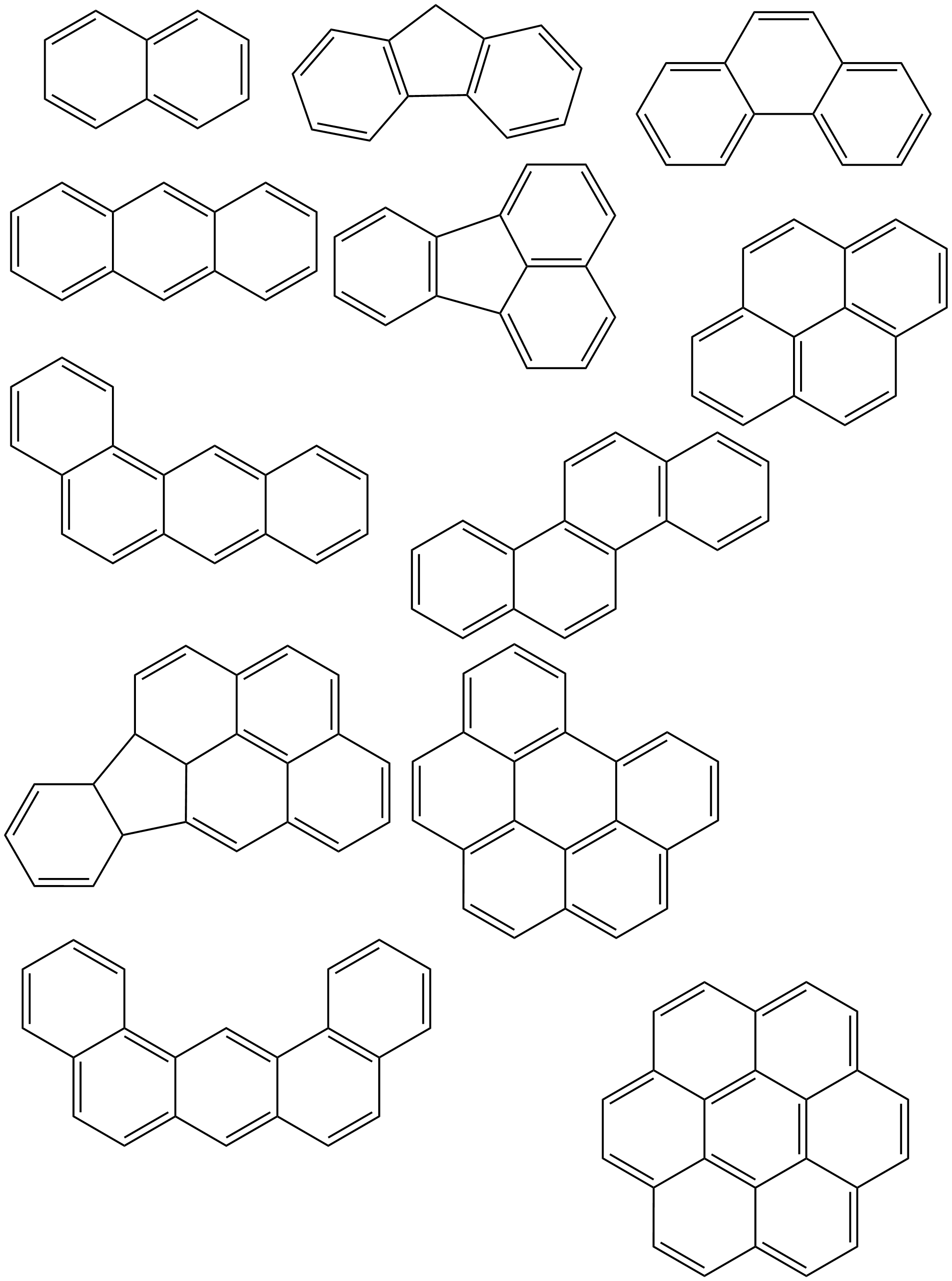Benzo[g,h,i]perylene (BPer) |
| 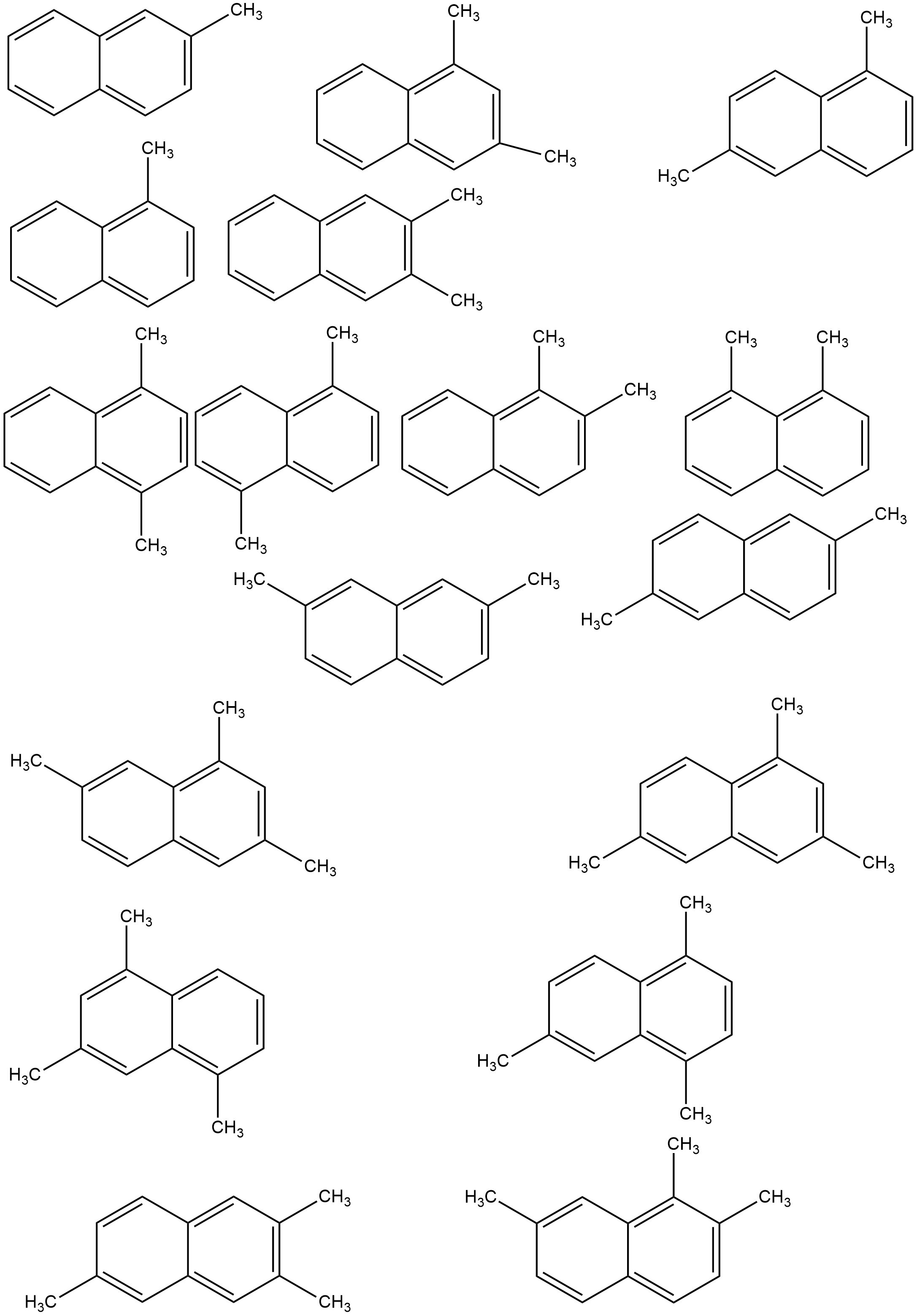  2,3-Dimethylnaphthalene  (2,3-DMN) | 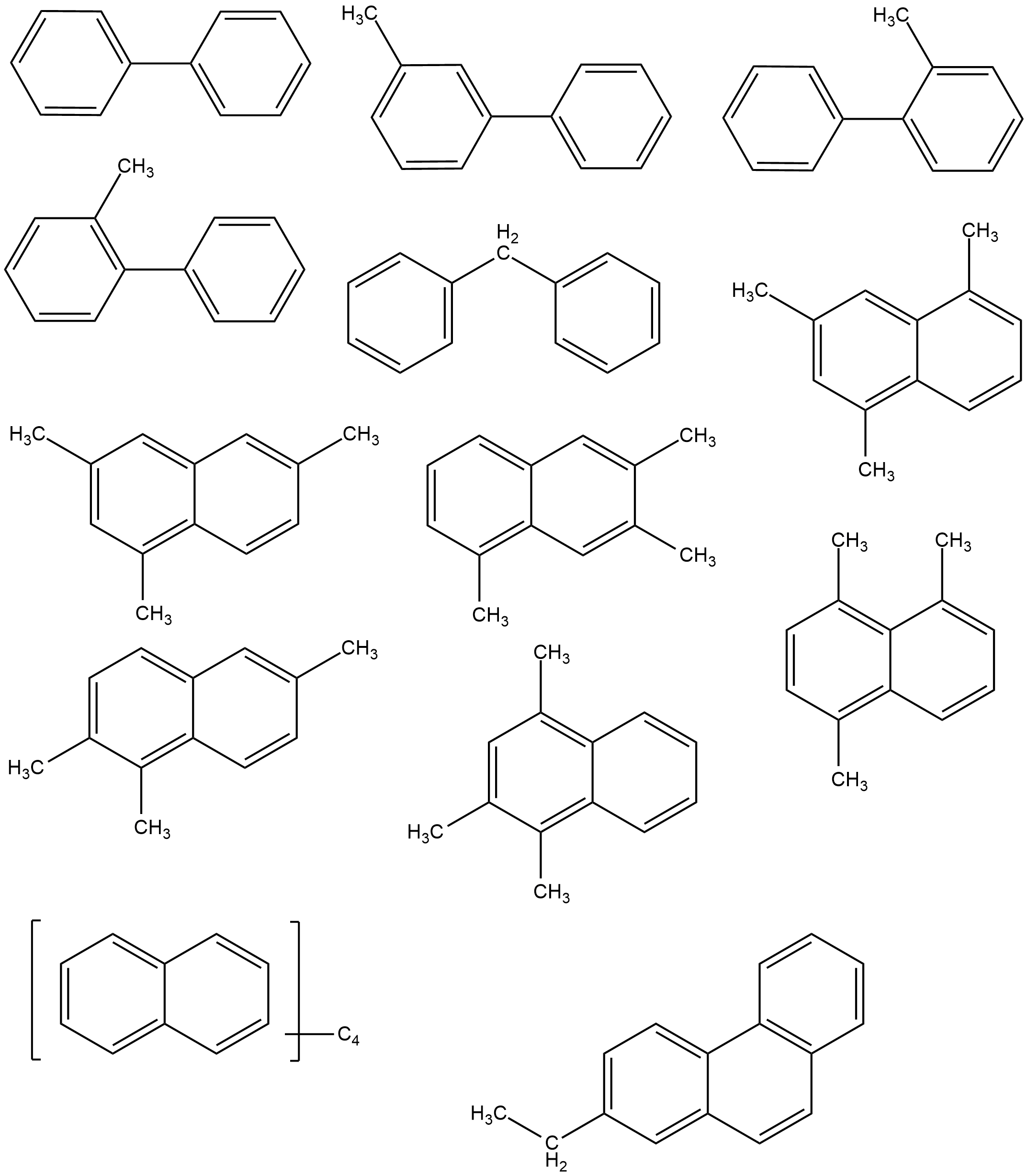  2-Ethylphenanthrene (2-EP) | 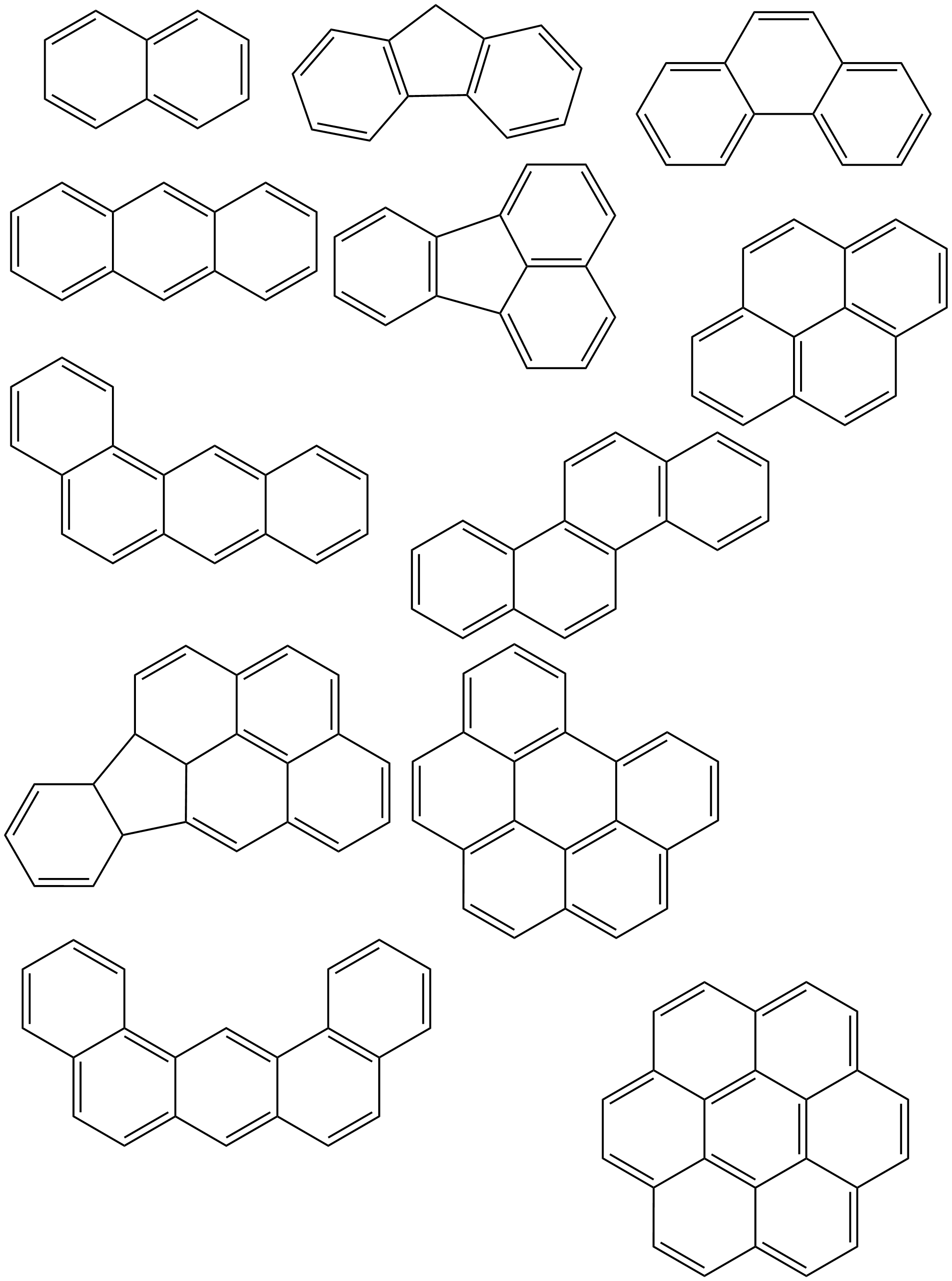Dibenzo[a,h]anthracene (DiA) |
| 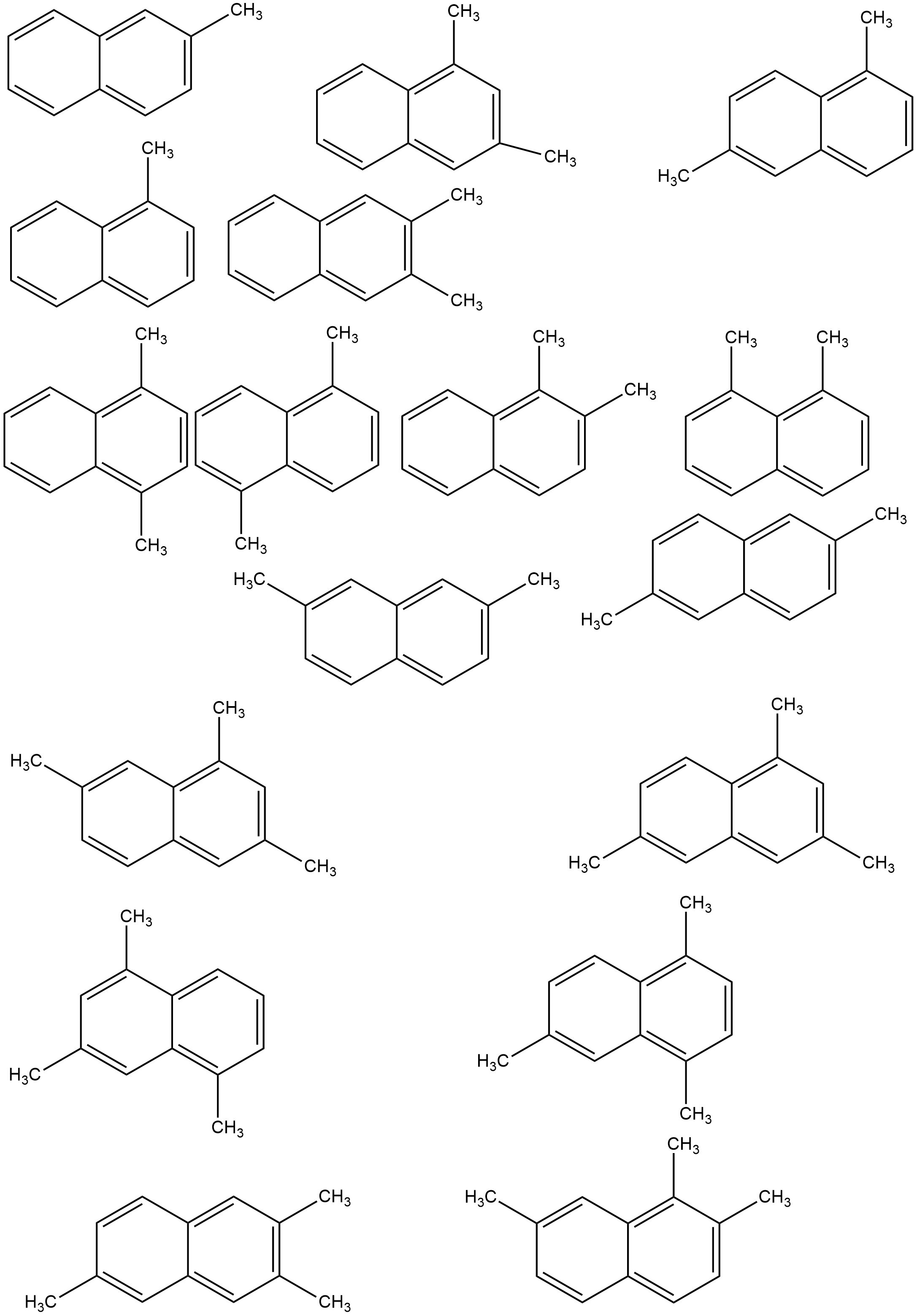  1,5-Dimethylnaphthalene  (1,5-DMN) | 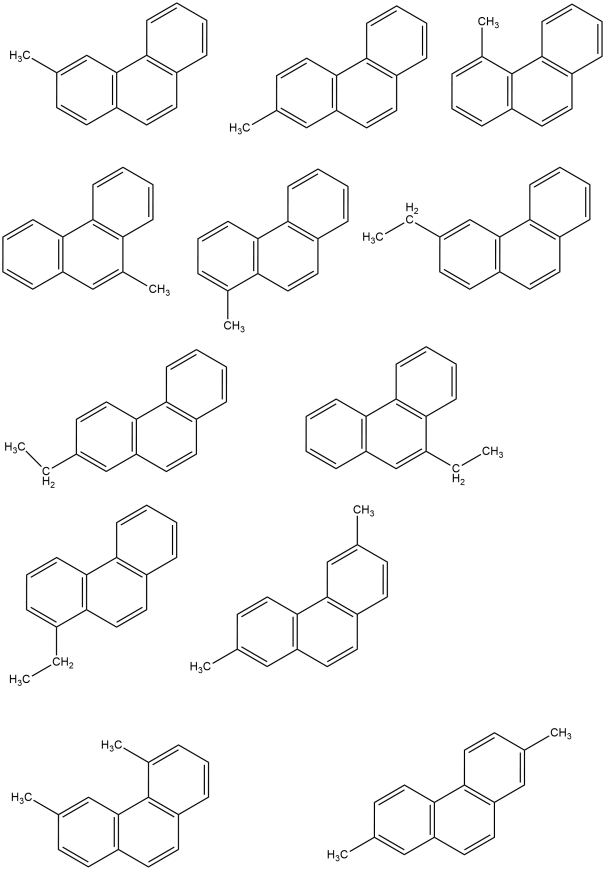  9-Ethylphenanthrene (9-EP) | 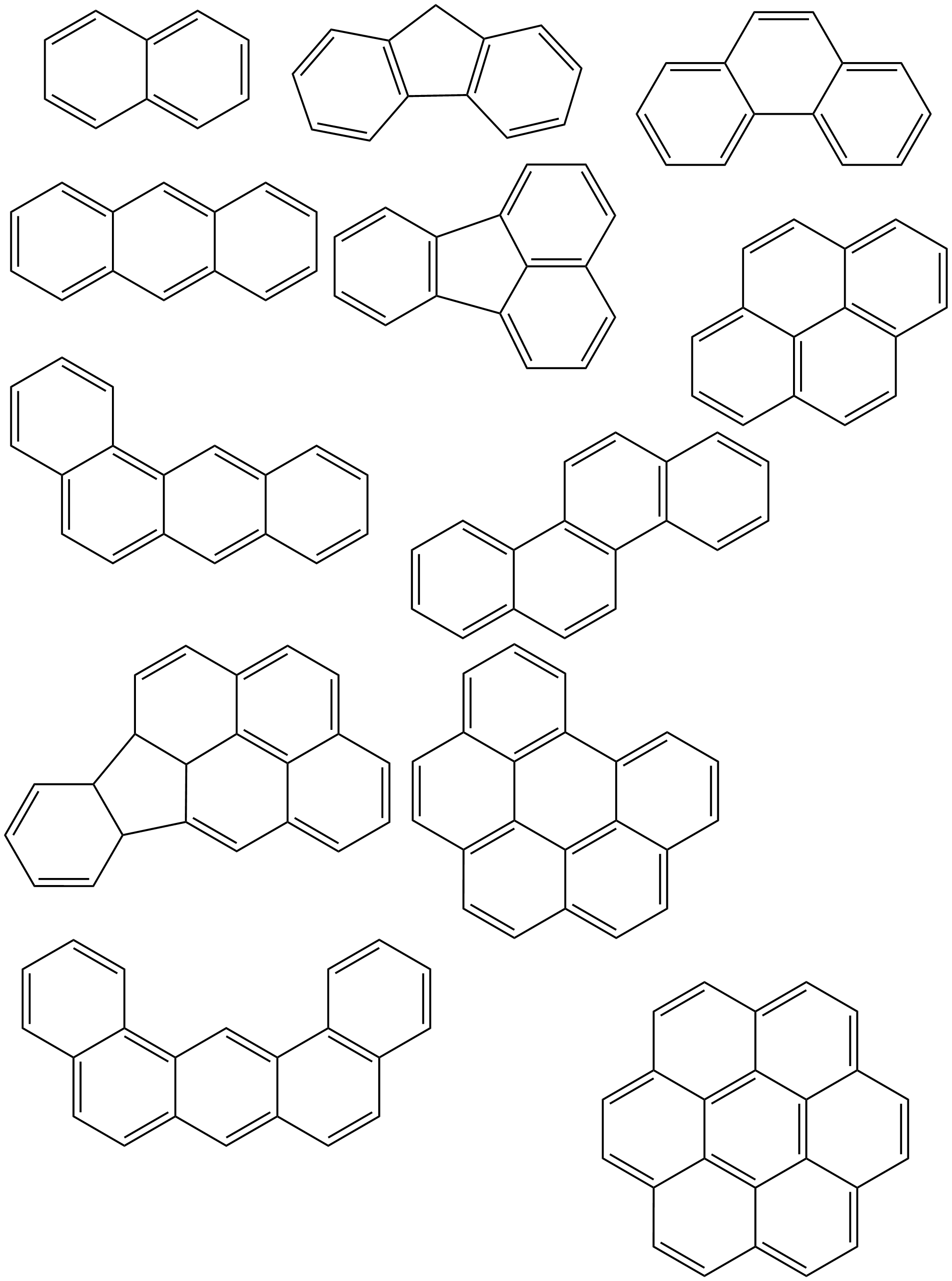  Coronene (Cor) |
| 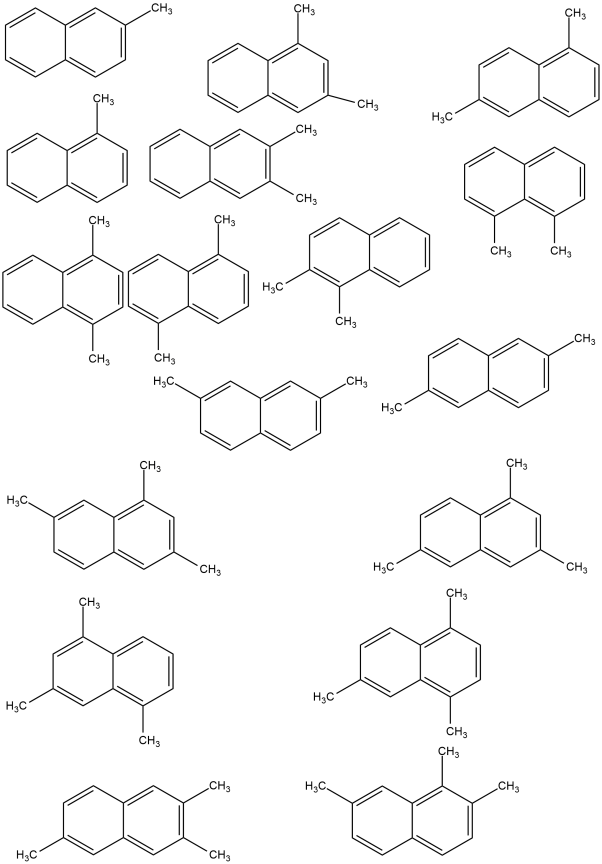  1,2-Dimethylnaphthalene  (1,2-DMN) | 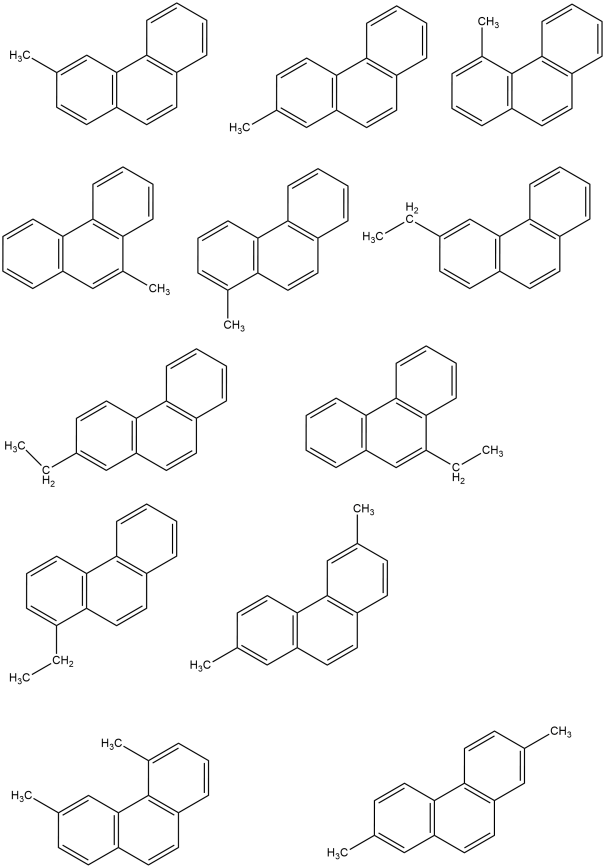  1-Ethylphenanthrene (1-EP) |  |
| 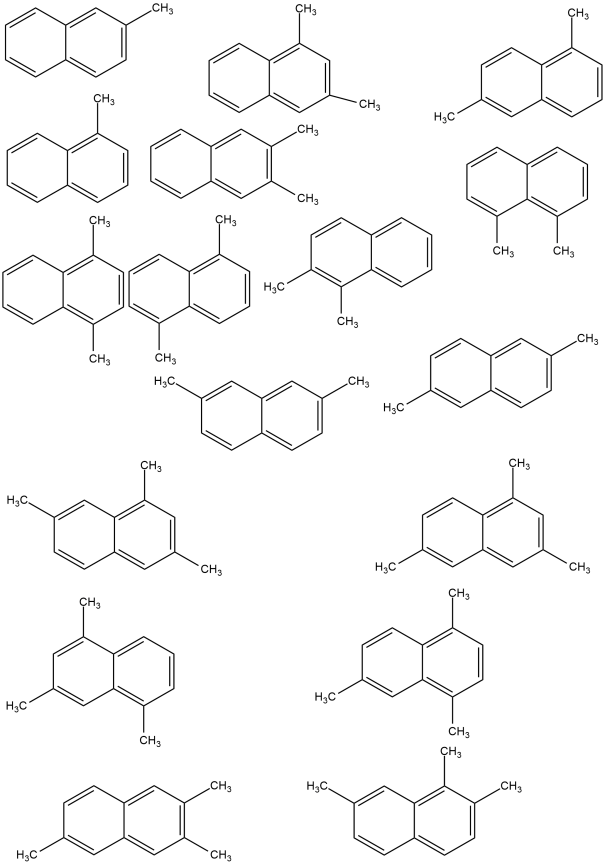  1,8-Dimethylnaphthalene  (1,8-DMN) | 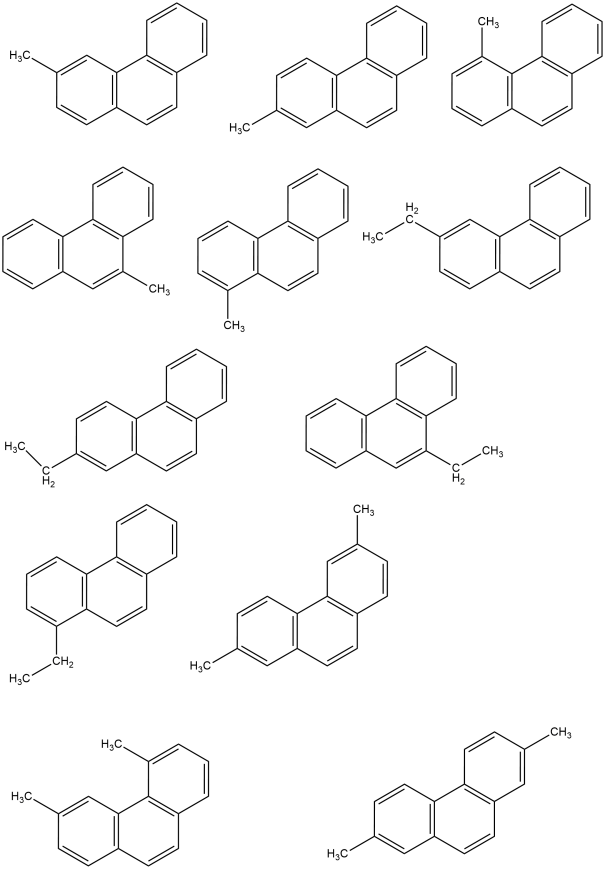  2,6-Dimethylphenanthrene (2,6-DMP) |  |
| 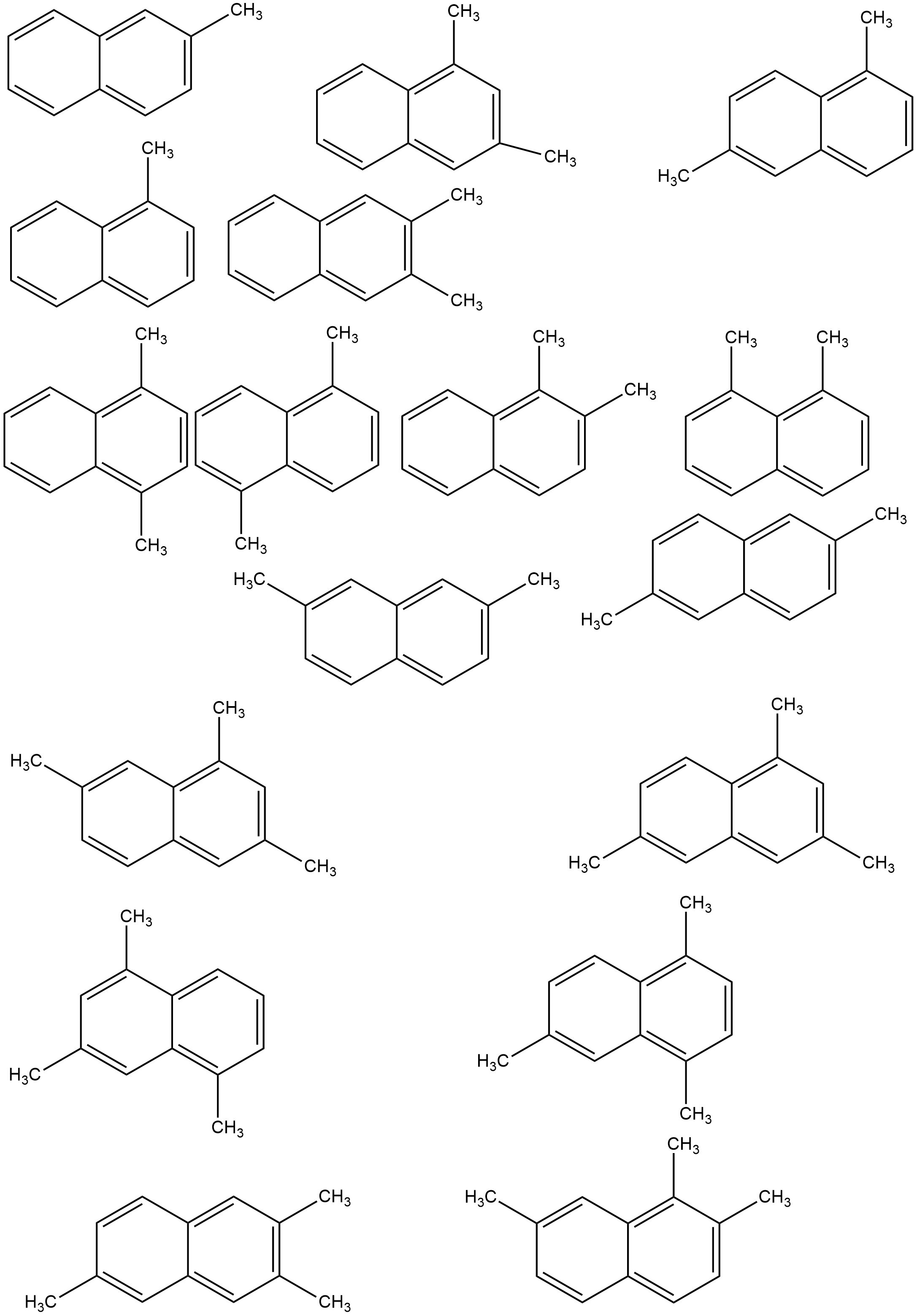  2,6-Dimethylnaphthalene  (2,6-DMN) | 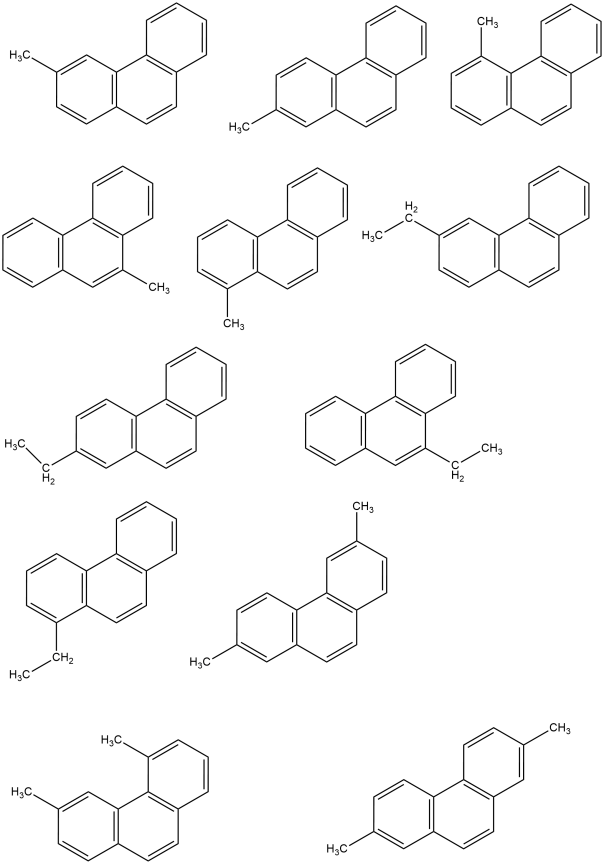  3,5-Dimethylphenanthrene (3,5-DMP) |  |
| 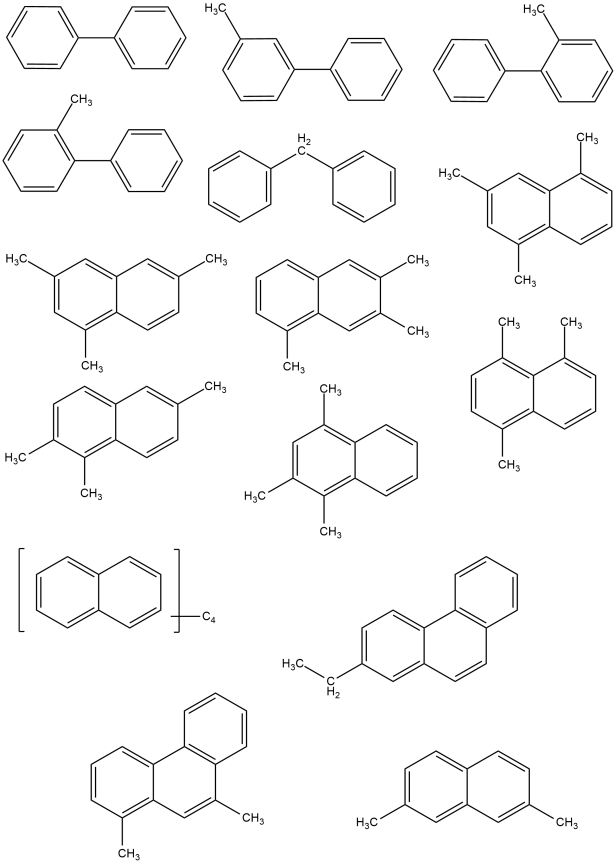  2,7-Dimethylnaphthalene  (2,7-DMN) | 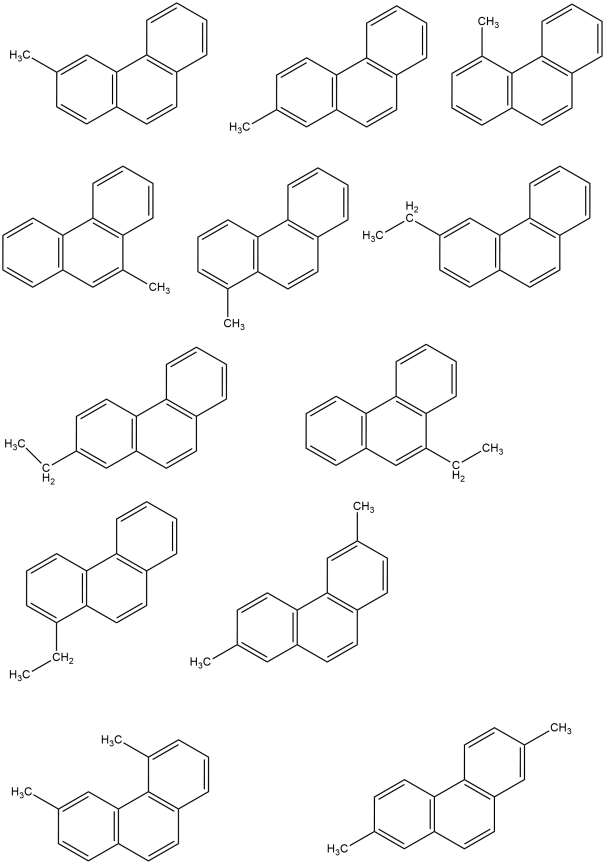  2,7-Dimethylphenanthrene (2,7-DMP) |  |
| 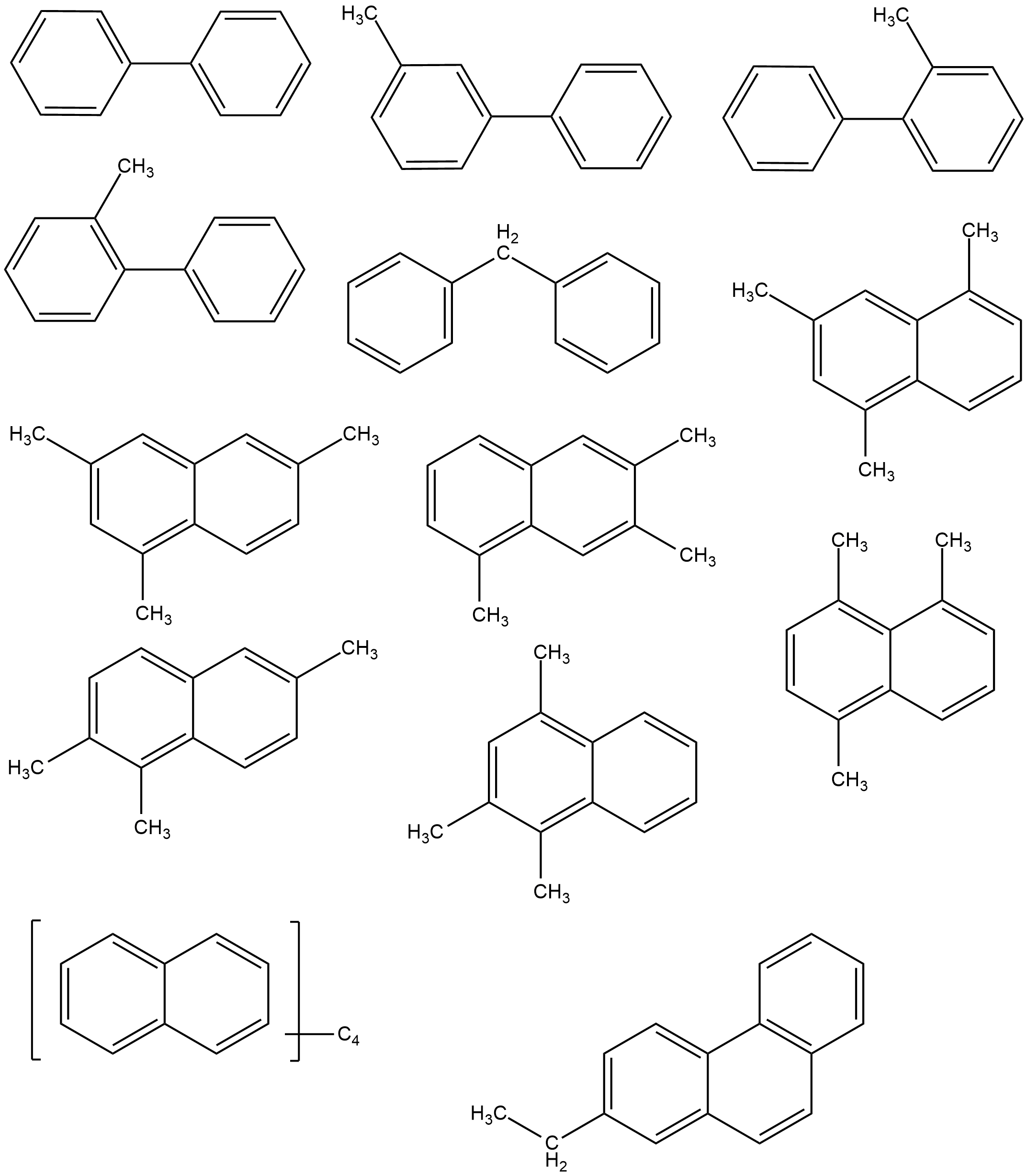  2-Methylbiphenyl (2-MBip) | 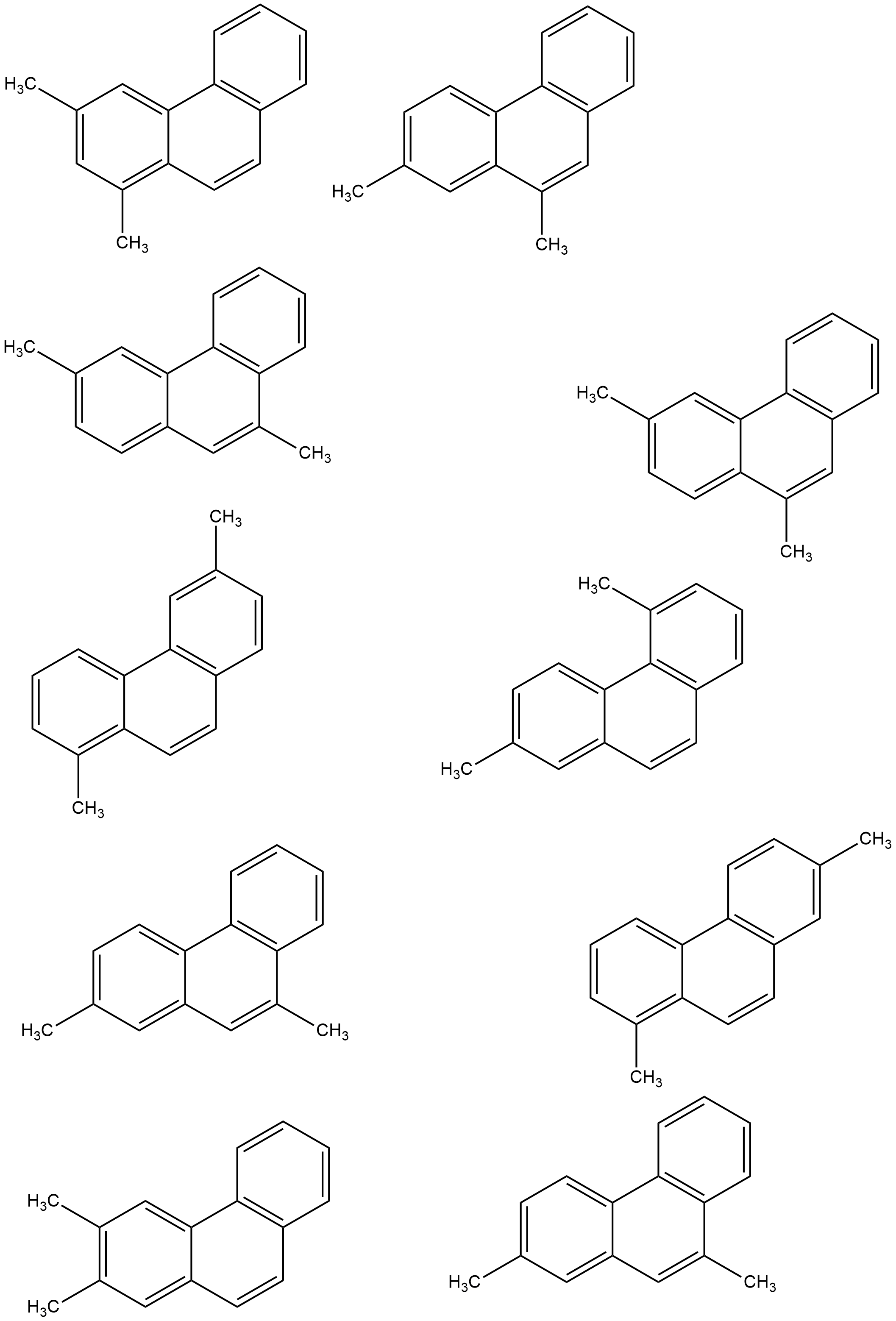  1,3-Dimethylphenanthrene (1,3-DMP) |  |
| 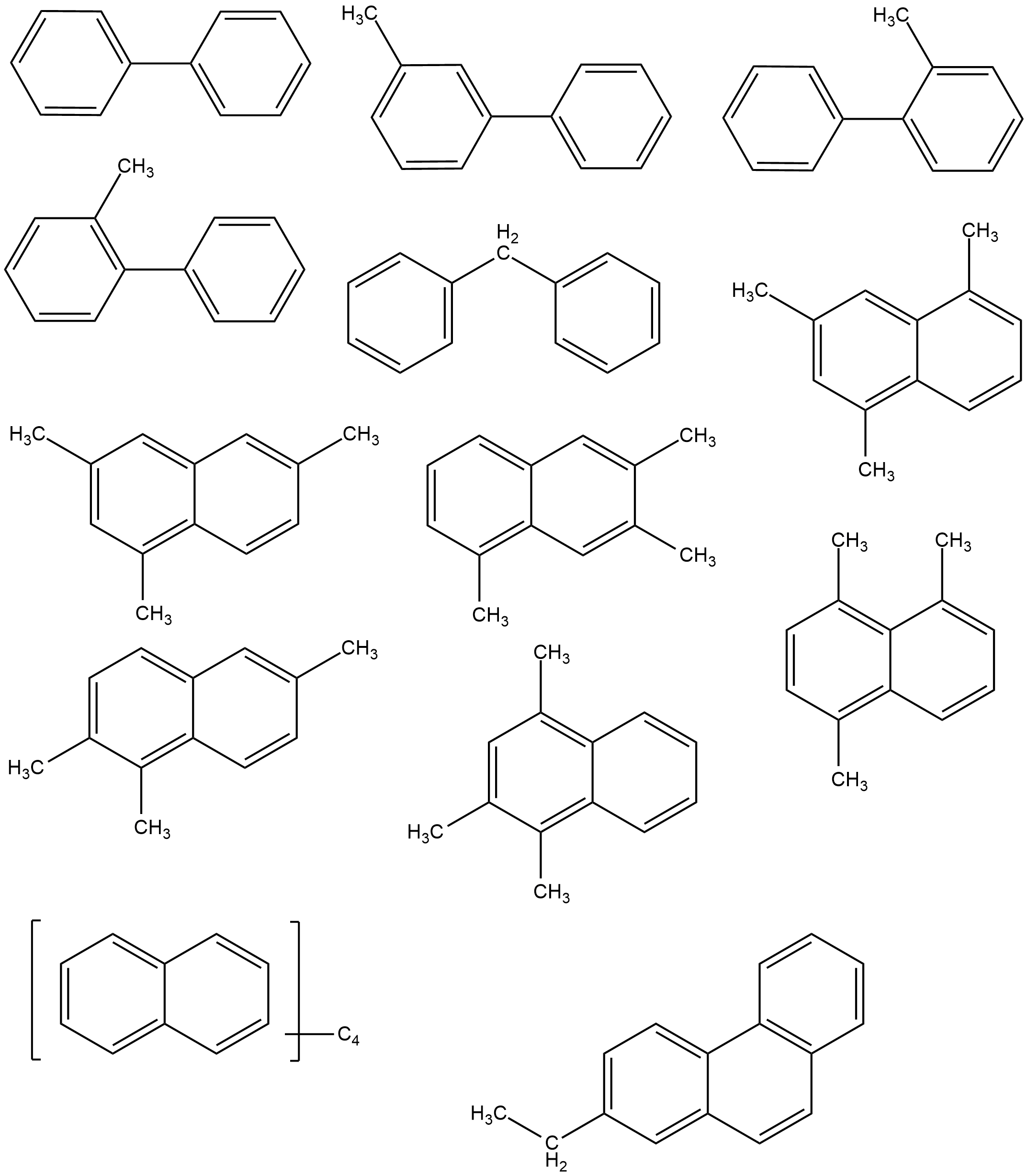  Diphenylmethane (DMe) | 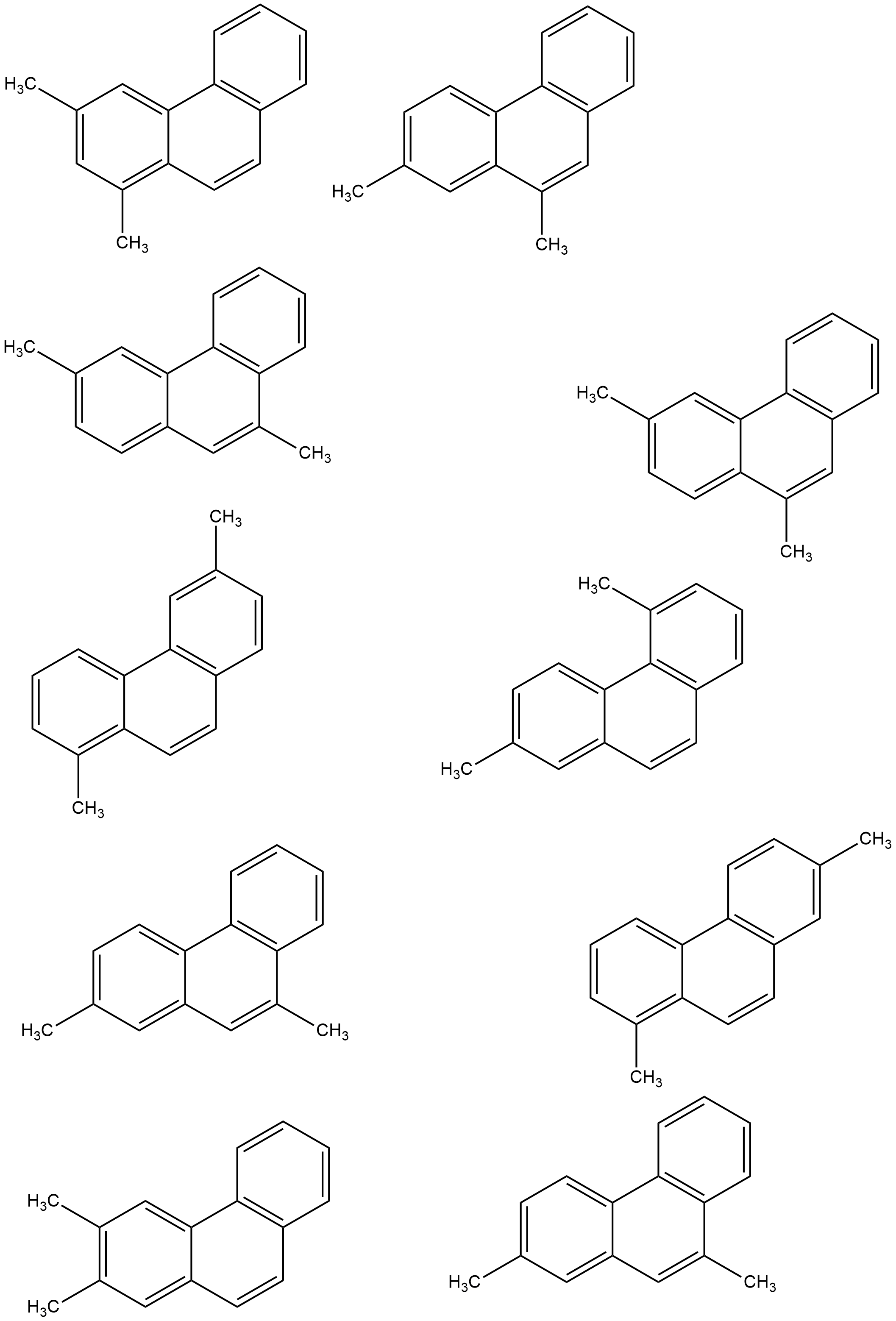  2,10- Dimethylphenanthrene (2,10-DMP) |  |
| 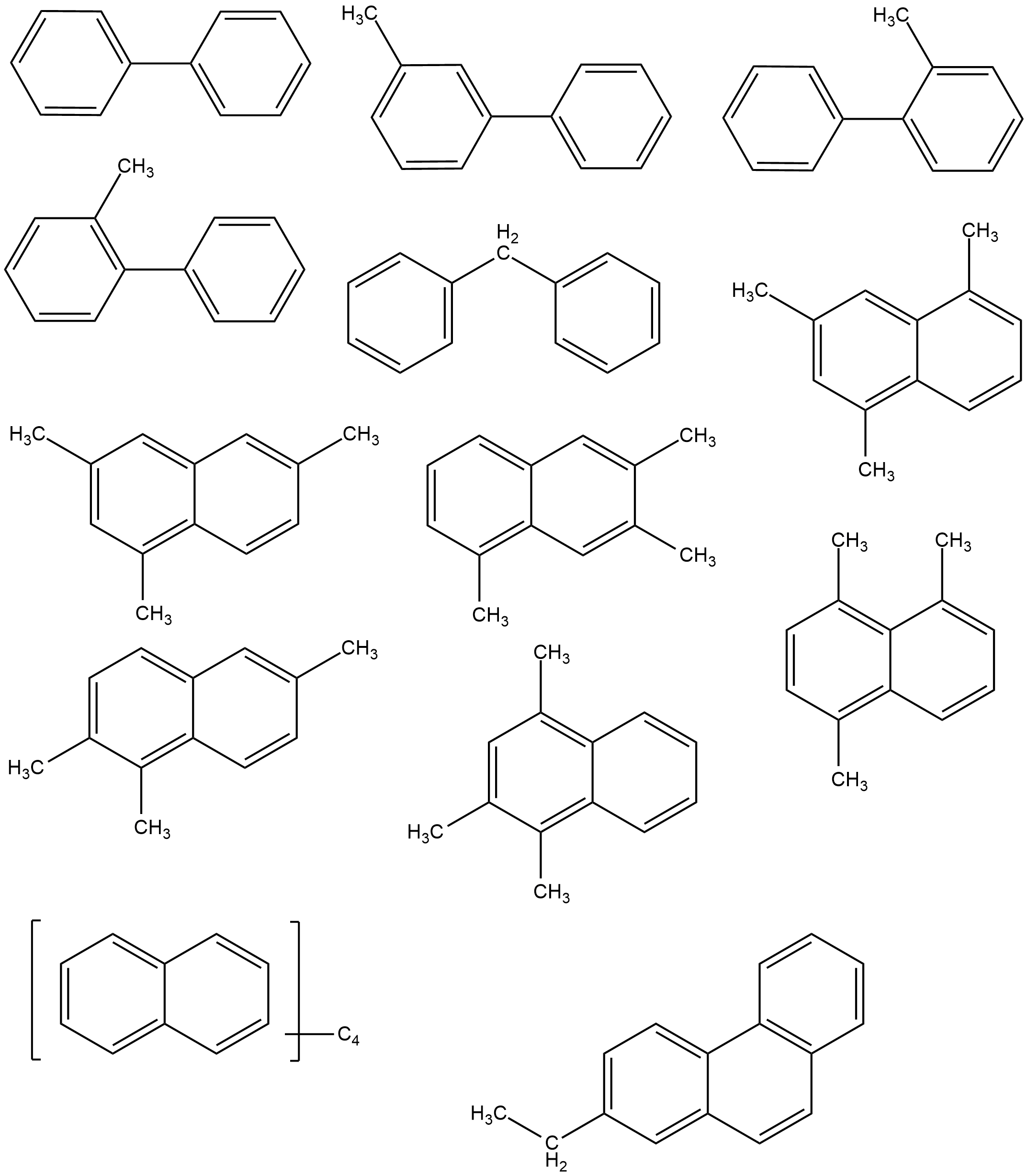  3-Methylbiphenyl (3-MBip) | 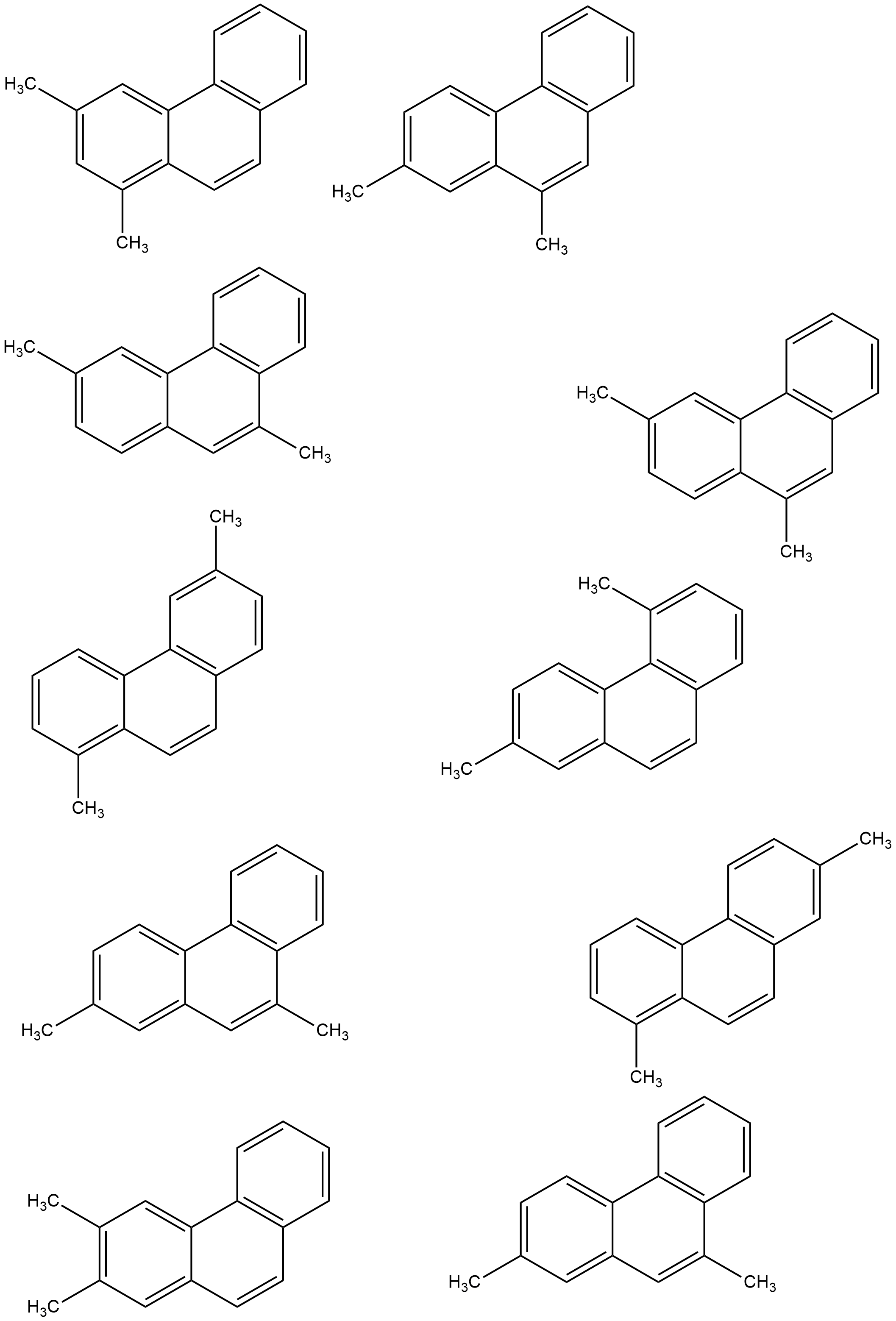  3,9- Dimethylphenanthrene (3,9-DMP) |  |
| 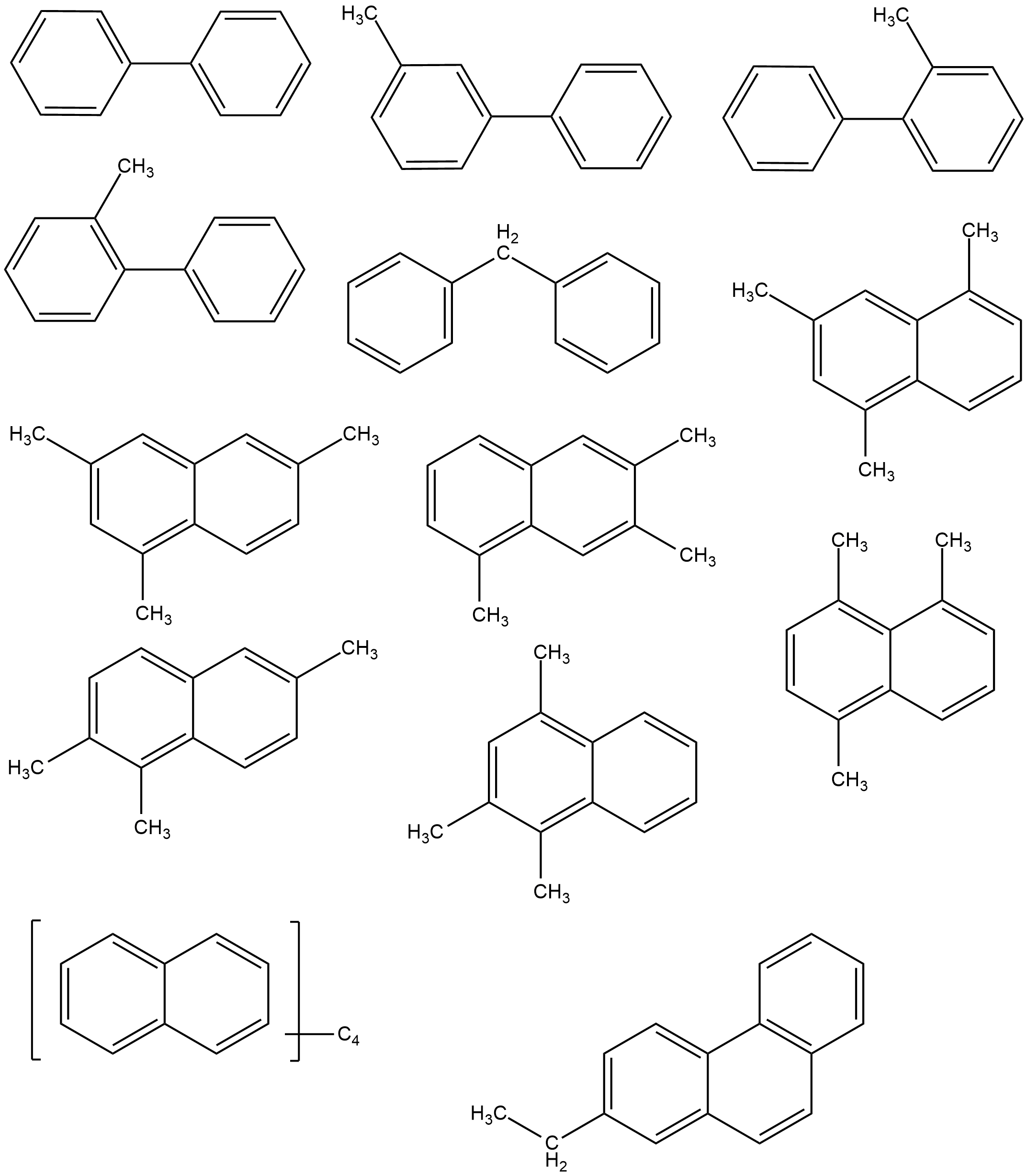  4-Methylbiphenyl (4-MBip) | 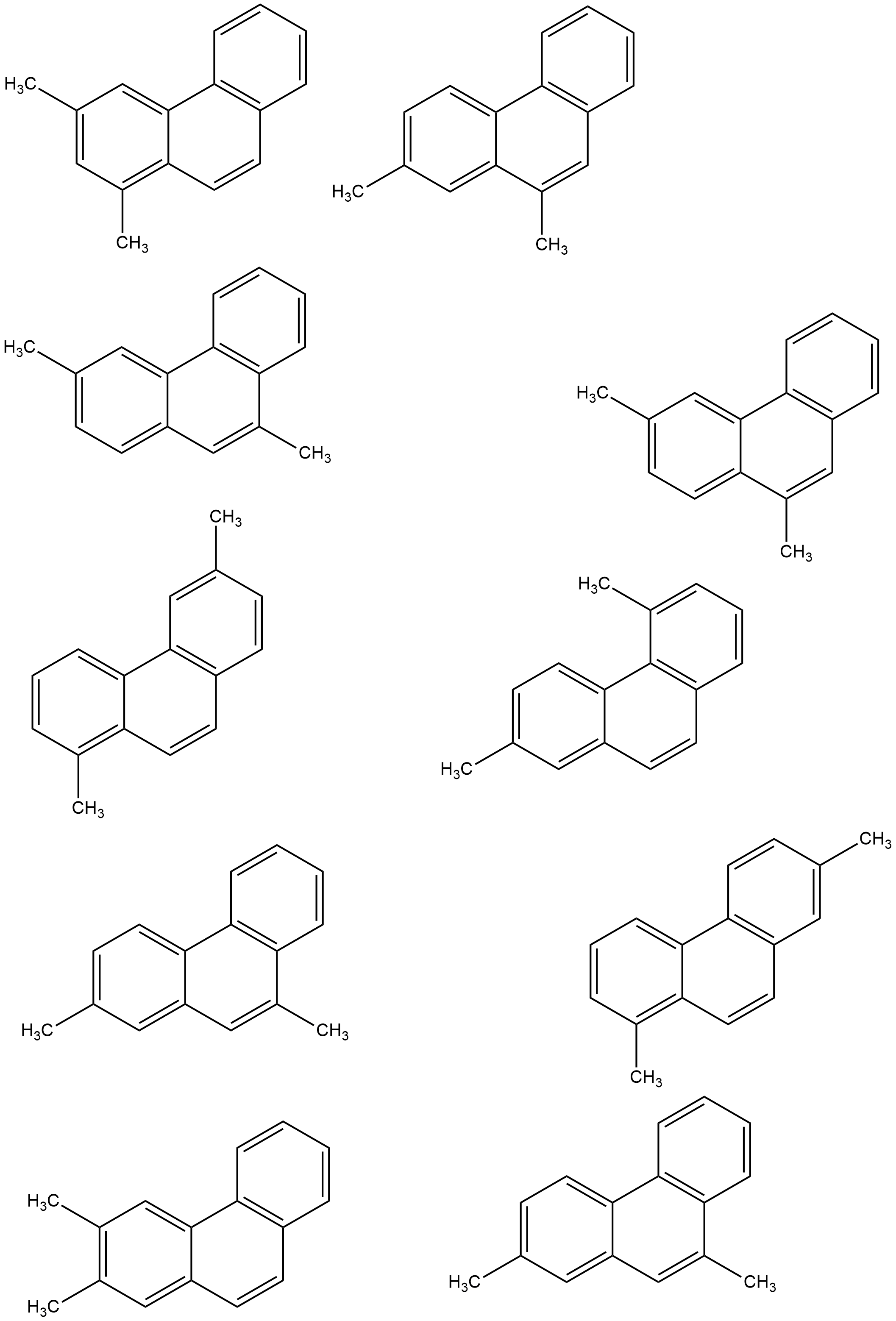  3,10-Dimethylphenanthrene (3,10-DMP) |  |
| 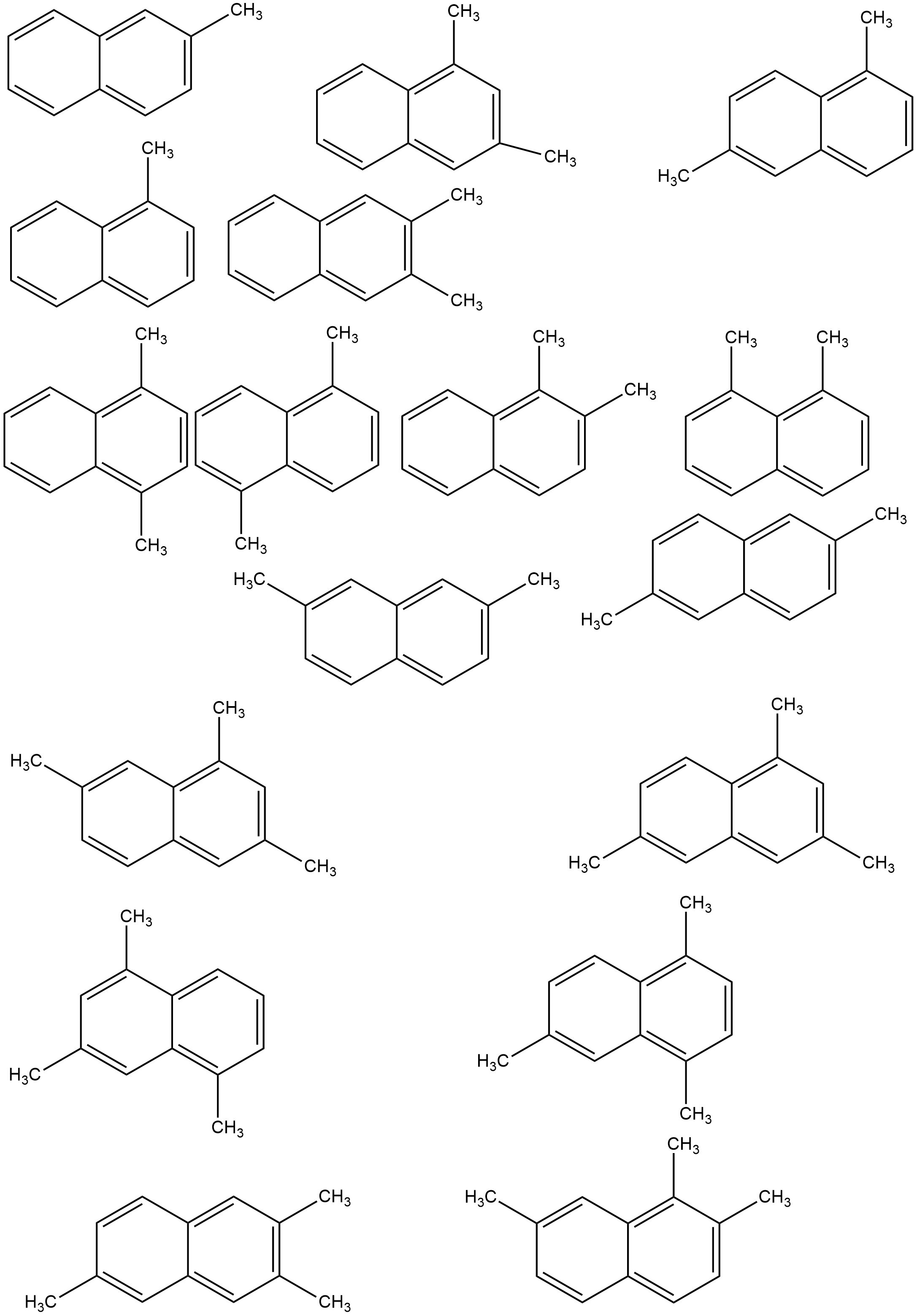  1,3,7-Trimethylnaphthalene (1,3,7-TMN) | 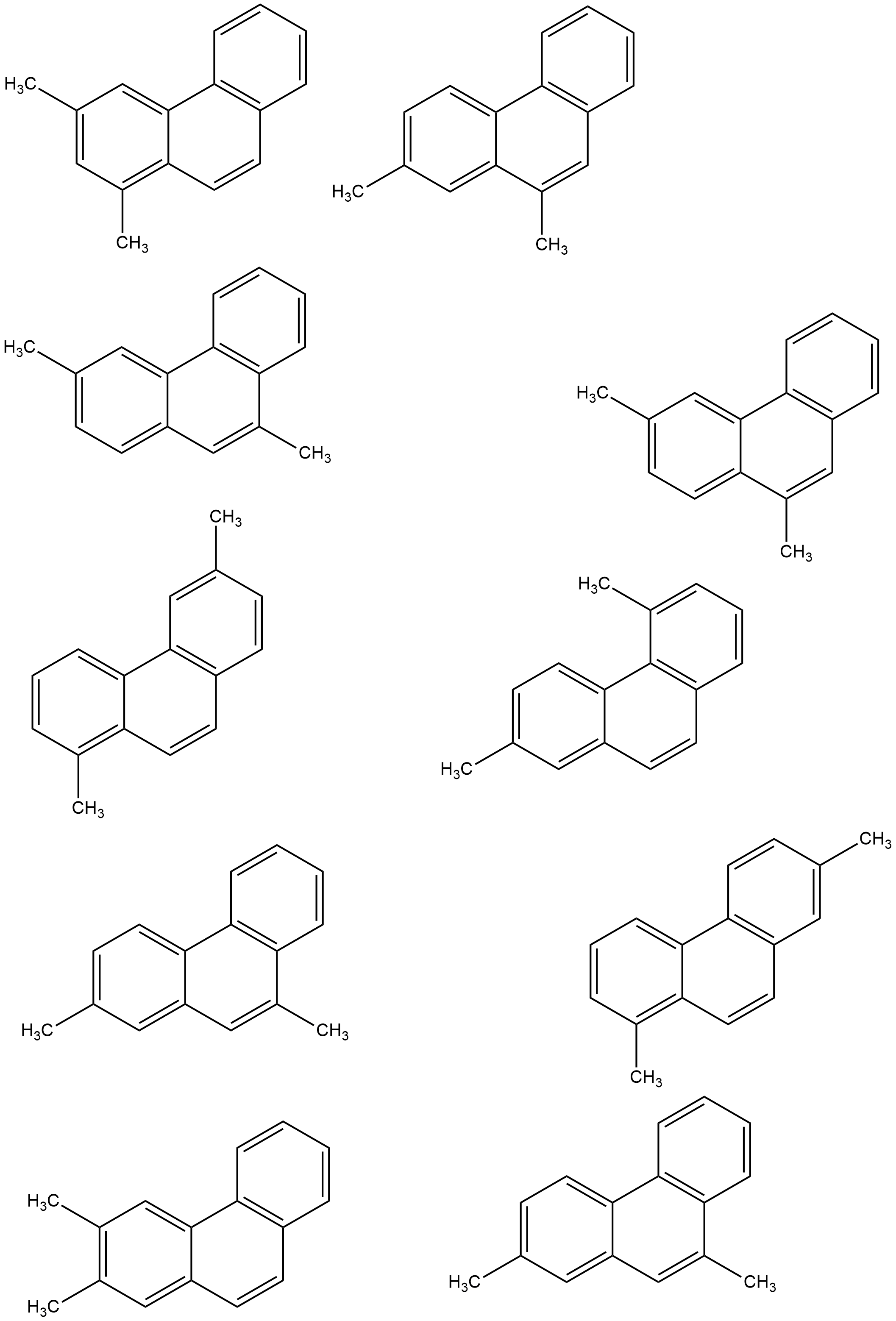  1,6-Dimethylphenanthrene (1,6-DMP) |  |
| 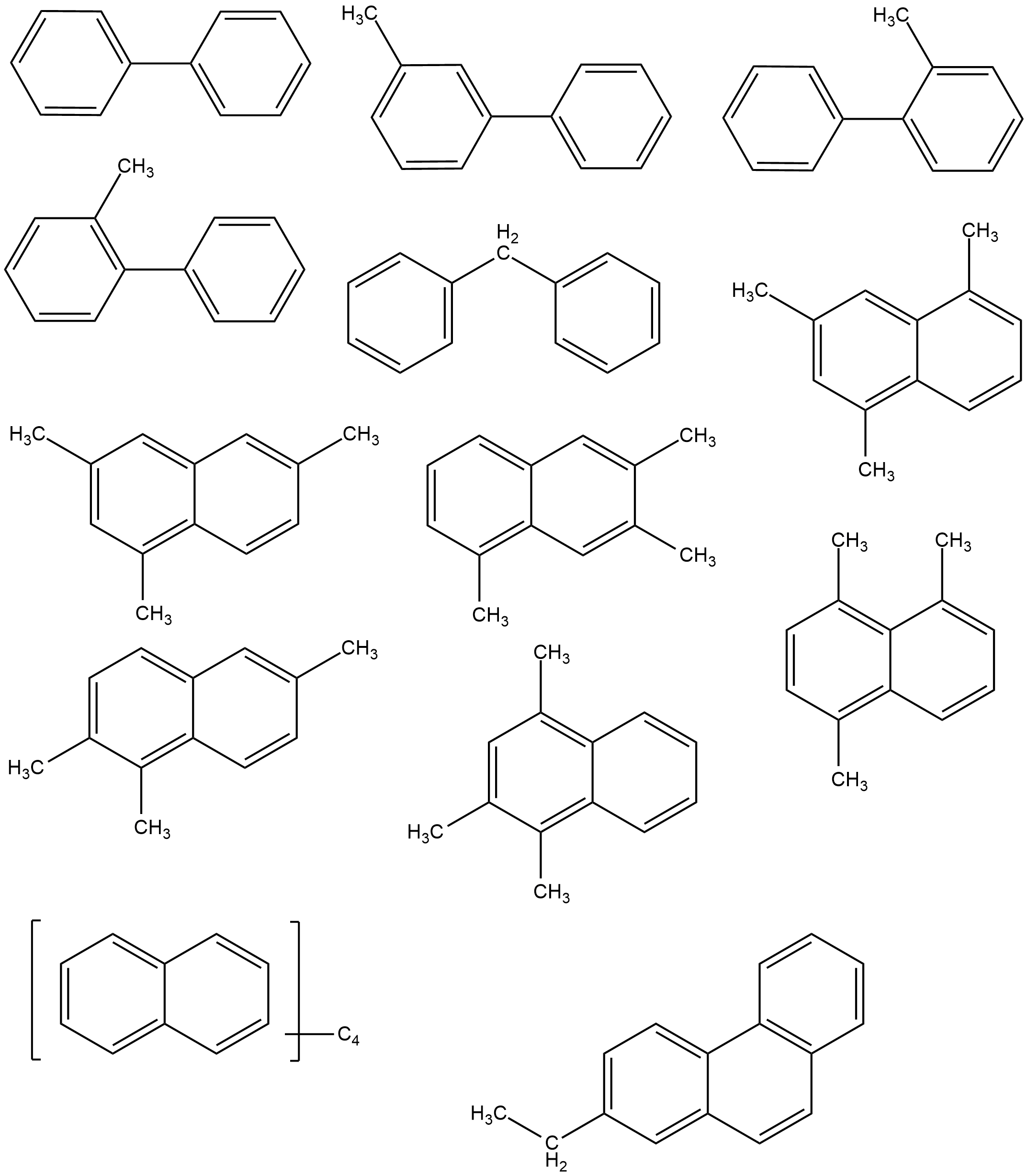  1,3,6-Trimethylnaphthalene (1,3,6-TMN) | 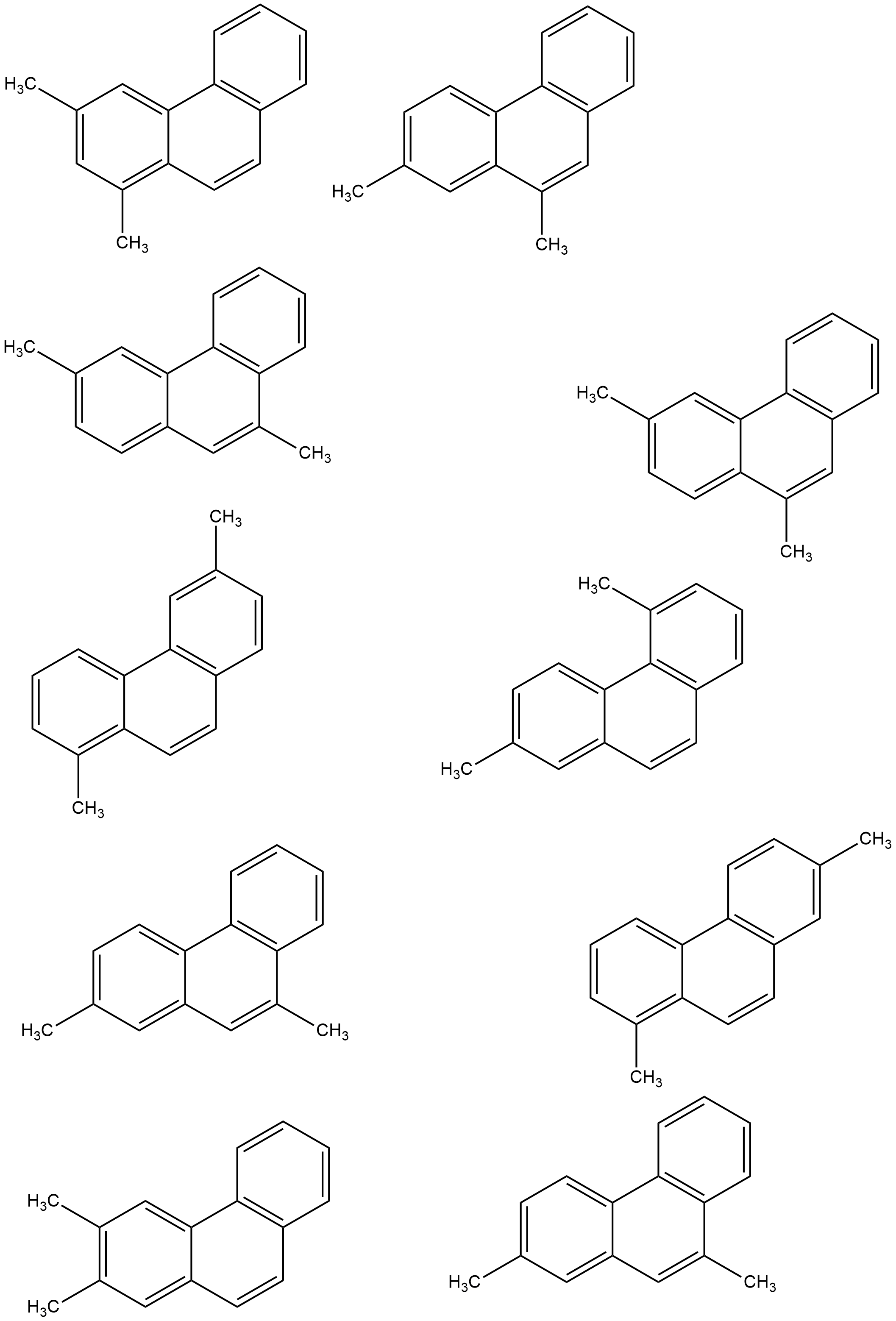  2,5-Dimethylphenanthrene (2,5-DMP) |  |
| 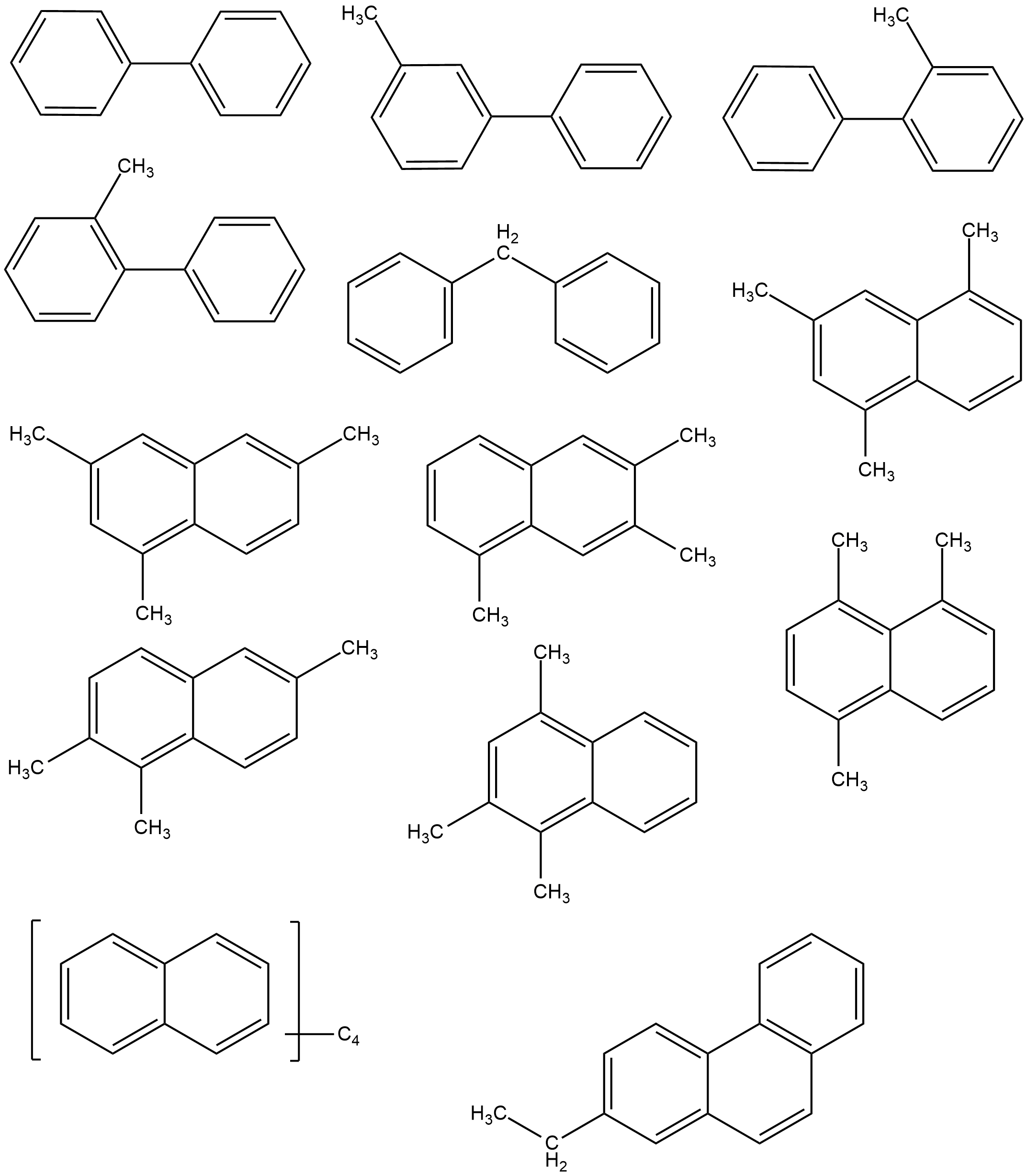  1,3,5-Trimethylnaphthalene (1,3,5-TMN) | 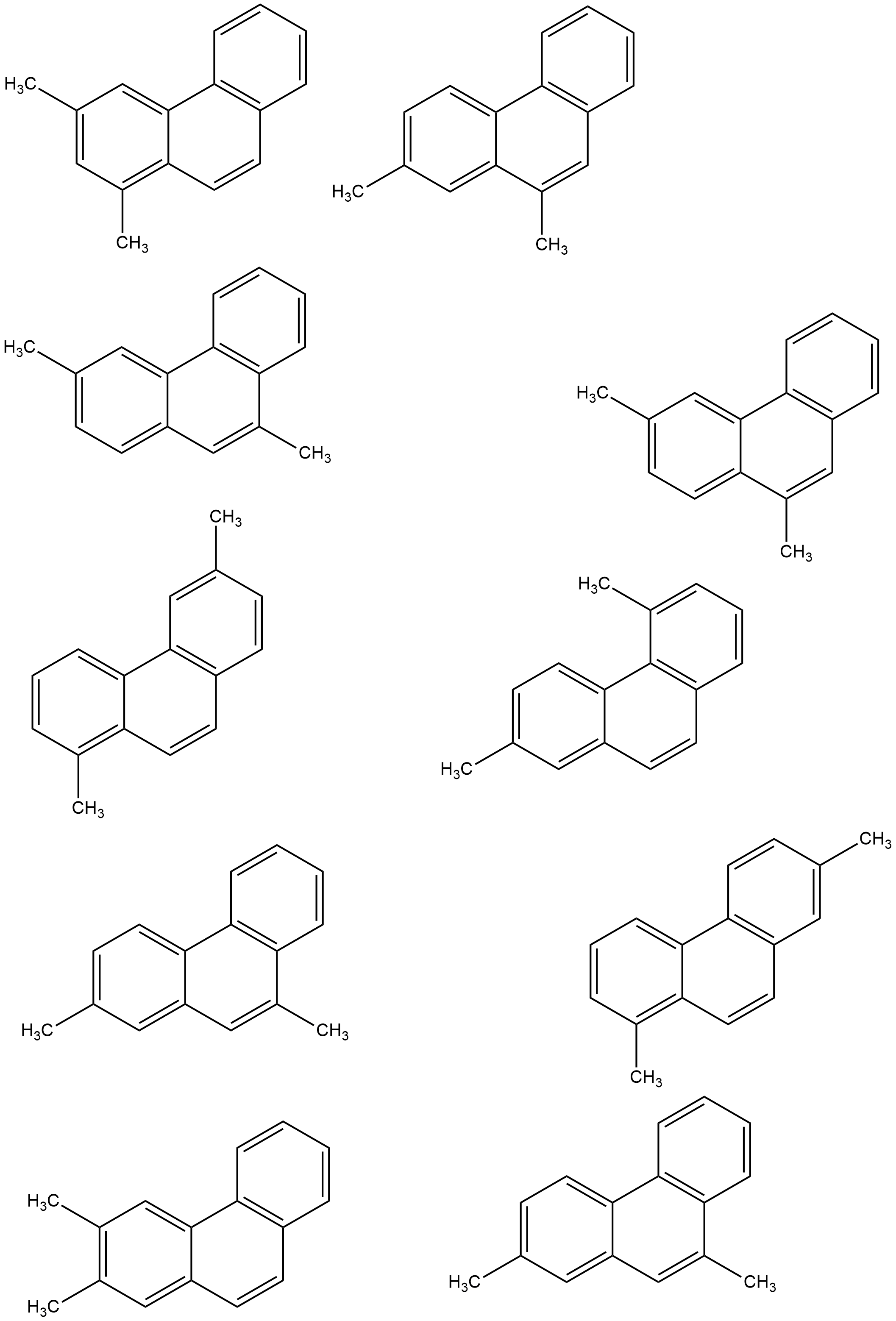  2,9-Dimethylphenanthrene (2,9-DMP) |  |
| 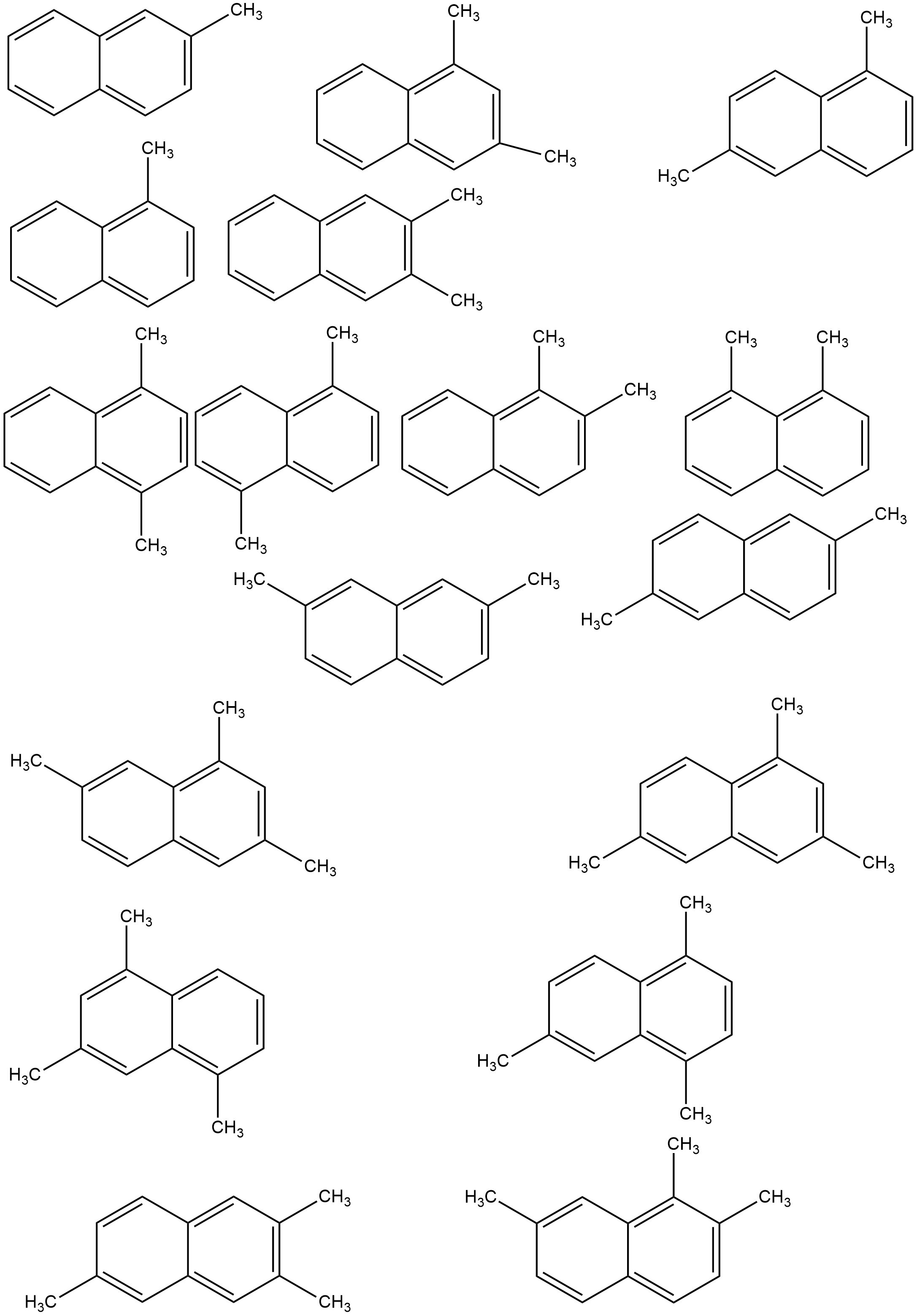  1,4,6- Trimethylnaphthalene (1,4,6- TMN) | 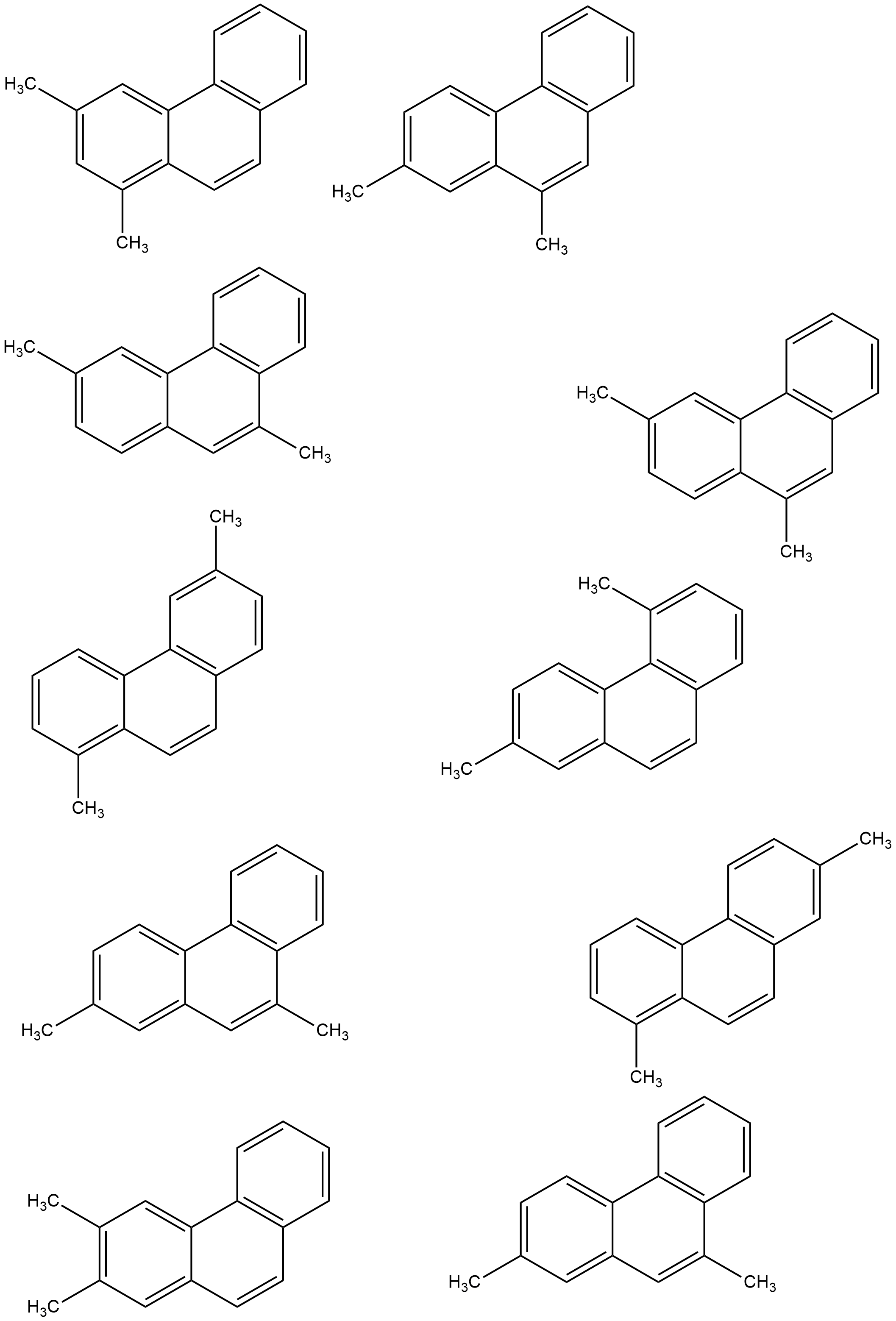  1,7-Dimethylphenanthrene (1,7-DMP) |  |
| 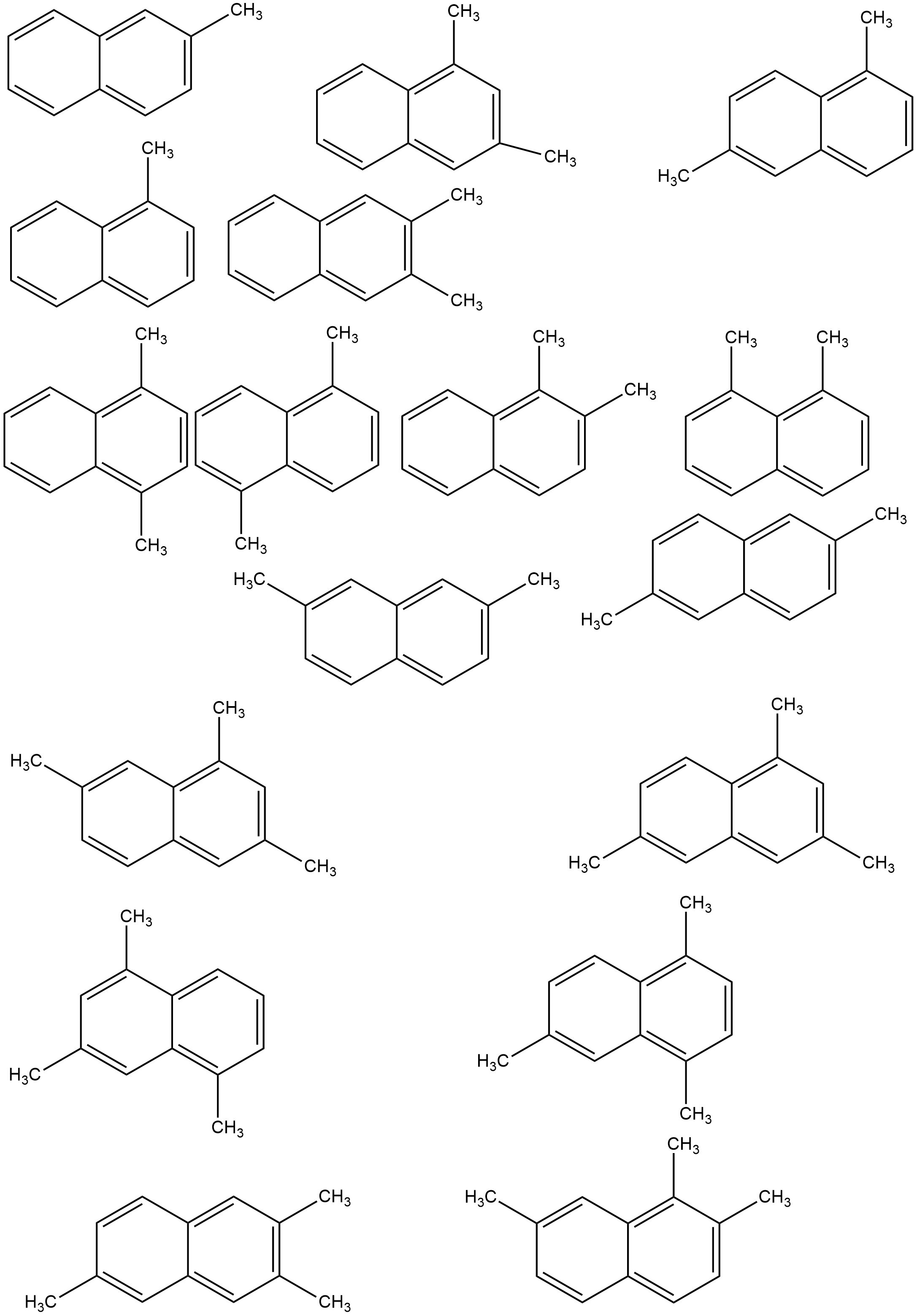  2,3,6-Trimethylnaphthalene (2,3,6-TMN) | 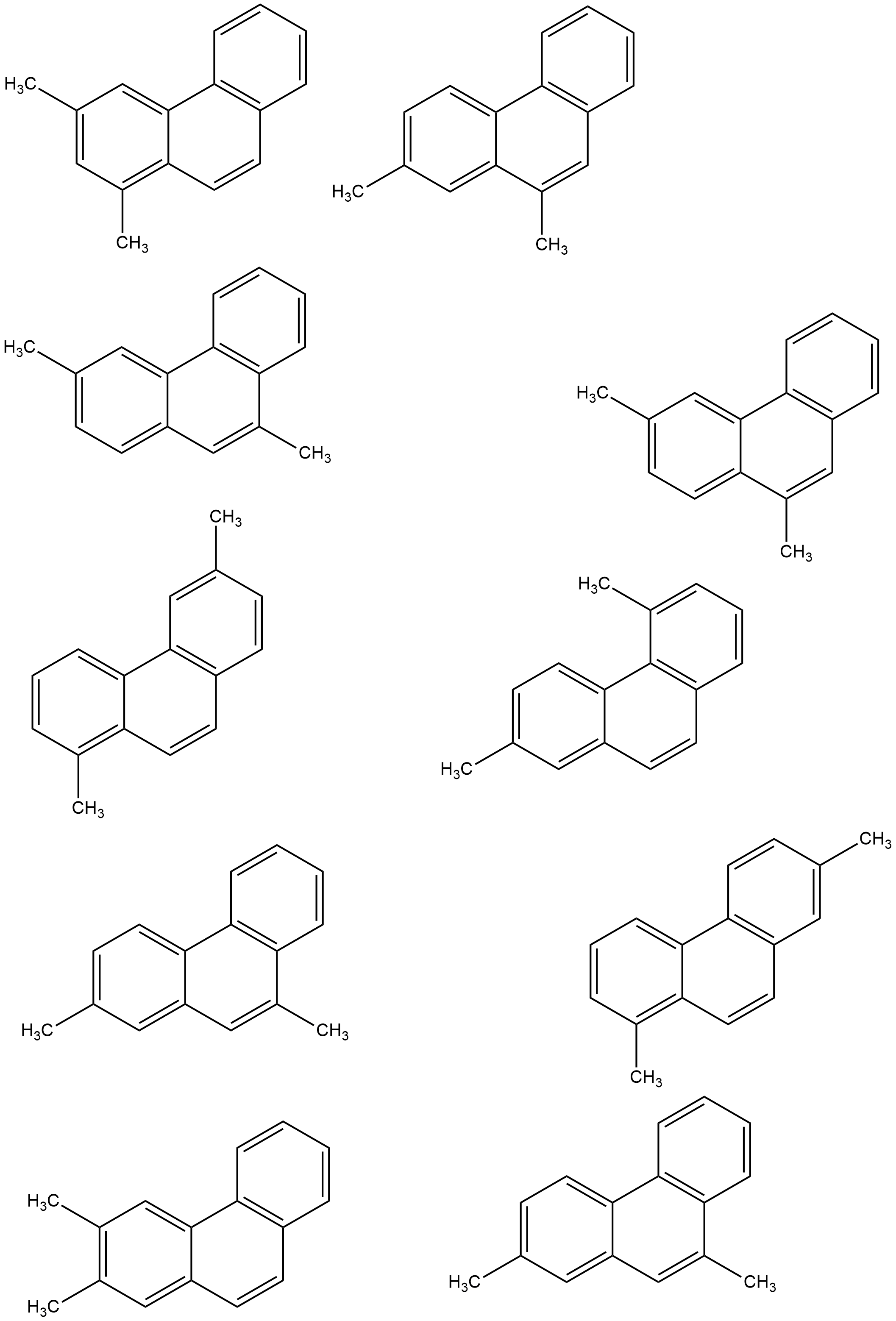  2,3-Dimethylphenanthrene (2,3-DMP) |  |
| 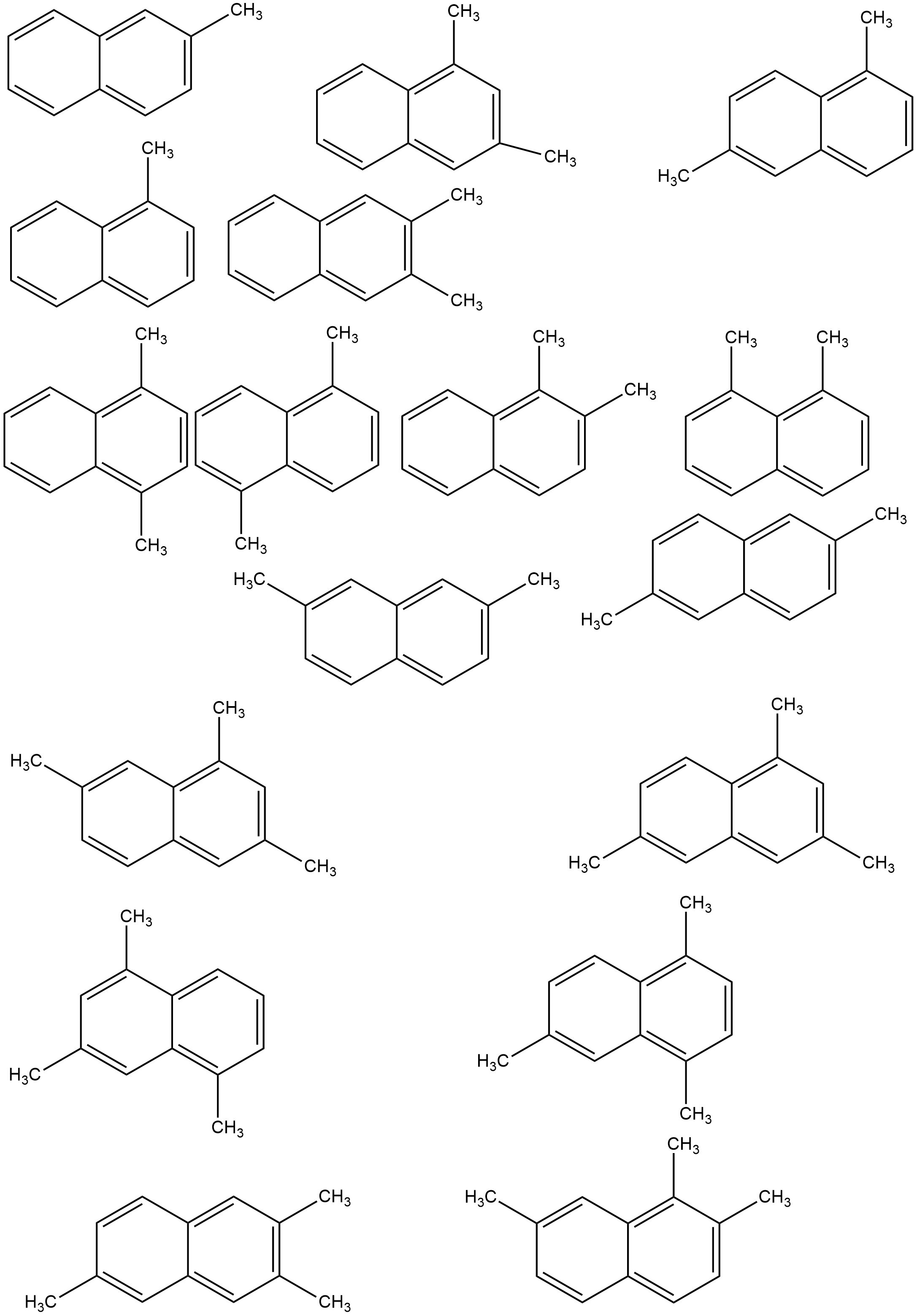  1,2,7-Trimethylnaphthalene (1,2,7-TMN) | 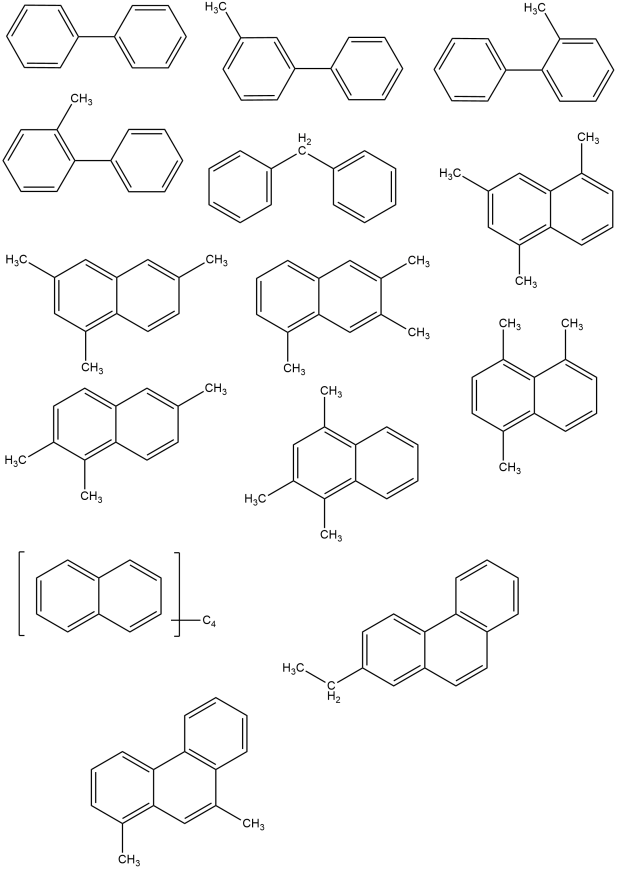  1,9-Dimethylphenanthrene (1,9-DMP) |  |
| 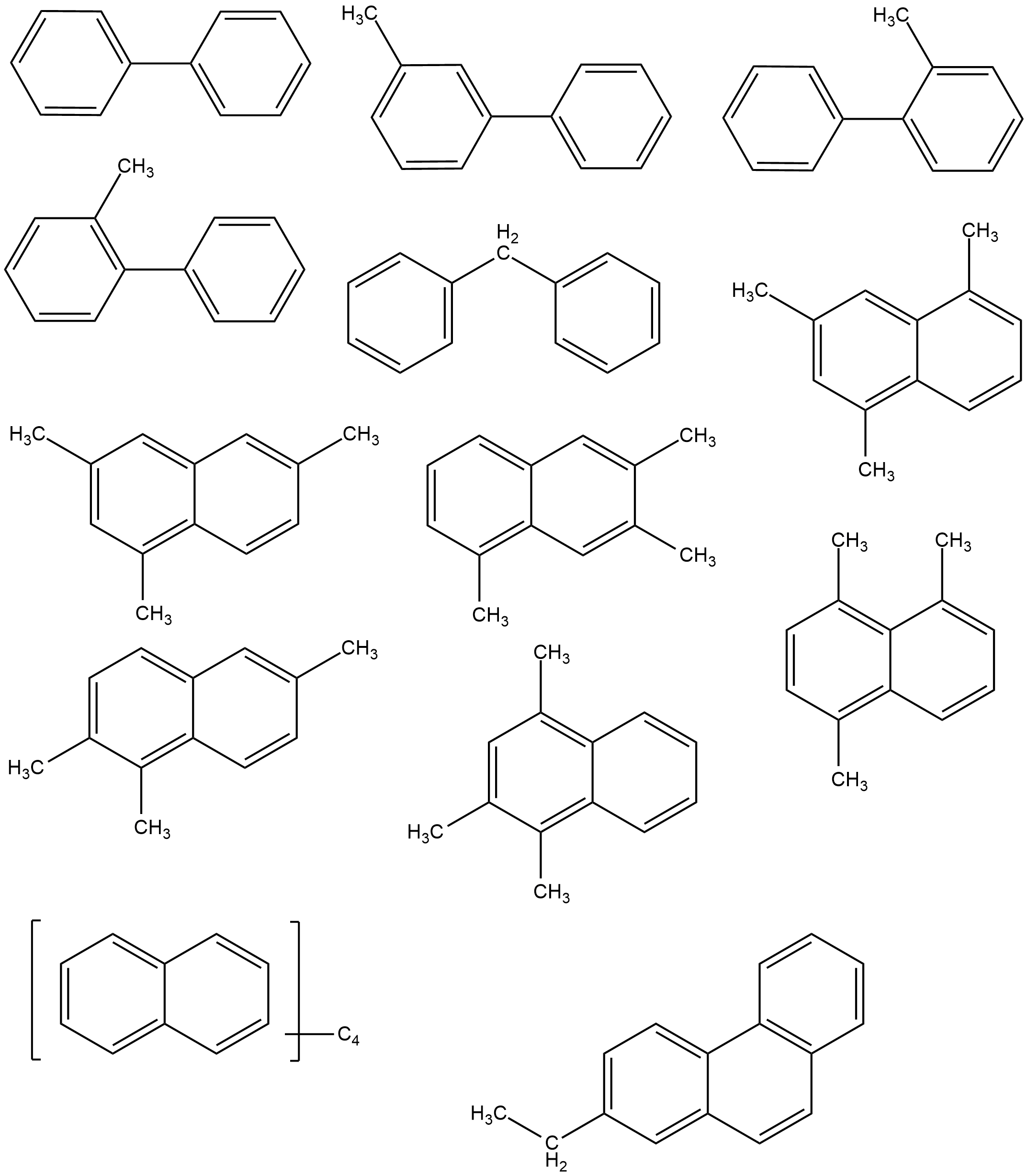  1,6,7-Trimethylnaphthalene (1,6,7-TMN) | 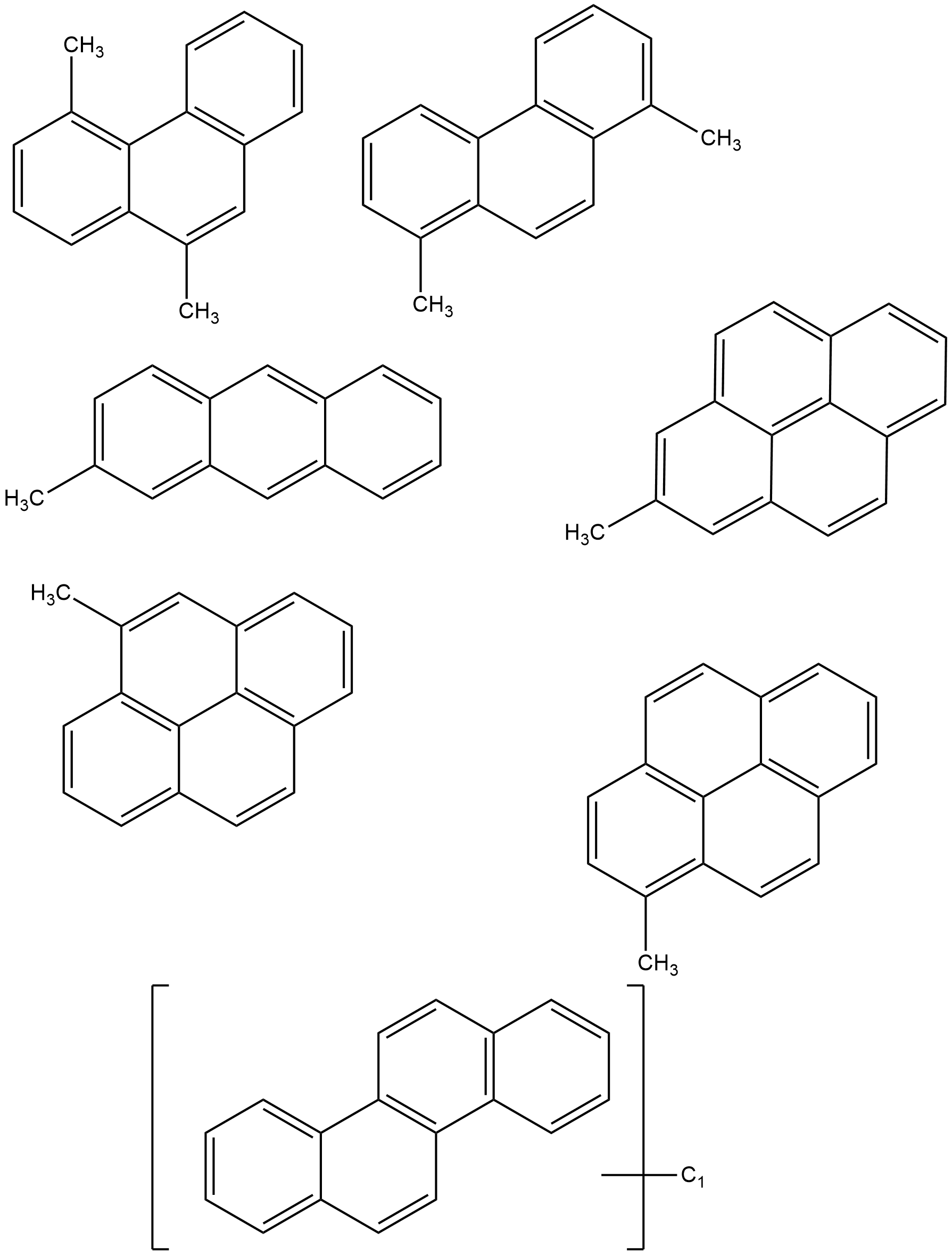  4,10-Dimethylphenanthrene (4,10-DMP) |  |
| 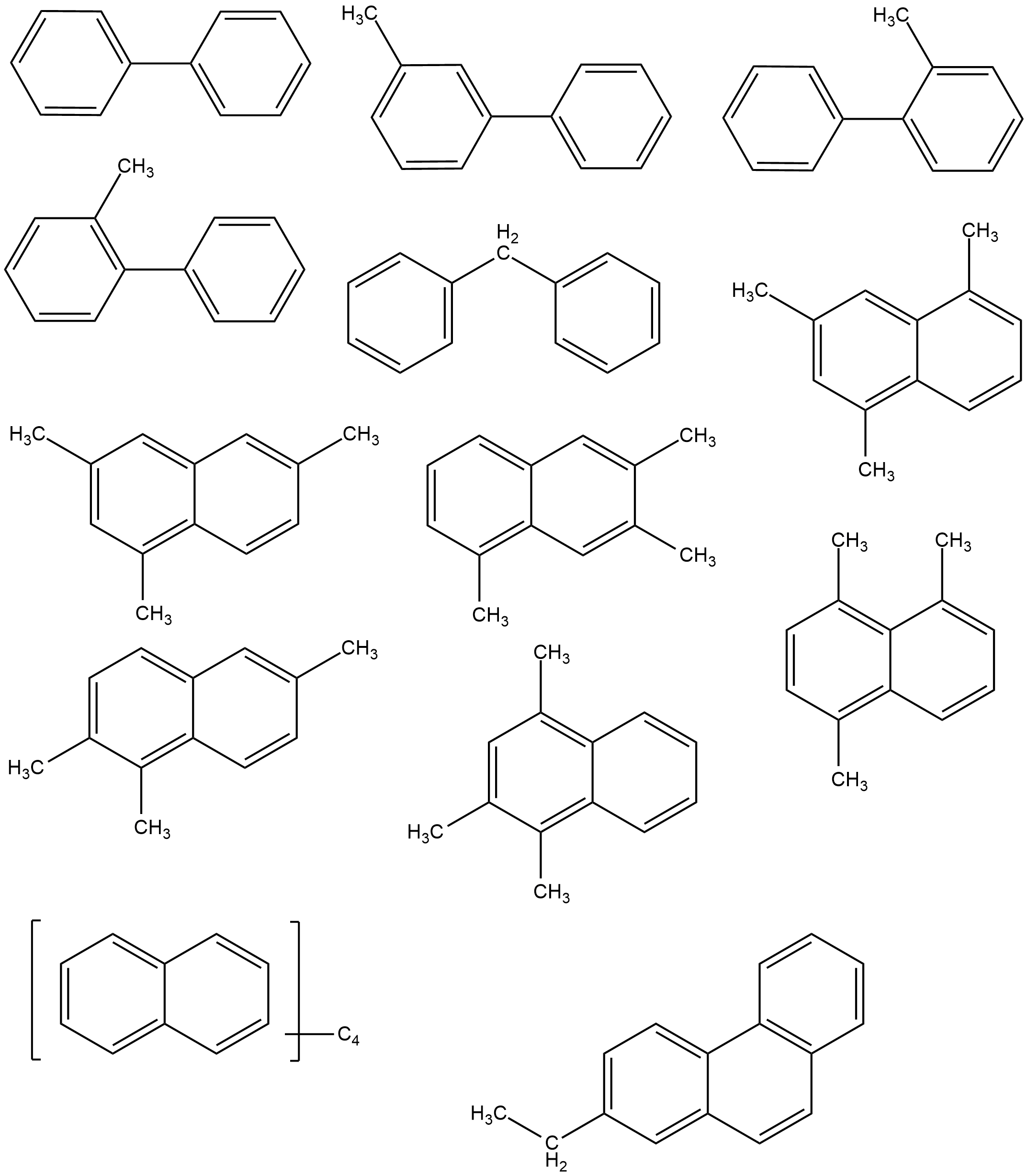  1,2,6-Trimethylnaphthalene (1,2,6-TMN) | 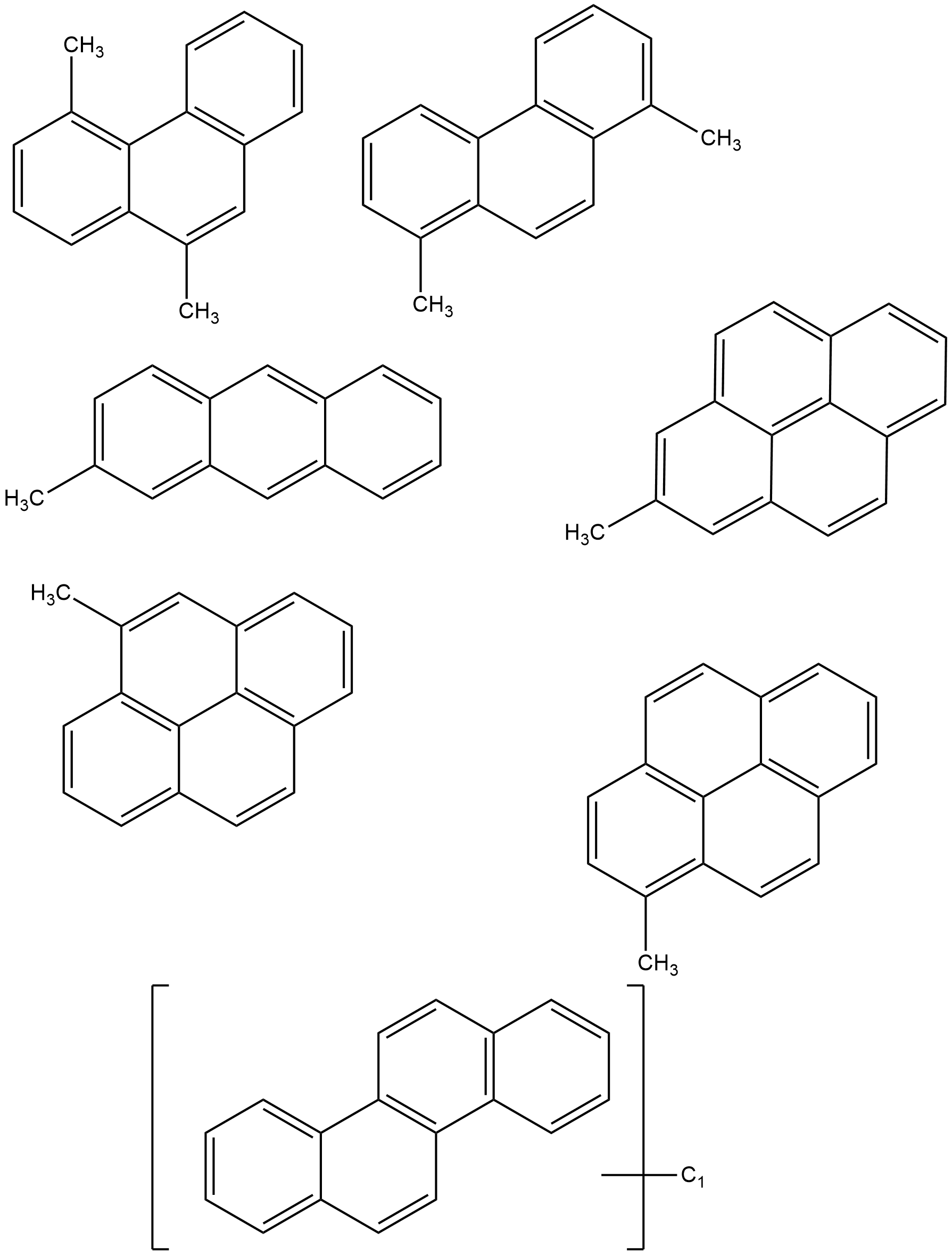  1,8-Dimethylphenanthrene (1,8-DMP) |  |
| 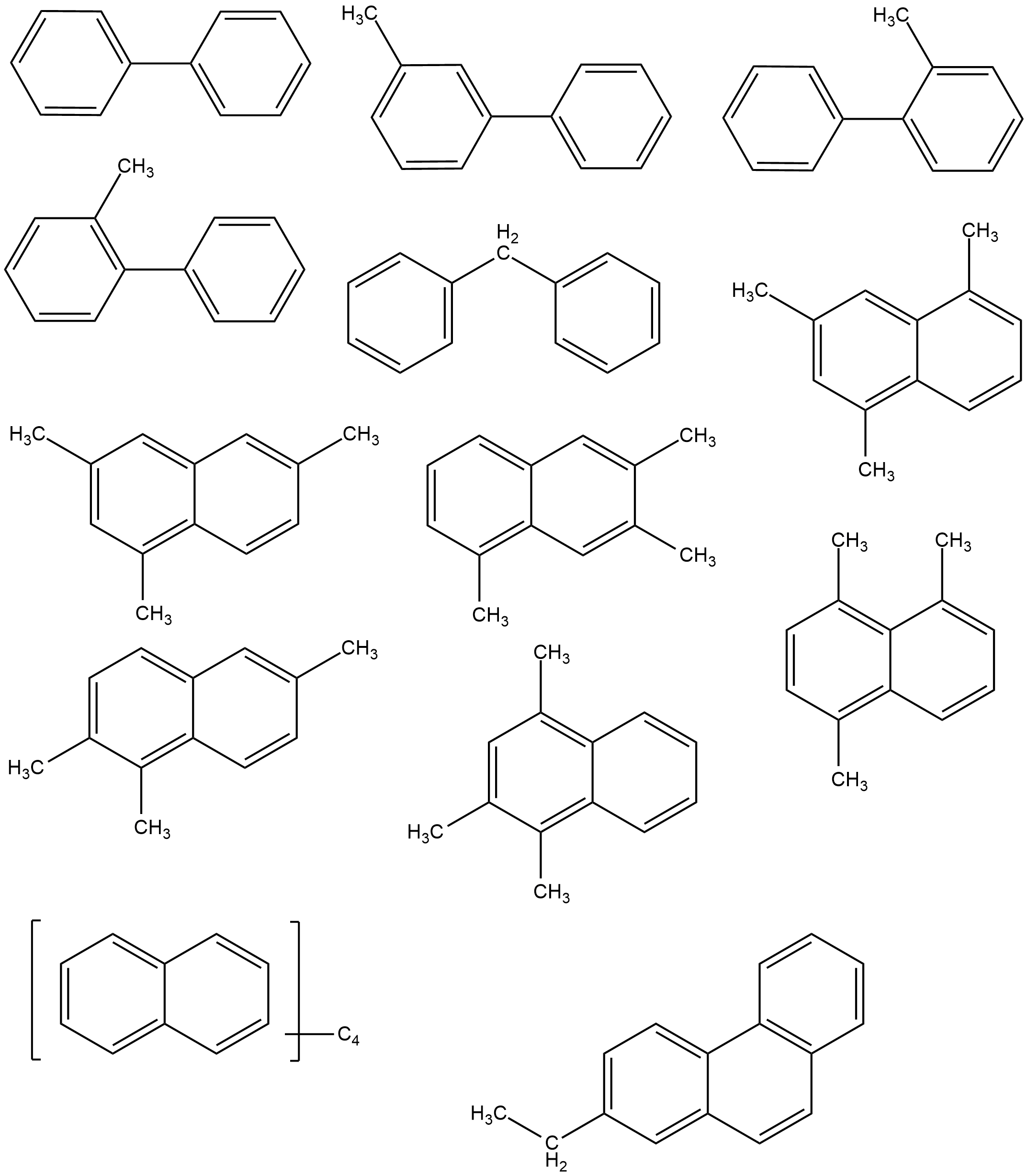  1,2,4-Trimethylnaphthalene (1,2,4-TMN) | 1,2,3-Trimethyl-4-propenylnaphthalene (1,2,3-TMPN) |  |
| 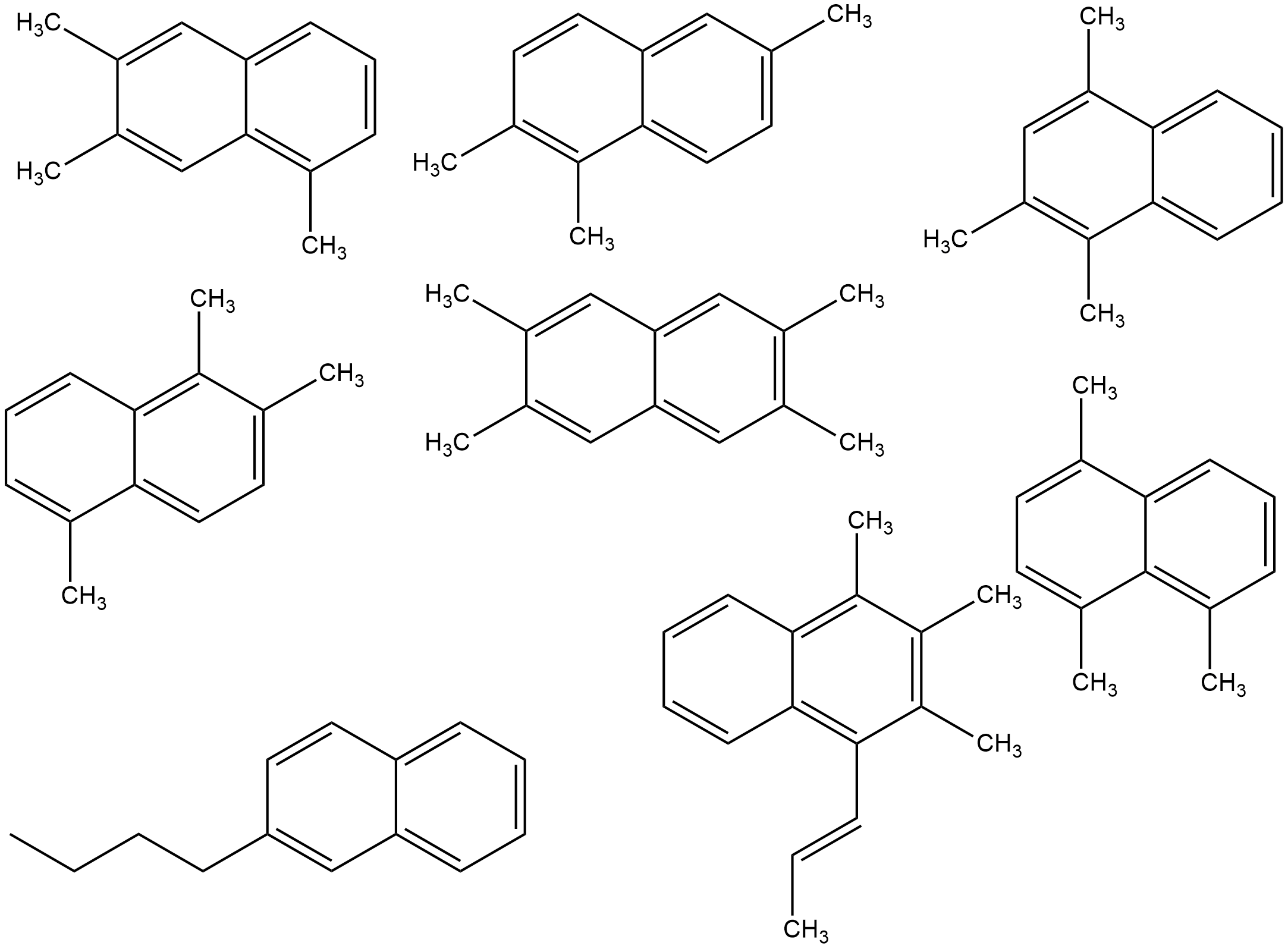  1,2,5-Trimethylnaphthalene (1,2,5-TMN) | 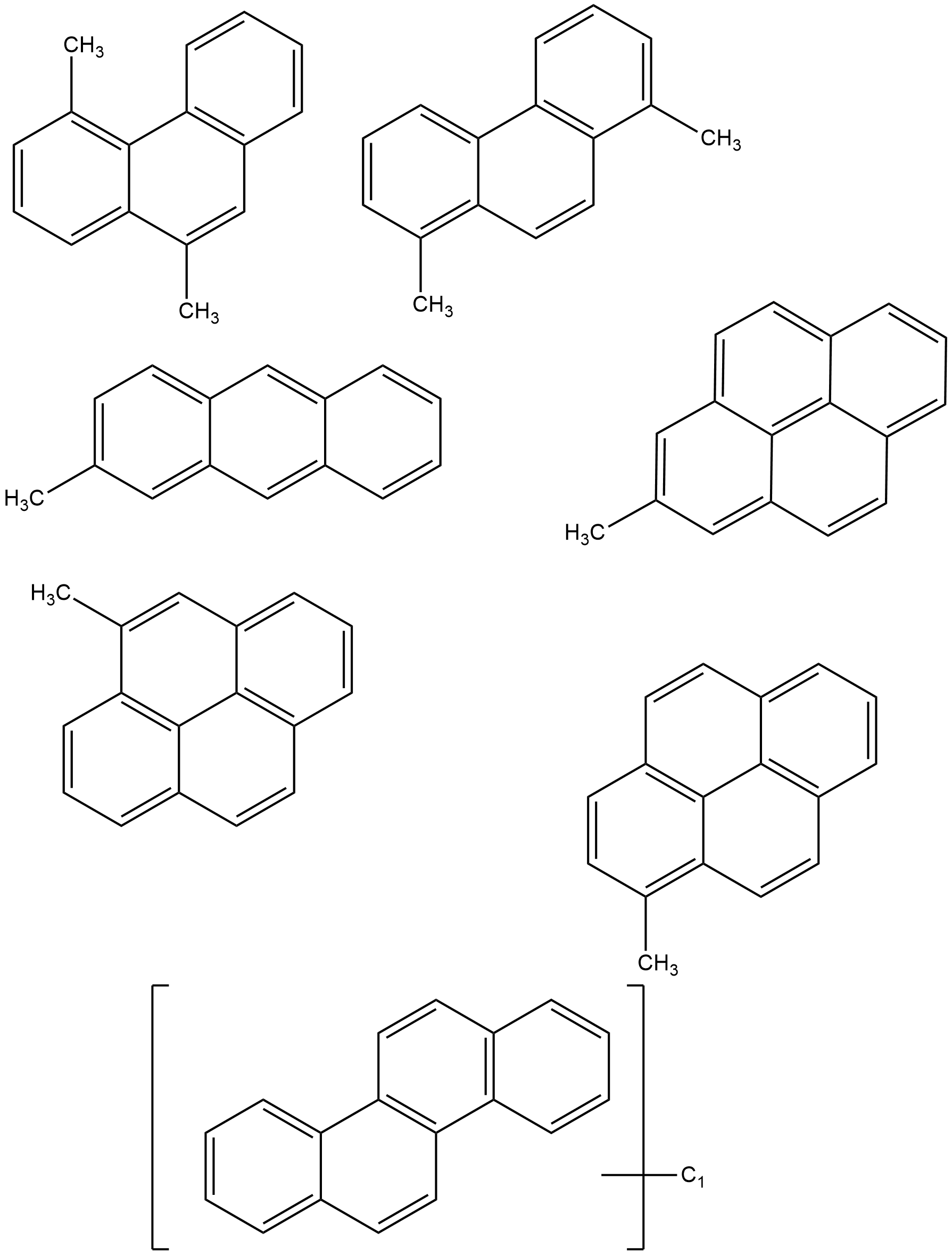Methylchrysene (MChr) |  |
| 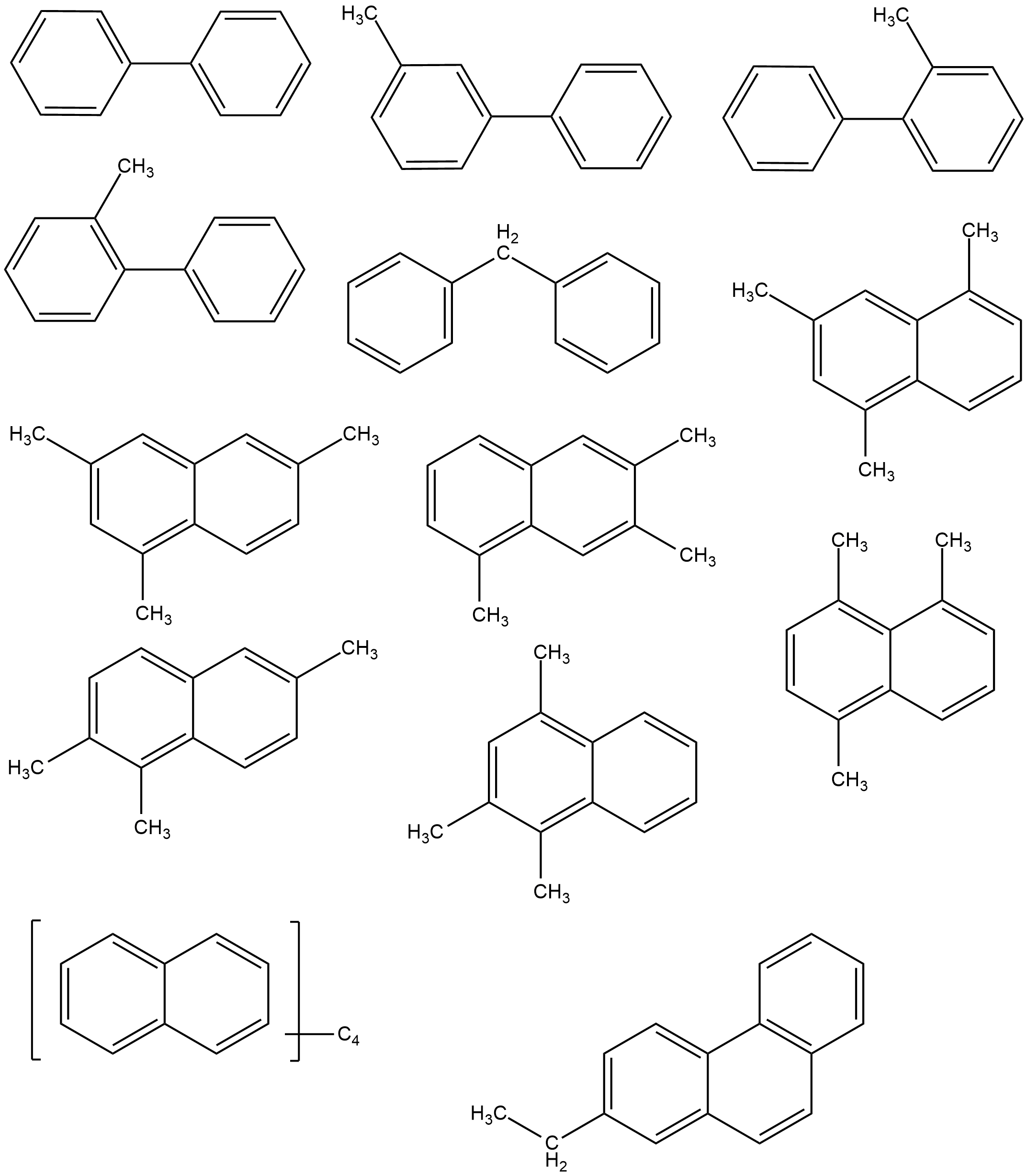  1,4,5-Trimethylnaphthalene (1,4,5-TMN) |  |  |
| 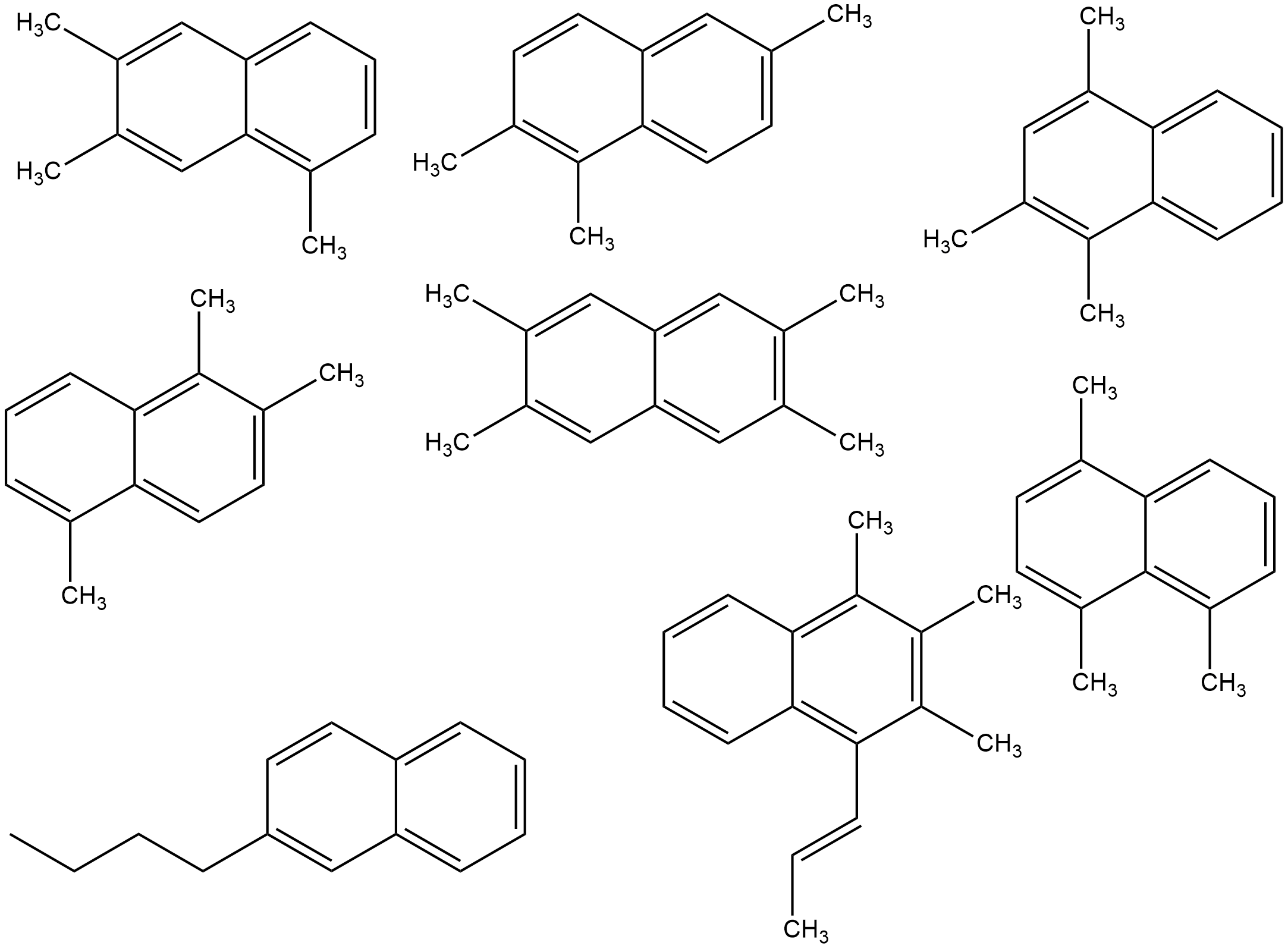  2-Butylnaphthalene (2-BN) |  |  |
| 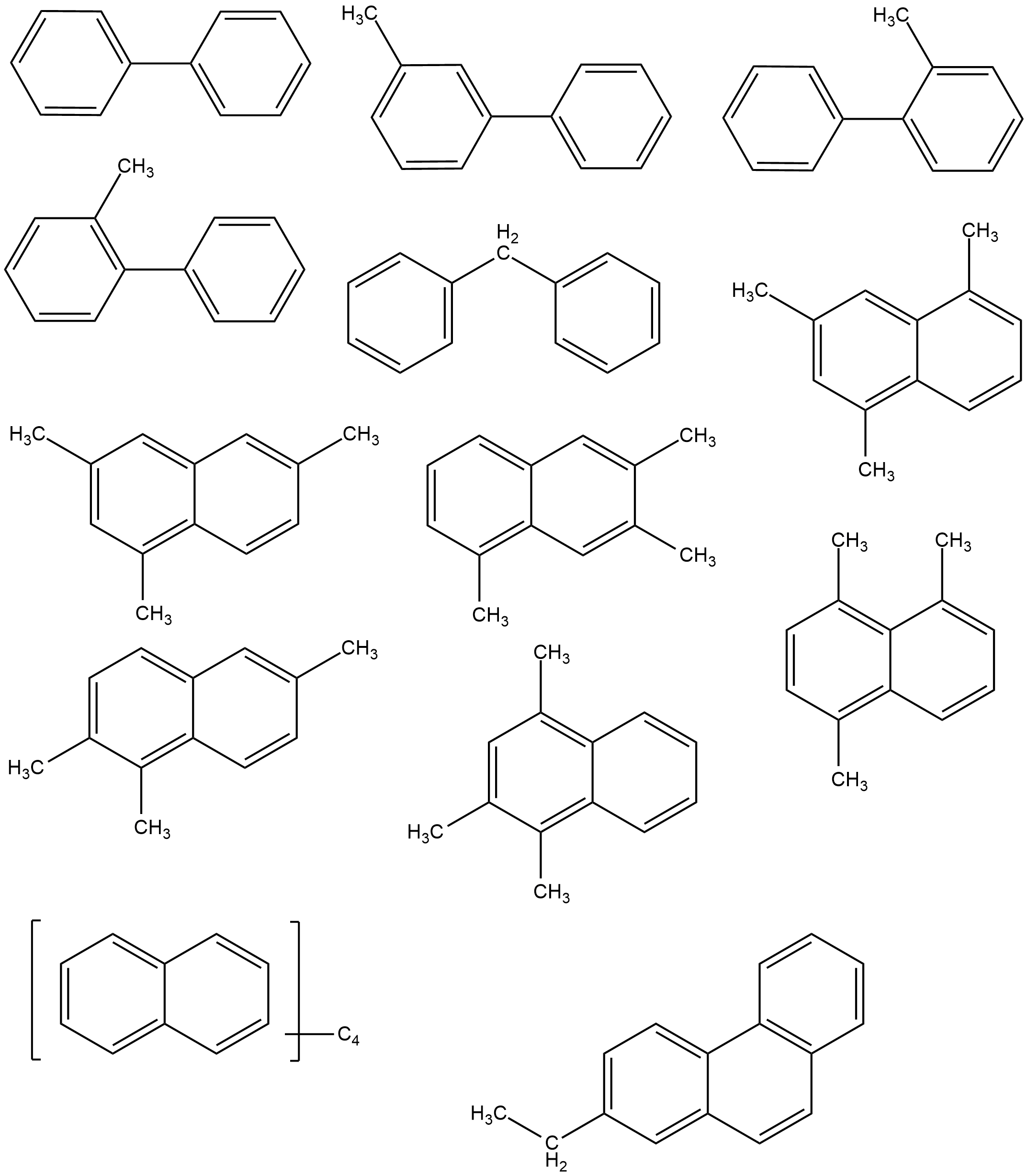Tetramethylnaphthalene (TeMN) |  |  |

**S1 Fig.**

**
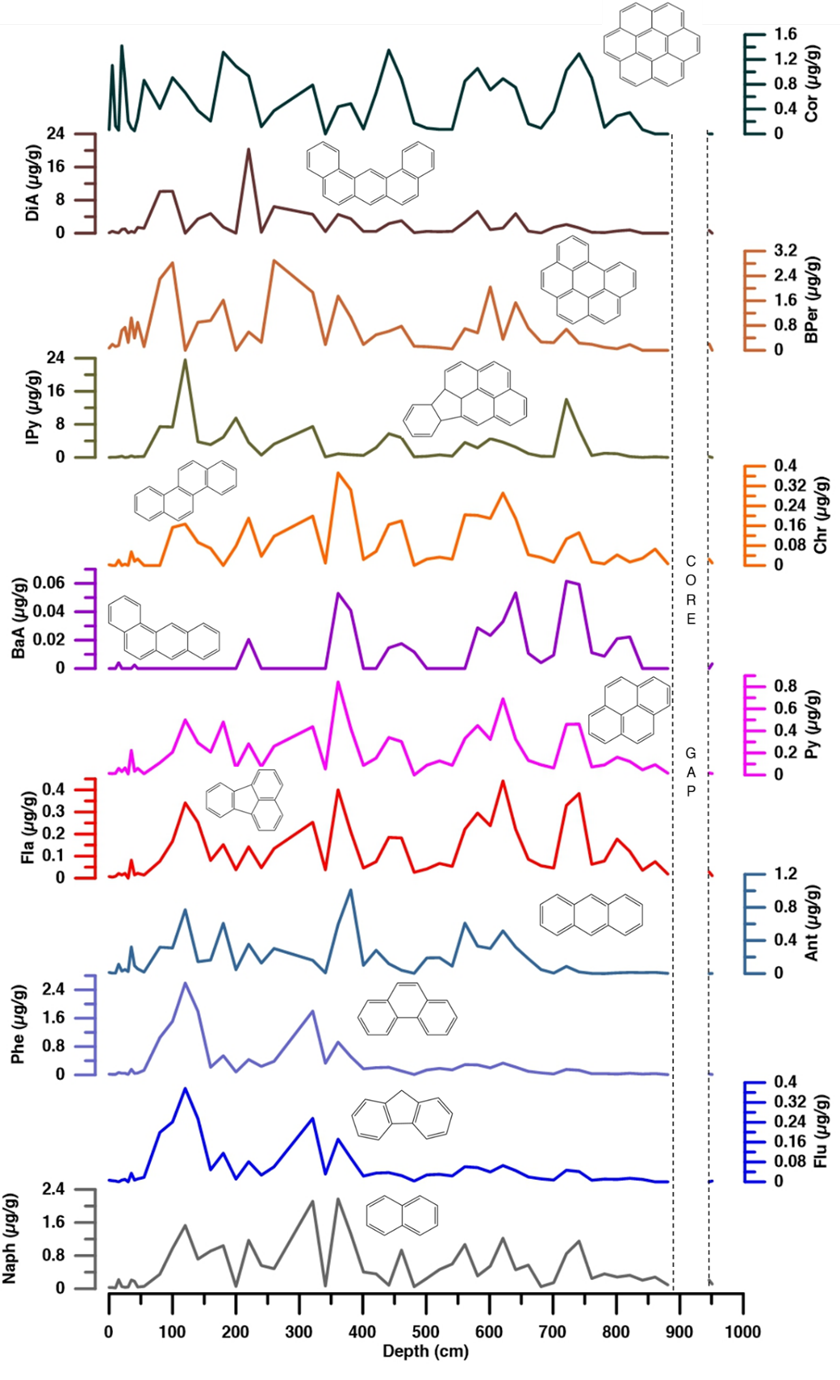
**

**S2 Fig.**

**
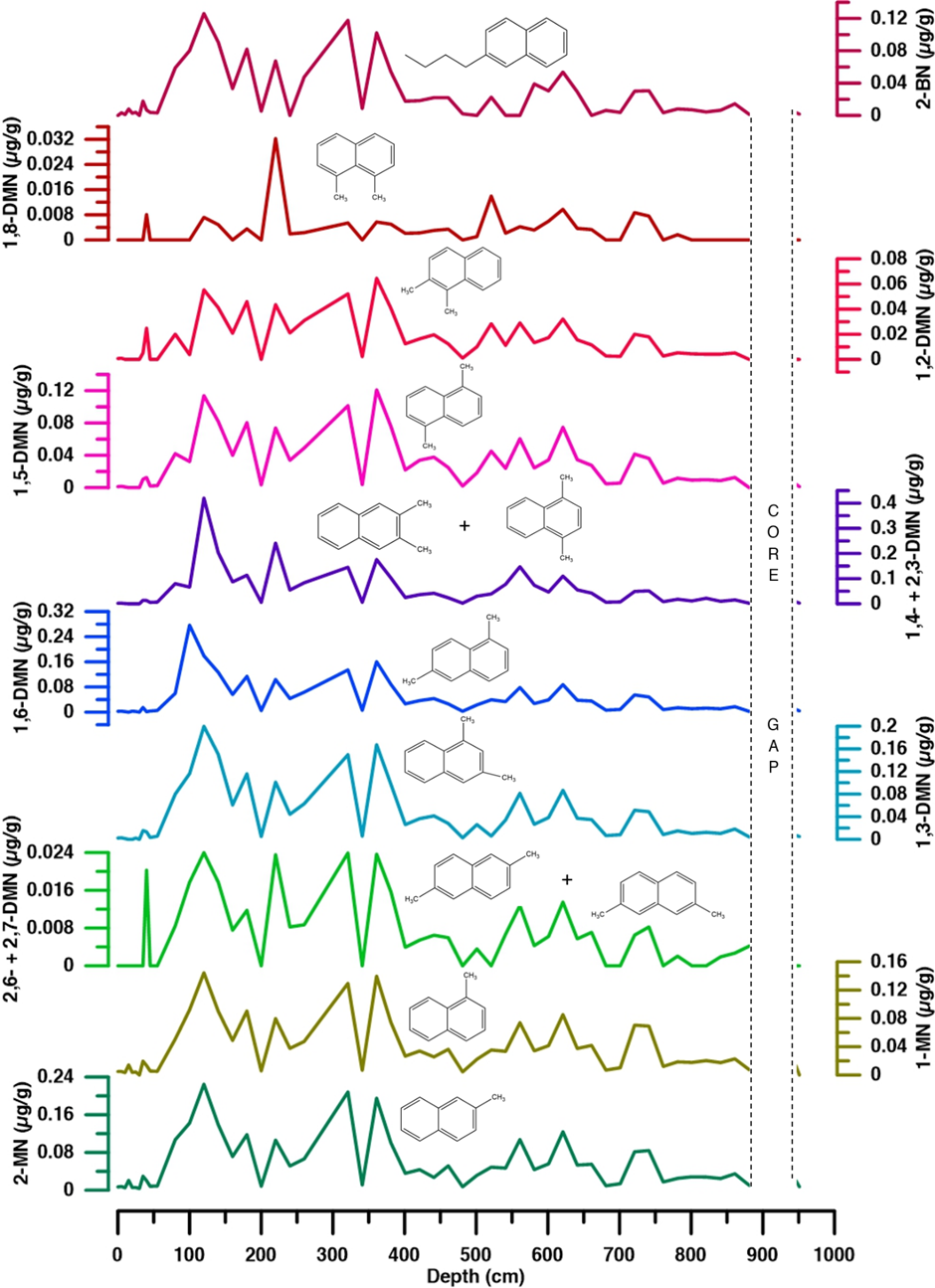
**

**S2 Fig. (continue)**

**
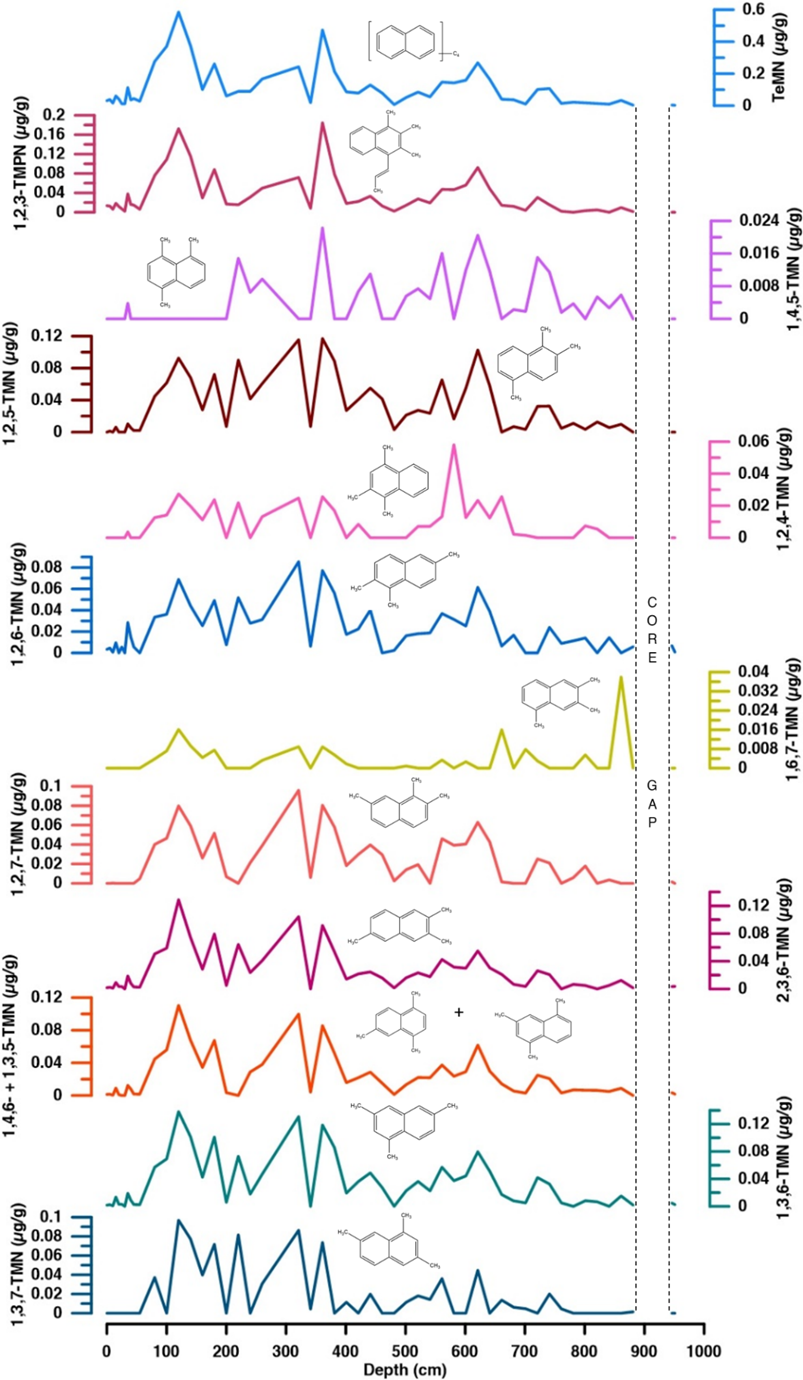
**

**S3 Fig.**

**
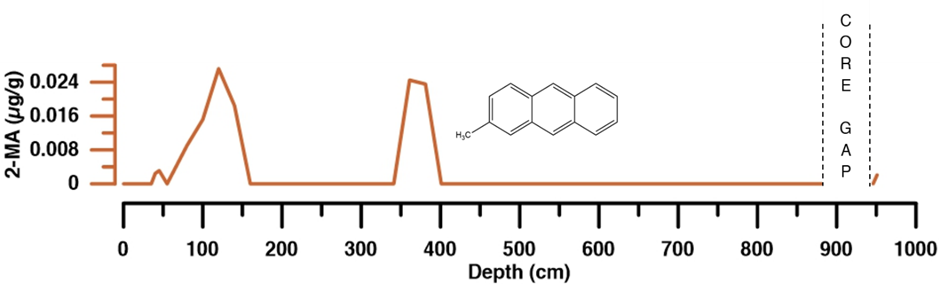
**

**S4 Fig.**

**
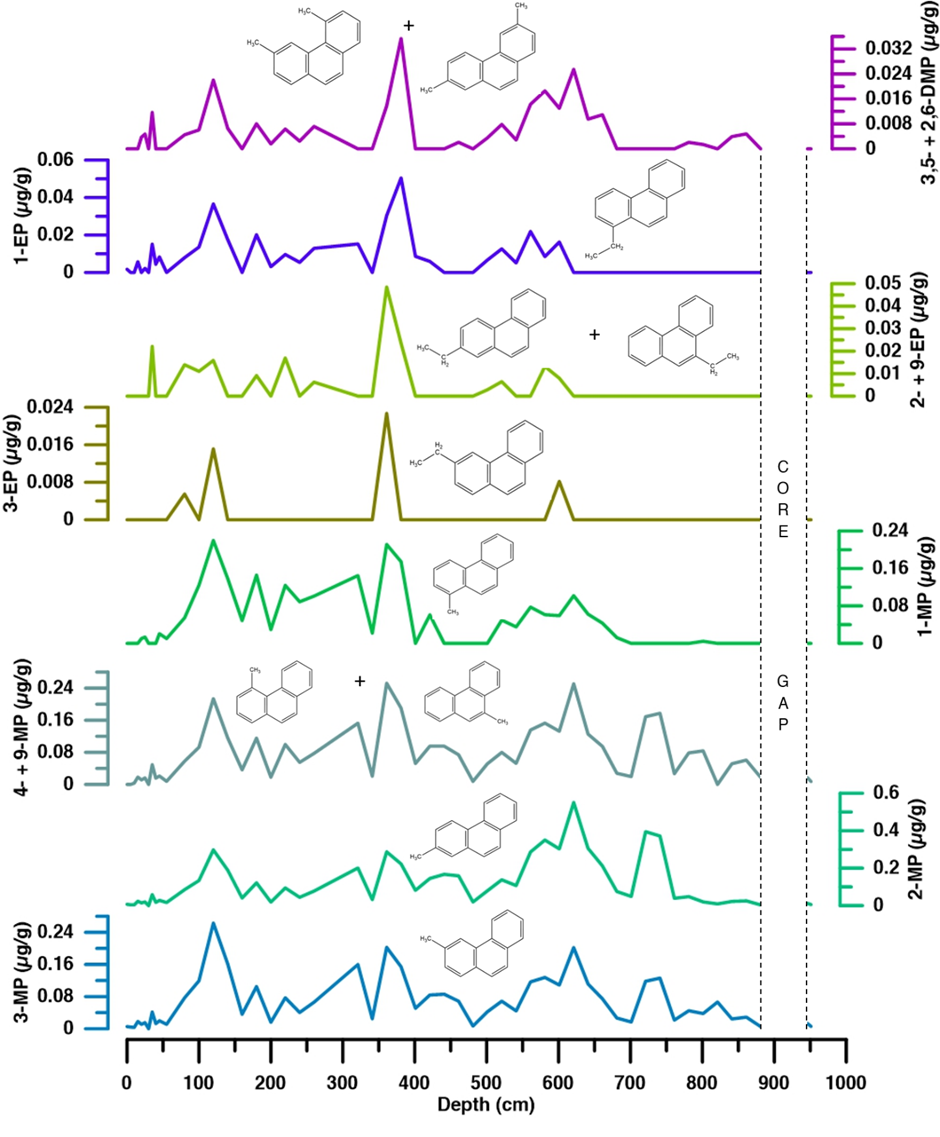
**

**S4 Fig. (continue)**

**
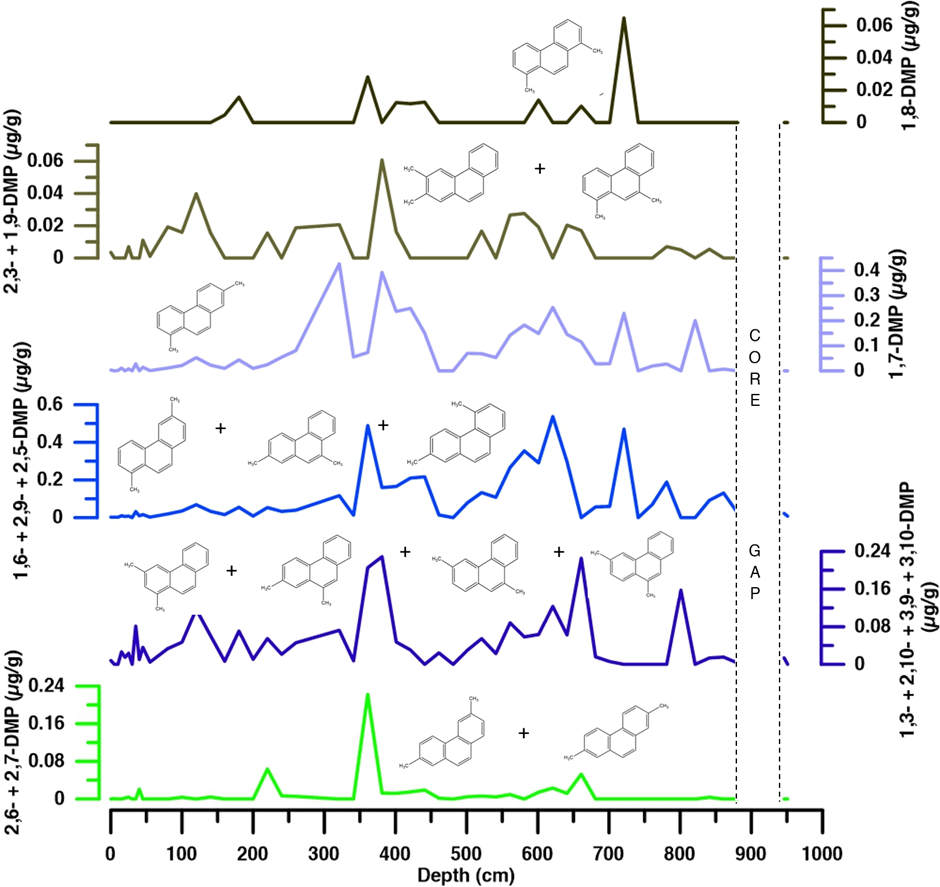
**

**S5 Fig.**

**
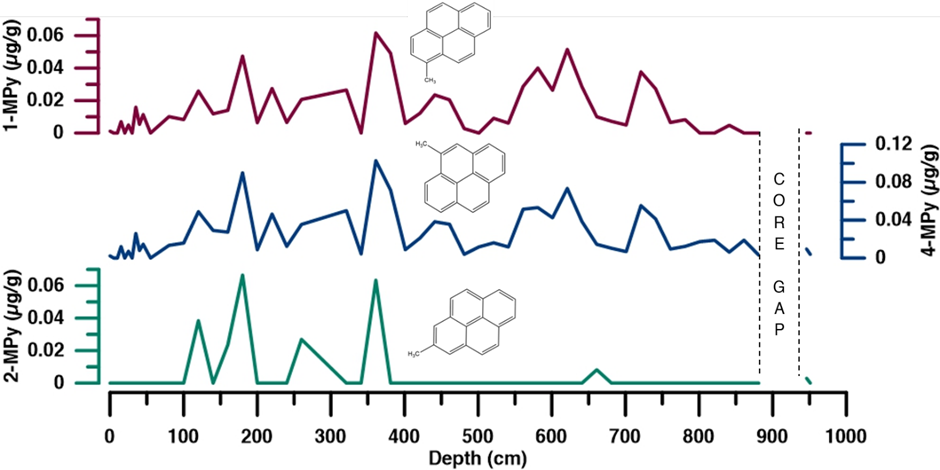
**

**S6 Fig.**

**
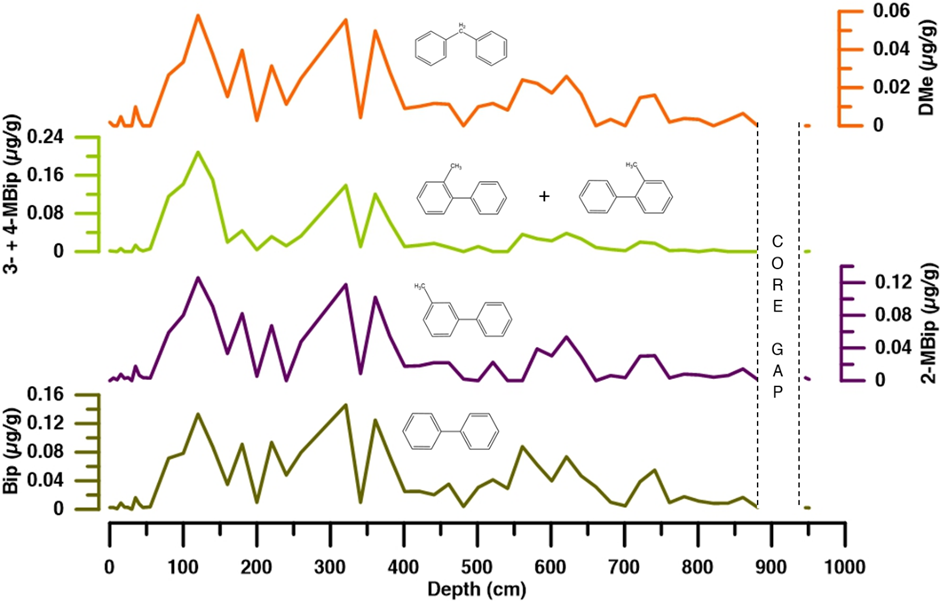
**

**S7 Fig.**

**
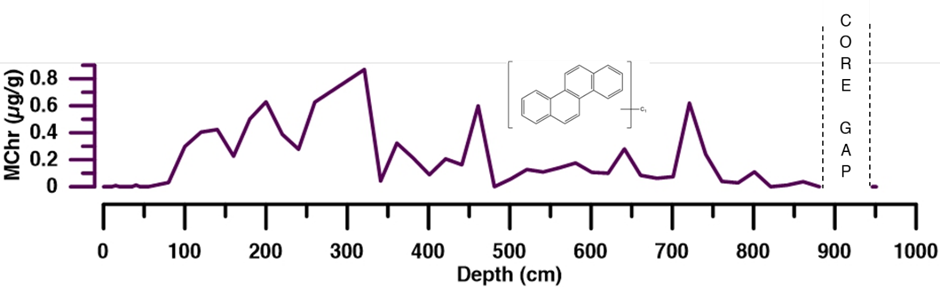
**

**S8 Fig.**


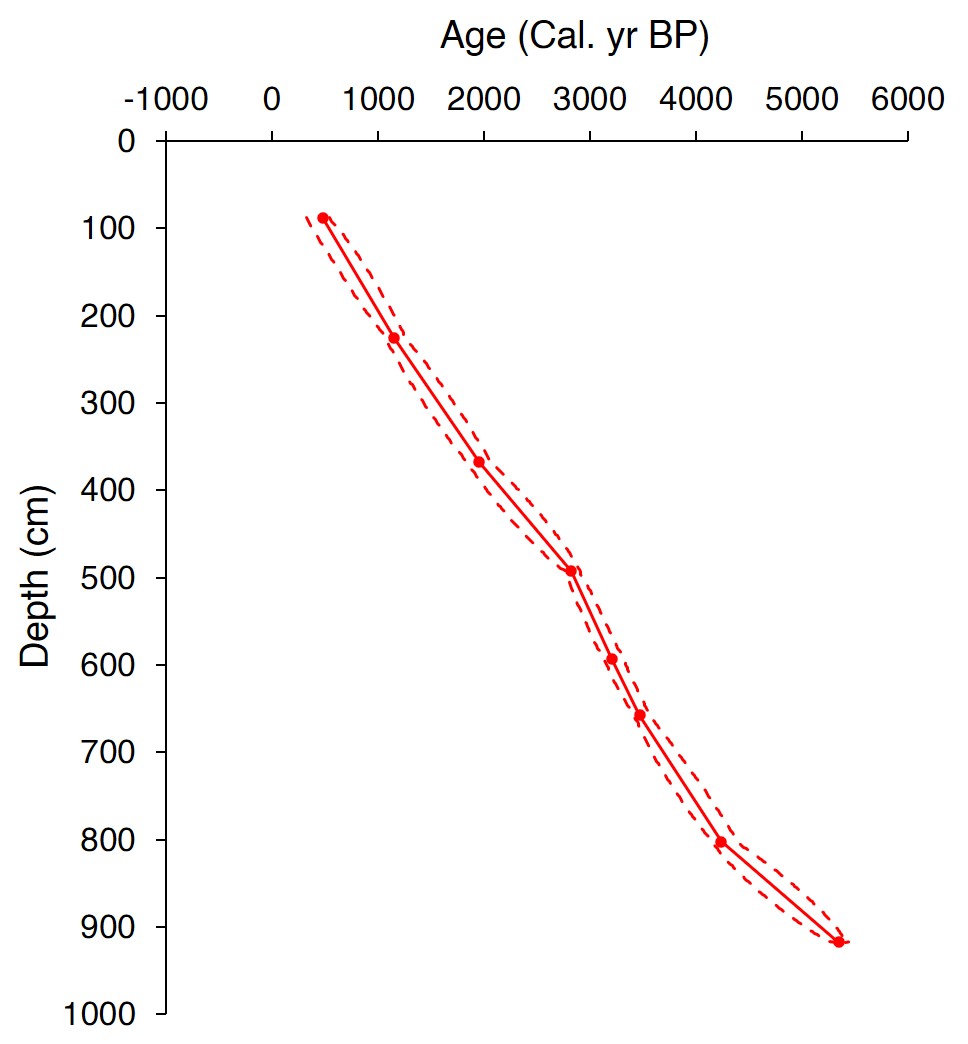

Supplement: S1 File — (DOCX) [file pone.0256853.s001.docx]
